# Analytical approach to structural chemistry origins of mechanical, acoustical and thermal properties

**DOI:** 10.1093/nsr/nwae269

**Published:** 2024-08-01

**Authors:** Zhiwei Chen, Wei Liu, Bing Shan, Yanzhong Pei

**Affiliations:** Interdisciplinary Materials Research Center, School of Materials Science and Engineering, Tongji University, Shanghai 201804, China; Interdisciplinary Materials Research Center, School of Materials Science and Engineering, Tongji University, Shanghai 201804, China; Interdisciplinary Materials Research Center, School of Materials Science and Engineering, Tongji University, Shanghai 201804, China; Interdisciplinary Materials Research Center, School of Materials Science and Engineering, Tongji University, Shanghai 201804, China

**Keywords:** material property prediction, analytical model, electrostatic interaction

## Abstract

Crystalline matters with periodically arranged atoms found wide applications in modern science and technology. To facilitate the design of new materials and the advancement of existing ones, accurate and efficient models without relying too much on known inputs for predicting the functionalities are essential. Here, we propose an analytical approach for such a purpose, with only the knowledge of the structural chemistry of crystals. Based on the electrostatic interaction between periodically arranged atoms, the 1st, 2nd and 3rd derivatives of interatomic potential, respectively, enable a prediction of ten kinds in total of mechanical, acoustical and thermal properties. Over a thousand measurements are collected from ∼500 literatures, this results in the symmetric mean percentage error (SMPE) within ±25% and the symmetric mean absolute percentage error (SMAPE) ranging from 22%∼74% across all properties predicted, which further enables a revelation of bond characteristics as the most important but implicit origin for functionalities.

## Teaser

Based on the electrostatic interaction between periodically arranged atoms, this work develops an analytical approach for predicting mechanical, acoustical and thermal properties of crystals using ICSD data as the only input.

## INTRODUCTION

Electrostatic interactions are ubiquitous in nature, having a vital significance in the formation of condensed matters. They provide robust interaction force among various charged species, such as elementary particles, molecules and proteins [1]. These microscopic forces largely determine the macroscopic properties of matter. For instance, sound waves (mechanical waves) in a one-dimensional oscillator are generated and transmitted by forces based on Hooke's law, while thermal resistance is the result of anharmonicity which is the high-order derivative of the interatomic forces. Therefore, a deeper understanding of electrostatic forces could shed new light on materials’ properties ranging from structure formation to lattice dynamics, help improve existing functional materials and design new ones.

Macroscopic functionality of materials mainly originates from the microscopic functional units and the structure [2]. At a microscopic level, lattice, charge, orbital and spin are the fundamental units [3], and each of them dominates the corresponding electrical and magnetic properties. Moreover, interactions between different units give birth to many unique properties, e.g. superconductivity [4] and thermoelectricity [5] through electron-phonon coupling. In fact, interatomic force is the key functional unit determining the mechanical, acoustical and thermal properties of materials.

The development of materials can be categorized into two perspectives. One focuses on structural geometry based on point group and space group, leading to the knowledge of the structural chemistry of ∼10^5^ inorganics and 10^6^ organics according to the Inorganic Crystal Structure Database (ICSD) and the Cambridge Crystallographic Data Centre (CCDC) databases. The other is a chemical algebraic approach based on quantum [6] or classical mechanics [7,8] for understanding the functionality. However, knowledge of functionality is far behind that of structural chemistry. Specifically, <5% of known inorganics are documented with their thermal conductivities, which of course limits the exploration of thermal functional materials.

In order to facilitate the manipulation and design of functionalities at equilibrium, one has to know the energy of the system as the first step. Surely, atoms standing at their equilibrium positions in space corresponds to the lowest-energy state, while the difficulty lies in the explicit and accessible expression of microscopic interatomic energy. Quantitatively, first-principle calculations enable an estimation of the relationship between structure, chemistry, energy and property. However, it is still challenging in accuracy, cost-efficiency and productivity particularly for complex structures [9–11].

To balance the efficiency and accuracy of predictions, many approximations and models have been developed. For example, the L-J [12], EAM [13,14], Tersoff [15] and machine leaning potentials [16] give the effective potential based on the known binding energy; the Griffith model [17] gives the theoretical fracture strength based on the known surface energy; the Slack [18] model and Synder model [19] give the lattice thermal conductivity based on the known sound velocity and anharmonicity. However, these approaches largely depend on the amount and quality of known inputs and involve numerical fittings [20]. Otherwise, the predictions could tend to be much less-accurate or even physically less-meaningful. Therefore, there is always a desire for an efficient and accurate approach for predictions without many material-dependent inputs and fittings.

**Figure 1.**
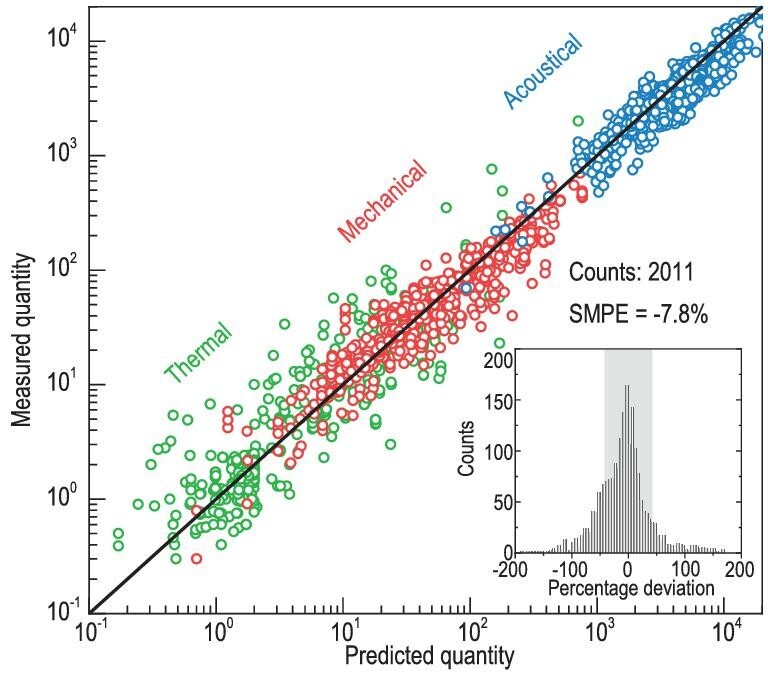
Measurements vs. model predictions for mechanical, acoustical and thermal properties in crystals (detailed units in Fig. 3), along with the corresponding statistic error analyses.

In this work, we develop an analytical model for predicting mechanical, acoustical and thermal functionalities of crystals, using MS Excel as the calculation tool and using the knowledge of structural chemistry from ICSD as the only input. Over 2000 measurements involving ten kinds of properties (mostly at room temperature), are collected from ∼500 literatures, the symmetric mean percentage error (SMPE) of −7.8% and the symmetric mean absolute percentage error (SMAPE) of 32% validate the prediction accuracy (Fig. 1).

**Figure 2.**
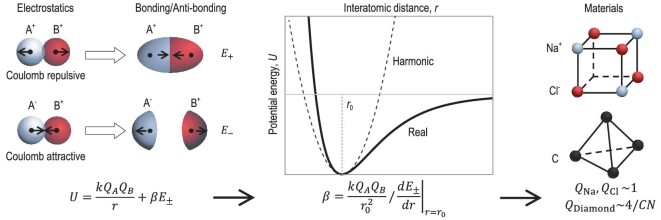
Development of analytical model. Schematic of the analytical potential based on the electrostatic interactions. The first step is to approximate Coulomb/exchange/overlap integrals by analytical function *E* according to the molecular orbital theory. The pre-factor *β* can then be solved according to the equilibrium condition and the electrostatic charge *Q* can be parameterized using basic structural chemistry parameters.

**Figure 3.**
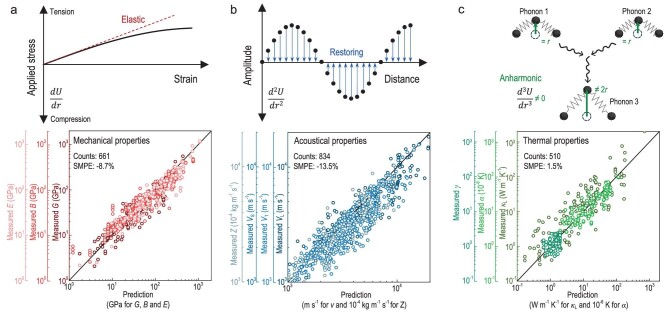
Prediction of properties. Schematic of the differentiations of potential energy, which determine the mechanical (a), acoustical (b) and thermal properties (c), along with a comparison of measurements (detailed ∼500 references are given in Supplementary Materials).

**Figure 4.**
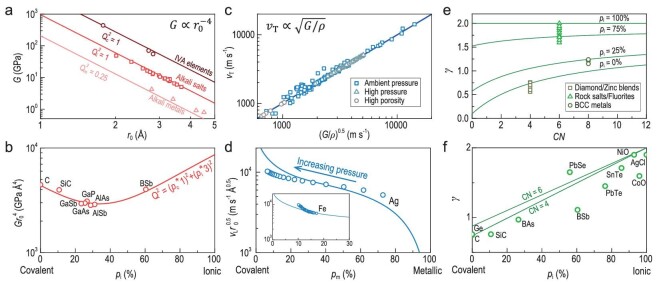
Key structural chemistry parameter for functionalities. Structural-parameter-dependent measured (scatters) and predicted (curves) shear modulus (a and b), sound velocity (c and d) and Grüneisen parameter (e and f).

For each of the predicted properties, the resultant SMPE are within ±25% and SMAPE range from 22%∼74%. Based on the electrostatic interaction between ionic cores, this model approximates the Coulomb, exchange and overlap integrals by analytical functions (Fig. 2). Holding the atom-arrangement periodicity in crystalline matters for simplicity in interatomic potential, the first-, second- and third derivatives of which enable many lattice-dynamic related properties including elastic constants, sound velocities, Grüneisen parameter, linear thermal expansion coefficient, etc. (Fig. 3). Importantly, this approach reveals the key structural chemistry parameters for manipulating each individual functionality (Fig. 4).

## RESULTS AND DISCUSSIONS

### The analytical model

To establish an analytical model (details in Supplementary Section 1), the following approximations are taken in this work: first, a chemical bond is divided into ionic (*U*_i_), covalent (*U*_c_) and metallic (*U*_m_) components; second, the ionic cores are treated as stationary point charges under the Born-Oppenheimer approximation. In this way, the electrodynamic interaction reduces to electrostatic interaction, and the potential between ionic cores has an analytical form of Coulomb potential, as shown in the first terms in Equations 1a, 1b and 1c:


1a \begin{equation*}
{{U}_{\mathrm{i}}} = - {{\alpha }_{\mathrm{i}}}\frac{{k{{Q}_{i,{\mathrm{i}}}}{{Q}_{j,{\mathrm{i}}}}}}{r} + {{\beta }_{\mathrm{i}}}{{e}^{\frac{{ - r}}{{{{r}_0}}}}}\frac{{\mathrm{A}}}{r}
\end{equation*}

1b \begin{equation*}
{{U}_{\mathrm{c}}} = {{\alpha }_{\mathrm{c}}}\frac{{k{{Q}_{i,{\mathrm{c}}}}{{Q}_{j,{\mathrm{c}}}}}}{r} - {{\beta }_{\mathrm{c}}}{{e}^{\frac{{ - r}}{{{\mathrm{C}}{{r}_0}}}}}\left[ {r + {{r}_0}\left( {\frac{{CN}}{{\mathrm{B}}} + {\mathrm{C}}} \right)} \right]
\end{equation*}

1c \begin{equation*}
{{U}_{\mathrm{m}}} = {{\alpha }_{\mathrm{m}}}\frac{{k{{Q}_{i,{\mathrm{m}}}}{{Q}_{j,{\mathrm{m}}}}}}{r} - {{\beta }_{\mathrm{m}}}{{e}^{\frac{{ - r}}{{{\mathrm{C}}{{r}_0}}}}}\left[ {r + {{r}_0}\left( {\frac{{CN}}{{\mathrm{B}}} + {\mathrm{C}}} \right)} \right)\Big]
\end{equation*}

where *k* is the Coulomb constant, *CN* is the coordination number, *r* is the interatomic distance and *r*_0_ is the equilibrium bonding length. The Madelung constant (*α*_i_) is a geometry factor, which is used for determining the superposition of the long-range electrostatic interactions in the lattice. Similarly, the other geometry factors (*α*_c_ for covalent bond and *α*_m_ for metallic bond) are defined in this work for determining short-range electrostatic interactions. Due to the short range, one can approximately consider only the repulsion between the nearest neighbors in which each atom has a *CN* nearest to the neighboring atoms. Thus, the repulsive potential components are proportional to the *CN*. It should be noted that the chemical bonds are localized in covalent compounds while they are delocalized in metallic compounds. This difference leads the geometry parameter for metallic bonds (*α*_m_) to be further weighted by the *CN*. Eventually, *α*_c _= 10*CN*^2^(100 + 19*CN*) and *α*_m_ = *α*_c_/*CN*. The *Q*_i_, *Q*_c_ and *Q*_m_ are ionic, covalent and metallic components of ionic charge for ionic cores *i* and *j*, respectively (Equation S2).

To avoid the collapse or decomposition of the lattice, the total potential requires a short-range repulsive potential of anti-bonding state (*E*_−_) for ionic component or a long-range attractive potential of bonding state (*E*_+_) for covalent and metallic components. Learning from the Coulomb, exchange and overlap integrals of hydrogen molecule ions [21], *E*_±_ are approximated to have analytical forms, as given by the second terms in Equations 1a–1c. Based on the exact definitions that alkalis are purely metallic, alkali salts are purely ionic and diamond-structured IVA elements are purely covalent, the constants A, B and C in Equation 1a–1c are respectively determined to be 1, 9 and 10, after fitting potential energy at equilibrium position (Fig. S1).

Meanwhile, the structural factors are normalized using a pre-factor *β*. The total potential energy (*U*) becomes the sum of *βE*_±_ and Coulomb potential between ionic cores (Fig. 2). The pre-factor *β* can then be solved according to the equilibrium conditions (Equation S1): the first and second derivatives of *U* at the equilibrium have, respectively, to be 0 and >0.

Since the three types of strong chemical bonds (ionic, covalent and metallic) are considered in this work, an arbitrary chemical bond is approximated here to be composed only of ionic (*p*_i_), covalent (*p*_c_) and metallic (*p*_m_) components, then the total potential is the summation of the individual contributions. It should be noted here that there is no strict boundary between chemical bonds. According to Pauling and Phillips [22–24], the difference of electronegativity (Δ*χ*) measures the polarity of the chemical bond, while the average of electronegativity ($\bar{\chi }$) measures the delocalization of the bonding electrons. The former determines *p*_i_ and *p*_c_, and the latter determines *p*_c_ and *p*_m_. Noted here that the bonding electrons are shared among coordination number bonds in crystals. Consequently, *p*_i_, *p*_c_ and *p*_m_ could be obtained according to Equation S3.

With basic structural chemistry parameters including atomic radius, equilibrium bond length (*r*_0_) and coordination number (*CN*) and so on, potential function and the charge *Q* of ionic cores at equilibrium can be determined (Fig. S2). To simplify the derivatives process of the potential energy in this work (Fig. S3), the lattice structure for modeling is approximated to be simple cubic, which is similar to the real structures for IV elements (C, Si and Ge), III-V semiconductors (GaAs, GaSb, etc.), alkali salts (NaCl, KCl, etc.) and alkali metals (Li, Na, K, etc.).

### Measurements *vs.* predictions

The relationship between interatomic force (stress) and distance (strain) can be obtained by taking the first-order derivatives of the potential energy (Fig. 3a), of which the slope is identified as elastic modulus or elastic force constant around the equilibrium position *r*_0_. In this work, elastic (*E*), shear (*G*) and bulk (*B*) modulus are predicted. For simplicity, we use fourth-order stiffness tensor *C_ijkl_* [25] to quantify these moduli by considering the nearest and the next nearest neighbor interactions with a cubic lattice approximation. According to the Voigt and Reuss methods [26], *E*, *G* and *B* can be derived from three independent components (*C*_11_, *C*_12_ and *C*_44_) of the stiffness tensor (Section S2). It should be noted that the observed deviations between the predicted values and the experimental results primarily stem from the discrepancy between the approximated cubic structure of the current model and the actual material structures. For example, alumina and its analogs (e.g. Fe_2_O_3_, Sc_2_O_3_, etc.) crystallize in R3-CH structure. This structure induces extra elements of *C*_13_ and *C*_44_ for the stiffness tensor in addition to *C*_11_, *C*_12_ and *C*_44_ as given by Equation S5. In this case, it is reasonable to expect deviations when estimating the mechanical properties of a hexagonal structure using the stiffness tensor of a cubic structure.

The second-order derivative of the potential energy is the elastic restoring force constant of a vibrating object, which describes the propagation of sound waves through a continuous medium (Fig. 3b). The propagating speed of sound energy (sound velocity, *v*) [27] is highly anisotropic even in a cubic structure, because the bonding strength is highly directional. For comparison (Section S2), predictions and measurements for *v* in polycrystals are included in Fig. 3b. The transverse (*v*_T_) and longitudinal (*v*_L_) sound velocities depend on both the nearest-neighbor (*f*_1_) and next-nearest-neighbor (*f*_2_) force constant. For a cubic approximation, the *v*_T_ is roughly half of the *v*_L_, because *f*_1_ is 8^0.5^ times *f*_2_ based on Equations S4 and S10. The harmonic mean sound velocity [28] (*v*_s_) is defined by 3*v*_s_^−3 ^= *v*_L_^−3 ^+ 2*v*_T_^−3^, which is formulated as Equation 2. In addition, acoustic impedance [29] (*Z*) that is analogous to electrical impedance, can be predicted according to the product of mass density and *v*_L_.


2 \begin{equation*}
{{v}_{\mathrm{S}}} = 0.76\sqrt {\frac{{\frac{\alpha }{2}k{{Q}_{i,{\mathrm{i}}}}{{Q}_{j,{\mathrm{i}}}}\! +\! CNk{{Q}_{i,{\mathrm{c}}}}{{Q}_{j,{\mathrm{c}}}}\! +\! k{{Q}_{i,{\mathrm{m}}}}{{Q}_{j,{\mathrm{m}}}}}}{{{{r}_0}}}}
\end{equation*}

Anharmonicity is the non-zero third-order and higher-order derivatives of potential energy. This results in a non-linear combination of amplitude during phonon collisions, which determines the phonon scattering and thermal properties of crystals [30] (Fig. 3c). Grüneisen parameter [$\gamma = - ( {{{r}_0}/2} )\ ( {\dddot U/\ddot{U}} )]$at *r *= *r*_0_ measures the anharmonicity, which is also crucial for thermal expansion (*α*_T_) and lattice thermal conductivity (*κ*_L_). The linear thermal expansion coefficient (*α*_T_) is determined by ${{\alpha }_{\mathrm{T}}} = ( {\gamma /B} )\ ( {{{C}_{\mathrm{V}}}/V} )/3$ according to the Grüneisen law [31], where *C*_V_ is the heat capacity at constant volume and *V* is the average atomic volume. With the isotropic and cubic approximations, *γ *is reduced to a scalar from a tensor due to the rotational invariance, which is mainly determined by the fractions of bond characters as well as the coordination number:


3 \begin{equation*}
\gamma = 2{{p}_{\mathrm{i}}} + \left( {{{p}_{\mathrm{m}}} + {{p}_{\mathrm{c}}}} \right)\frac{{200 + 599CN}}{{2000 + 380CN}}
\end{equation*}

For lattice thermal conductivity (*κ*_L_) of a crystalline matter, the phonon gas model is utilized in this work to calculate the lattice thermal conductivity via *κ*_L_ = 1/3*C*_V_*v*_s_^2^*τ*, in which the phonon relaxation time (*τ*) is inversely proportional to the square of the Grüneisen parameter (*γ*^2^).

### Error analysis

Since symmetric mean percentage error (SMPE), symmetric mean absolute percentage error (SMAPE) and the mean absolute percentage error (MAPE) can better illustrate the deviation between predicted values and measured values, we estimate the error analysis for each of the properties based on the definitions of SMPE, SMAPE and MAPE (Equations S12a, S12b and S12c), as shown in Table 1. All of the collected and the predicted data are listed in Tables S1–S3.

**Table 1.** Error analysis of symmetric mean percentage error (SMPE), symmetric mean absolute percentage error (SMAPE) and mean absolute percentage error (MAPE) for each of the properties, namely shear modulus (*G*), bulk modulus (*B*), elastic modulus (*E* ), transverse sound velocity (*v*_T_), longitudinal sound velocity (*v*_L_), average sound velocity (*v*_s_), acoustic impendence (*Z* ), Grüneisen parameter (*γ*), linear thermal expansion coefficient (*α*) and lattice thermal conductivity (*κ*_L_).

Error analysis	*G*	*B*	*E*	*v*_T_	*v*_L_	*v*_s_	*Z*	*γ*	*α*	*κ*_L_
SMPE (%)	−20.1	0.15	−20.2	−18.5	−8.9	−17.9	−13.9	−7.3	−15.0	24.3
SMAPE (%)	35.5	30.2	33.9	24.2	21.9	23.8	25.5	27.6	40.5	73.5
MAPE (%)	52.3	34.1	51.8	31.0	26.0	30.2	25.5	32.2	52.6	113.0

It is important to point out here that the relatively large deviation in *κ*_L_ is related to dynamic factors rather than to the thermoelastic parameters predicted here. In regard to the dynamic factors, we learned from the Born–von Karman boundary condition [32] that the dispersion of acoustic phonons is a sine-type (Equation S11a). This is a good approximation for those materials with large number of atoms in the primitive cell, but might not be applicable for materials with simple structures, such as IV elements and III-V semiconductors.

Consequently, although the sound velocity (*v*_s_) and Grüneisen parameter can be predicted quite well (Fig. S4), the lattice thermal conductivity is statistically underestimated. This is attributed to the underestimation of the contribution of high-frequency acoustic phonons to *κ*_L_. This underestimation can be partially eliminated if a linear-type dispersion is used (Equation S11b). The physical scenario is that the group velocity of high-frequency acoustic phonons is similar to that of low-frequency acoustic phonons, which is consistent with the actual phonon dispersion of IV elements, III-V semiconductors, alkali salts and alkali metals. In this way, the SMPE decreases from 88.2% for sine-type dispersion to 18.3% for linear-type dispersion (Fig. S5).

### Key structural chemistry parameters for functionalities

To better reveal the key structural chemistry parameters for manipulating the material's properties, we quantitatively analyze the implications that affect some mechanical, acoustical and thermal properties. As shown in Fig. 4, chemical bond length (*r*) and characteristics along with charge quantity (*Q*) are important.

Common wisdom of electrostatic interaction is that a low charge quantity and a long equilibrium distance result in a weak interatomic force thus a low elastic module. This is reproduced in Fig. 4a, showing that shear modulus (*G*) is clearly proportional to *r*_0_^−4^ and *Q*^2^ for exemplary purely covalent IVA elements (C, Si and Ge), purely ionic alkali salts (NaCl, KCl, etc.) and purely metallic alkali metals (Li, Na, K, Rb).

Furthermore, the mixture of bond characteristics significantly complicates the chemical environment, leading to a nonlinear variation of shear modulus (normalized by *r*_0_^4^) versus the fraction of ionicity (*p*_i_), as shown in Fig. 4b. This is because the net charges *Q* for both cation and anion are weighted by the degree of the mixture of bond characteristics. The underlying mechanism is that the competition between ionicity and covalency induces extra contributions to bond strength, leading to an existence of a minima in elastic modulus at a certain *p*_i_. This is analogous to the disorder-induced alloy scattering of phonons [33].

The propagation of sound waves is related to the mass (or density *ρ*) of vibrating objects and restoring forces. For a transverse sound wave in a crystalline material, velocity (*v*_T_) is proportional to (*G*/*ρ*)^0.5^ whatever the changes in external pressure [34,35] or porosity [36], as can be seen in Fig. 4c.

The fractions of bond covalency (*p*_c_) and metallicity (*p*_m_) depend on the degree of localization of valence electrons. In Fig. 4d, elemental Ag is taken as an example to understand the effect of bond heterogeneity on sound velocity. High pressure shortens the bond favoring the overlap of 4d and 5s orbitals [37,38], which leads to an increase in covalency. This would eventually enhance the sound velocity (normalized by *r*_0_^0.5^ to rule out the effect of the change in bond length), which is also observed in Fe^31^.

Structural chemistry parameters strongly affect thermal properties of crystals as well. As shown in Fig. 4e, Grüneisen parameter (*γ*) monotonously increases with increasing coordination number (*CN*) at a given fraction of ionicity *p*_i_, since higher *CN* means a larger degree of freedom in anharmonicity (Section S2). In addition, long-range attractive interaction beyond the 1st neighboring atomic distance in ionic compounds induces an extra contribution to the potential energy for deviating from a harmonic case (parabola around *r*_0_, Fig. 2). This would result in an increase in* γ* since ionicity increases (Fig. 4f).

## SUMMARY

Taking electrostatic interaction between periodically arranged atoms as the basis of structural chemistry, this work developed an analytical approach for predicting some mechanical, acoustical and thermal properties of crystals using MS Excel. The collection of over a thousand measurements spanning ten kinds of properties, reaches the symmetric mean percentage error (SMPE) within ±25% and the symmetric mean absolute percentage error (SMAPE) ranging from 22%∼74%, ensuring the revelation of key structural chemistry parameters for many functionalities. The proposed approach is believed to offer great potential for screening and designing materials with expectant lattice dynamics related functionalities.

## Supplementary Material

nwae269_Supplemental_Files
