# Supplementary material for: Analytical approach to structural chemistry origins of mechanical, acoustical and thermal properties
Source: Natl Sci Rev. 2024 Aug 1;11(9):nwae269. doi: 10.1093/nsr/nwae269 (PMC11345612; doi:10.1093/nsr/nwae269)
Supplement: nwae269_Supplemental_Files [file nwae269_supplemental_files.zip › Supplementary data.pdf]

Supplementary

Analytical approach to structural chemistry origins of mechanical, acoustical and thermal properties

Zhiwei Chen, Wei Liu, Bing Shan, and Yanzhong Pei*

Interdisciplinary Materials Research Center, School of Materials Science and Engineering, Tongji Univ., Shanghai 201804, China.

*Email: yanzhong@tongji.edu.cn

Section S1: Model Deduction

As shown in **Figure S1**, Equations 1a-1c are used for fitting the potential energy at equilibrium position for some pure ionic, pure covalent, and pure metallic compounds, which determines the material-independent constants A, B and C respectively.

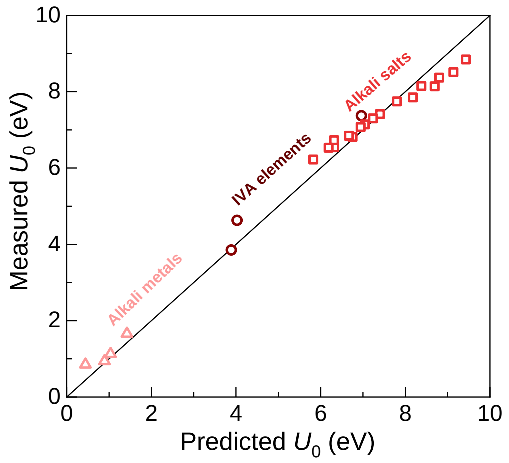

Figure S1. Prediction of the potential energy at equilibrium position for alkali salts, IVA elements and alkali metals, along with a comparison to measurements[1-3].

The pre-factors β_i, β_c and β_m are related to some structural quantities, as mentioned in the main text. If the equilibrium position r_0 is known, the pre-factor can be solved according to the equilibrium condition, where the first derivative of total potential *vs.* r has to be 0. Consequently, the pre-factors are given in **Equation S1a, S1b** and **S1c**.

$$\beta_{\mathrm{i}} = \alpha_{\mathrm{i}} e \frac{kQ_{i,\mathrm{i}}Q_{j,\mathrm{i}}}{2} \tag{S1a}$$

$$\beta_{\mathrm{c}} = \alpha_{\mathrm{c}} \frac{100e^{\frac{1}{10}}}{CN} \frac{kQ_{i,\mathrm{c}}Q_{j,\mathrm{c}}}{r_0^2} \tag{S1b}$$

$$\beta_{\mathrm{m}} = \alpha_{\mathrm{m}} \frac{100e^{\frac{1}{10}}}{CN} \frac{kQ_{i,c}Q_{j,c}}{r_0} \tag{S1c}$$

To determine the charge for ionic cores (Q), the number of bonding electrons (N_b) is firstly estimated. We further assume the electrons of each shell follow a Gaussian distribution in a Bohr atom model, as shown in **Figure S2a**. For an isolated atom with principal quantum number (n), a, b and c in Gaussian function are respectively the number of electrons (N_n), the radius and the distribution range for each shell. For examples, N_n in carbon atom is 2 for n = 1 and is 4 for n = 2. Since each carbon atom in diamond is believed to have exact 4 valent electrons, the distribution range (c) is determined to be 0.07 under a full description of a Gaussian distribution. For simplicity, we therefore use c=0.07 for all orbitals and the radius (b) of shell is considered as equant in this work.

The molecular orbital theory emphasizes the importance of the overlap integral between the two atomic orbitals—the nature of the chemical bond[4]. Due to the orbital overlap between atoms, the number of bonding electrons **(Figure S2b)** is the sum of the integrated areas under the discrete Gaussian functions. For atom i, the integral starts from the bonding radius (r_{ij}) to the infinite, where r_{ij} is determined by the bonding length (r_0) and the atomic radii for atom i and j via $r_{ij} = (r_0 - r_j + r_i)/2$. Similarly, the bonding radius for atom j (r_{ji}) equals to $(r_0 - r_i + r_j)/2$. The ERF in Figure S1b is the abbreviation of error function (also called Gauss error function), which is used for describing the integral of the Gaussian distribution as shown in Figure S1a. Thus, the (1+ERF) is the cumulative distribution function for estimating the number of bonding electrons due to orbital overlap between atoms.

Taking diamond as an example (Figure S1b), the bonding radius is estimated to be ~0.75 Å, which is larger than the upper boundary of 1st shell for carbon (Figure S1a). Therefore, the 1st orbitals do not overlap, and the electrons in 1st orbitals do not participate in bonding. On the other hands, the r_{ij} is smaller than the lower boundary of 2nd orbital, which means all of electrons in the 2nd shell participate in bonding. Accordingly, the number of bonding electrons is estimated to be ~4 for diamond. This further indicates that the higher the degree of orbital overlap, the smaller the bonding radius, and consequently, the greater the number of bonding electrons.

For ionic component, the net ionic charge (Q_i) depends on the charge of anions with larger electronegativity, at the same time, the cations have the equivalent but opposite-sign charges. For an example, 3- for Sb and 3+ for Ga in GaSb. For covalent

component, the directionality of chemical bonds leads the net charges (Q_c) to be weighted by CN. Eventually, Q_i, Q_c, and Q_m can be written as:

$$Q_i = p_i(8 - N_{n\_max}) \quad \text{(S2a)}$$
$$Q_m = p_m N_b \quad \text{(S2b)}$$
$$Q_c = p_m N_b / CN \quad \text{(S2c)}$$

where the fraction of ionicity (p_i), covalency (p_c) and metallicity (p_m) characters are determined by the difference and average values of electronegativity (χ)[5].

$$p_i = 1 - e^{-(\chi_i - \chi_j)^2/4} \frac{N_b}{CN} \quad \text{(S3a)}$$
$$p_m = (1 - p_i)(1 - e^{-\frac{2(\bar{\chi} - 0.79)^2}{CN/N_b}}) \quad \text{(S3b)}$$
$$p_c = 1 - p_i - p_m \quad \text{(S3c)}$$

The above case is applicable for binary compounds of cation to anion molar ratio of 1:1. For binary compounds of cation to anion molar ratio of ≠1, Q_i of the element with higher N_n should be weighted by the subscript of another atom with lower N_n. For examples: 2+ for Mg and 4- over 2 for Pb in Mg_2Pb; 1+ for Li and 2- over 2 for O in Li_2O; 2+ over 2 for Mg and 1- for Cl in $MgCl_2$.

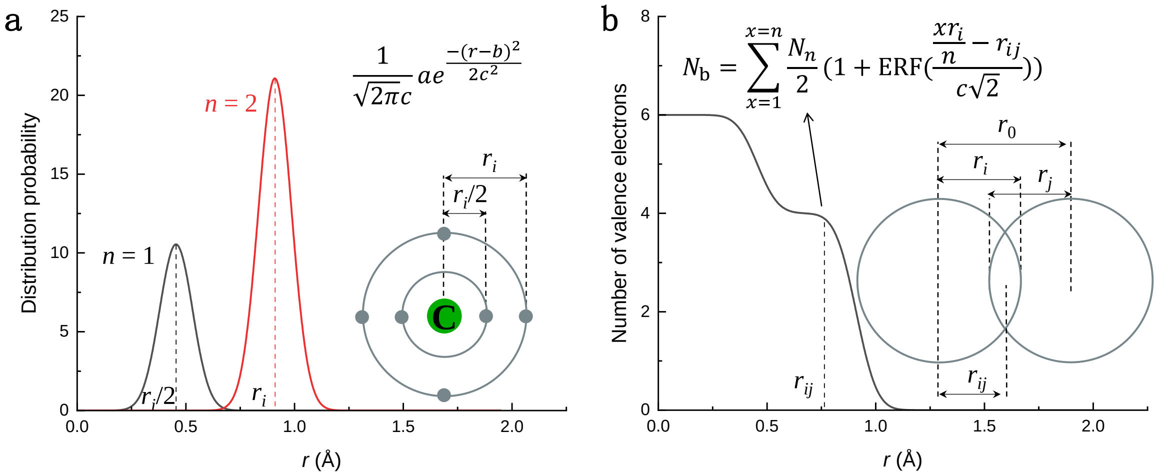

Figure S2. (a) the distribution probability of the extranuclear electron for carbon as an example. (b) the estimation of the number of bonding electrons based on the overlap of orbitals.

Section S2: Model Prediction

Taking NaCl as an example, **Figure S3** displays the zero-, first-, second- and third derivatives of the potential energy *vs.* the interatomic distance, all of which are divided into repulsive (red curves) and attractive parts (blue curves). The properties at equilibrium are obtained according to the equilibrium position r_0, which allows to predict some mechanical, acoustical and thermal properties.

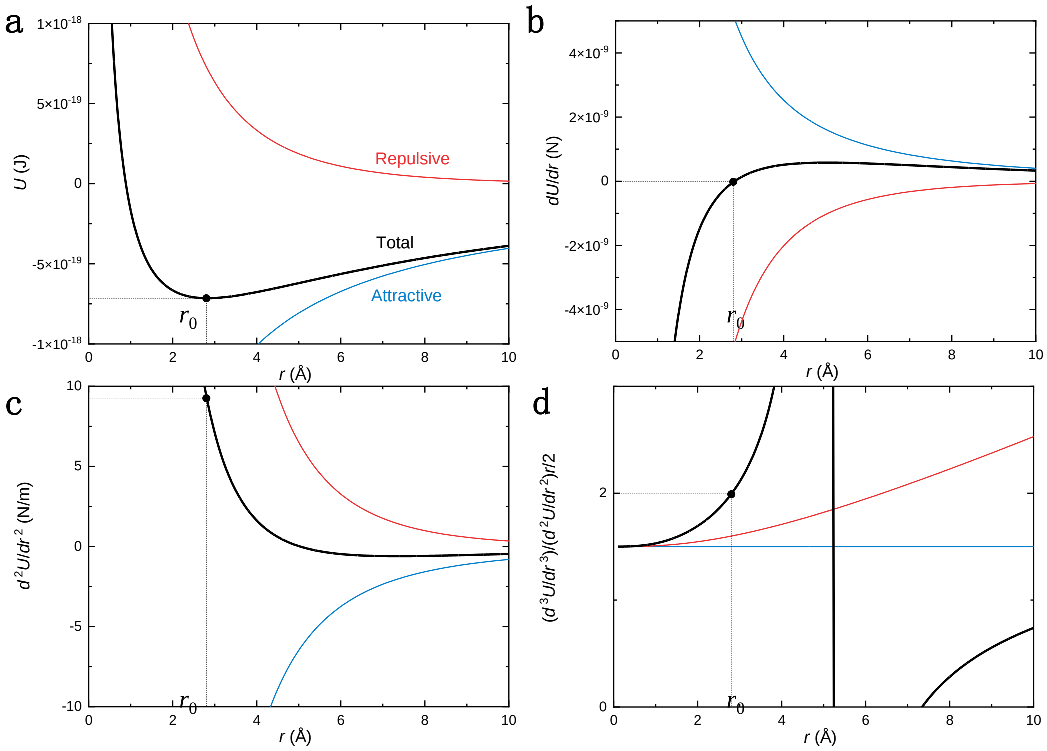

Figure S3. The zero (a), first (b) and second (c) derivatives of potential energy *vs.* the interatomic distance for NaCl. r_0 represents the bond length at equilibrium (first derivative of 0). (d) Grüneisen parameter *vs.* interatomic distance.

At equilibrium, the second-order force constants of ionic (f_i), covalent (f_c) and metallic (f_m) components are given by

$$f_{\mathrm{i}} = \frac{d^2U_{\mathrm{i}}}{dr^2} = \frac{\alpha}{2}\frac{kQ_{i,\mathrm{i}}Q_{j,\mathrm{i}}}{r_0^3} \quad \text{(S4a)}$$

$$f_{\mathrm{c}} = \frac{d^2U_{\mathrm{c}}}{dr^2} = CN\frac{kQ_{i,\mathrm{c}}Q_{j,\mathrm{c}}}{r_0^3} \quad \text{(S4b)}$$

$$f_{\mathrm{m}} = \frac{d^2U_m}{dr^2} = \frac{kQ_{i,\mathrm{m}}Q_{j,\mathrm{m}}}{r_0^3} \quad \text{(S4c)}$$

For a cubic lattice, the force constants between the nearest and next neighbor atoms are defined as f_1 with interatomic distance of r_0 and f_2 with $2^{0.5}r_0$. Thus, f_1 is $8^{0.5}$ times f_2 based on Equation S4. The only three independent tensor C_{11}, C_{12} and C_{44} of the elastic modulus of cubic structure can be derived[6].

$$c_{11} = r_0^2\frac{f_1 + 2f_2}{M}\rho \quad \text{(S5a)}$$

$$c_{12} = r_0^2\frac{f_2}{M}\rho \quad \text{(S5b)}$$

$$c_{44} = r_0^2\frac{f_2}{M}\rho \quad \text{(S5c)}$$

where M is the average atomic mass and ρ is the volume density. According to the average of Voigt approximation and Reuss approximation[7], the Bulk modulus (B), shear modulus (G) and Young modulus (E) are given as

$$B = \frac{c_{11} + 2c_{12}}{3} \quad \text{(S6a)}$$

$$G = \frac{\frac{c_{11} - c_{12} + 3c_{44}}{4} + \frac{5c_{44}(c_{11} - c_{12})}{3(c_{11} - c_{12}) + 4c_{44}}}{2} \quad \text{(S6b)}$$

$$E = \frac{9BG}{3B + G} \quad \text{(S6c)}$$

The longitudinal, transverse and harmonic average sound velocities can be estimated by these elastic properties

$$v_{\mathrm{L}} = \sqrt{\frac{B + \frac{4}{3}G}{\rho}} \tag{S7a}$$

$$v_{\mathrm{T}} = \sqrt{\frac{G}{\rho}} \tag{S7b}$$

$$v_{\mathrm{S}} = [\frac{1}{3}(v_{\mathrm{L}}^{-3} + 2v_{\mathrm{T}}^{-3})]^{-1/3} \tag{S7c}$$

Alternatively, the sound velocity could also so be obtained from the phonon dispersion. According to the Born-Von Karman periodic boundary conditions[8], the phonon dispersions along the [100], [110] and [111] directions are given by Equations S8a, S8b and S8c, respectively.

$$\omega_1^2 = \frac{2}{M}(f_1 + 2f_2)(1 - \cos kr_0),\ \omega_2^2 = \omega_3^2 = \frac{2}{M}f_2(1 - \cos kr_0) \tag{S8a}$$

$$\omega_1^2 = \frac{2}{M}\left[(f_1 + f_2)\left(1 - \cos\frac{kr_0}{\sqrt{2}}\right) + 2f_2\left(\sin\frac{kr_0}{\sqrt{2}}\right)^2\right],$$
$$\omega_2^2 = \frac{2}{M}(f_1 + f_2)\left(1 - \cos\frac{kr_0}{\sqrt{2}}\right), \omega_3^2 = \frac{2}{M}2f_2\left(1 - \cos\frac{kr_0}{\sqrt{2}}\right) \tag{S8b}$$

$$\omega_1^2 = \frac{2}{M}\left[f_1\left(1 - \cos\frac{kr_0}{\sqrt{3}}\right) + 4f_2\left(\sin\frac{kr_0}{\sqrt{3}}\right)^2\right], \omega_2^2 = \omega_3^2 = \frac{2}{M}\left[f_1\left(1 - \cos\frac{kr_0}{\sqrt{3}}\right) + f_2\left(\sin\frac{kr_0}{\sqrt{3}}\right)^2\right] \tag{S8c}$$

where ω is the phonon frequency and k is the phonon wave vector. Taking the derivative of the phonon dispersion at wave vector k=0, we obtain the longitudinal (v_{L}) and transverse (v_{T}) sound velocities along the [100] (Equation S9a), [110] (Equation S9b) and [111] (Equation S9c) directions.

$$v_{\mathrm{L}} = r_0\sqrt{\frac{f_1 + 2f_2}{M}} = r_0\sqrt{\frac{(1 + \sqrt{2})f_1}{\sqrt{2}M}}, v_{\mathrm{T1}} = v_{\mathrm{T2}} = r_0\sqrt{\frac{f_2}{M}} = r_0\sqrt{\frac{f_1}{2\sqrt{2}M}} \tag{S9a}$$

$$v_{\mathrm{L}} = r_0\sqrt{\frac{(4 + 5\sqrt{2})f_1}{8M}}, v_{\mathrm{T1}} = r_0\sqrt{\frac{(4 + \sqrt{2})f_1}{8M}}, v_{\mathrm{T2}} = r_0\sqrt{\frac{\sqrt{2}f_1}{4M}} \tag{S9b}$$

$$v_{\mathrm{L}} = r_0\sqrt{\frac{(1 + 2\sqrt{2})f_1}{3M}}, v_{\mathrm{T1}} = v_{\mathrm{T2}} = r_0\sqrt{\frac{(1 + \sqrt{2})f_1}{3\sqrt{2}M}} \tag{S9c}$$

The different numeric factors indicate the anisotropy of the sound velocity. Consequently, the sound velocity for polycrystals can be written as

$$v_{\mathrm{L}} = 1.19\sqrt{\frac{\frac{\alpha}{2}kQ_{i,\mathrm{i}}Q_{j,\mathrm{i}} + CNkQ_{i,\mathrm{c}}Q_{j,\mathrm{c}} + kQ_{i,\mathrm{m}}Q_{j,\mathrm{m}}}{r_0}} \tag{S10a}$$

$$v_{\mathrm{T}} = 0.69\sqrt{\frac{\frac{\alpha}{2}kQ_{i,\mathrm{i}}Q_{j,\mathrm{i}} + CNkQ_{i,\mathrm{c}}Q_{j,\mathrm{c}} + kQ_{i,\mathrm{m}}Q_{j,\mathrm{m}}}{r_0}} \tag{S10b}$$

$$v_{\mathrm{S}} = 0.76\sqrt{\frac{\frac{\alpha}{2}kQ_{i,\mathrm{i}}Q_{j,\mathrm{i}} + CNkQ_{i,\mathrm{c}}Q_{j,\mathrm{c}} + kQ_{i,\mathrm{m}}Q_{j,\mathrm{m}}}{r_0}} \tag{S10c}$$

At the high-temperature limit, contributions of acoustic (κ_{a}) and optical (κ_{o}) phonons to lattice thermal conductivity (κ_{L}) under a Umklapp scattering can be determined by the equations with (sine-type dispersion, Equation S11a[9]) or without (linear-type dispersion, Equation S11b[10]) Born-von Karman boundary condition,

$$\kappa_{\mathrm{L,sine}} = \kappa_{\mathrm{a}} + \kappa_{\mathrm{o}} = \frac{1}{3\pi^3}(\frac{6\pi^2}{V}\frac{1}{N})^{2/3}\frac{v_{\mathrm{S}}^3M}{\gamma^2T} + \frac{3k_{\mathrm{B}}}{2}\left(\frac{\pi}{6V^2}\right)^{\frac{1}{3}}(1 - \frac{1}{N^{2/3}})v_{\mathrm{S}} \tag{S11a}$$

$$\kappa_{L,\mathrm{linear}} = \kappa_a + \kappa_o = \frac{1}{4\pi^2}(\frac{6\pi^2}{V}\frac{1}{N})^{2/3}\frac{v_S^3M}{\gamma^2T} + \frac{3k_B}{2}\left(\frac{\pi}{6V^2}\right)^{\frac{1}{3}}(1 - \frac{1}{N^{2/3}})v_S \tag{S11b}$$

where N is the number of atoms in the primitive cell, V is the average atomic volume. As shown in Figure S4, we can find that the predictions for IV elements (C, Si and Ge), III-V semiconductors (GaAs, GaSb, etc.), alkali salts (NaCl, KCl, etc.) and alkali metals (Li, Na, K, etc.) is quite good, showing percentage errors <5%.

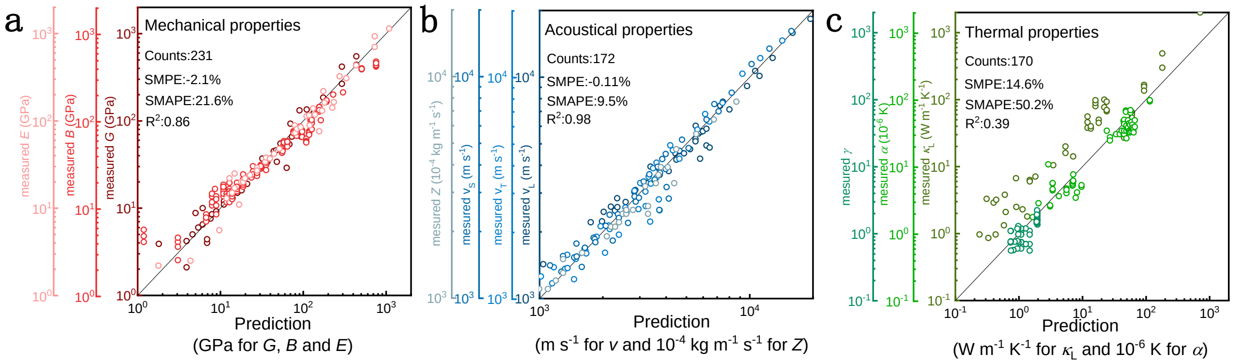

Figure S4. Predictions of mechanical (a), acoustical (b) and thermal (c) properties, along with a comparison to measurements for IV elements, III-V semiconductor, alkali salts and alkali metals.

Based on Equation S11a and S11b, with the same input acoustic and thermoelastic properties, the differences in lattice thermal conductivity predictions primarily stem from the different dispersion of acoustic phonons.

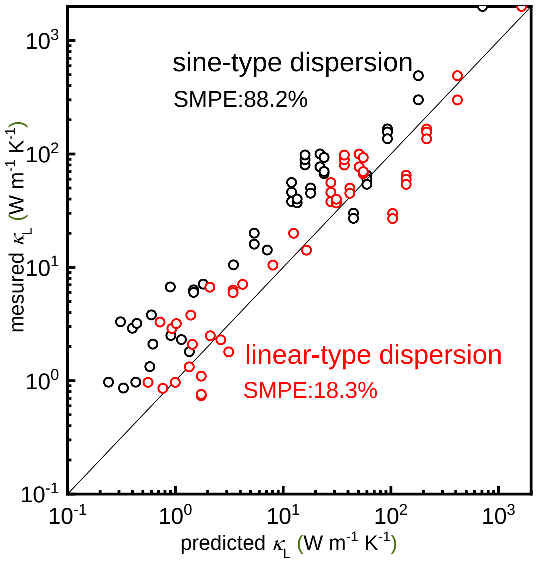

Figure S5. Comparison of the predictions of lattice thermal conductivity for sine-type dispersion [ref] and linear-type dispersion.

In order to evaluate the accuracy of the predictions, the symmetric mean percentage error (SMPE, Equation S12a), the symmetric mean absolute percentage error absolute deviation (SMAPE, Equation S12b) and the mean absolute percentage error (MAPE, Equation S12c) are used for the error analyses (Table 1)

$$\mathrm{SMPE} = \frac{100\%}{n}\sum_{i=1}^{n}\left|\frac{meas._i - pred._i}{meas._i}\right| \tag{S12a}$$

$$\mathrm{SMAPE} = \frac{100\%}{n}\sum_{i=1}^{n}\left|\frac{meas._i - pred._i}{(meas._i + pred._i)/2}\right| \tag{S12b}$$

$$\mathrm{MAPE} = \frac{100\%}{n}\sum_{i=1}^{n}\left|\frac{meas._i - pred._i}{meas._i}\right| \tag{S12c}$$

Table S1. The predicted mechanical properties based on the model proposed in this work, along with a comparison to measurements. *G* is the shear modulus, *B* is the bulk modulus and *E* is the Young modulus.

Materials	Pred. *G* (GPa)	Meas. *G* (GPa)	Pred. *B* (GPa)	Meas. *B* (GPa)	Meas. *E* (GPa)	Pred. *E* (GPa)
Diamond	437	440[11]	767	429[12]	1101	1142[11]
		550[13]		437[14]		1186[13]
				490[15]		
				445[16]		
				469[13]		
Si	73	66[17]	128	98[14]	183	162[17]
				82[17]		
				96[18]		
				105[19]		
Ge	68	54[20]	119	74[18]	171	131[20]
				78[21]		
				76[22]		
				75[23]		
				71[18]		
3C-SiC	174	185[24]	306	217[24]	440	433[24]
c-BN	293	418[25]	514	410	738	931[25]
		266[26]		399[27]		703[26]

Materials	Pred. *G* (GPa)	Meas. *G* (GPa)	Pred. *B* (GPa)	Meas. *B* (GPa)	Meas. *E* (GPa)	Pred. *E* (GPa)
				401[28]		
				403[29]		
BP	110	135[30]	193	169[30]	278	324[30]
				172[31]		
				171[32]		
				176[33]		
BAs	90	132[34]	158	131[35]	227	142[34]
				148[36]		
				147[33]		
				136[37]		
BSb	70	93[38]	124	108[39]	177	219[38]
				116[38]		
				103[27]		
				115[40]		
AlN	133	123[25]	233	205[25]	335	308[25]
AlAs	44	45[41]	77	78[42]	111	113[41]
				76[43]		
				74[37]		

Materials	Pred. *G* (GPa)	Meas. *G* (GPa)	Pred. *B* (GPa)	Meas. *B* (GPa)	Meas. *E* (GPa)	Pred. *E* (GPa)
AlSb	34	32[43]	59	58[43]	85	81[43]
				60[42]		
				57[43]		
				58[42]		
GaP	53	55[44]	92	88[44]	133	139[44]
				87[43]		
GaAs	47	46[45]	83	76[43]	119	116[45]
				75[46]		
				73[47]		
GaSb	37	34[43]	65	57[43]	94	85[43]
				52[48]		
				60[49]		
				59[50]		
InN	94	66[25]	165	104[25]	237	164[25]
InP	48	34[51]	84	70[49]	121	89[51]
				69[52]		
				81[53]		
				76[32]		

Materials	Pred. *G* (GPa)	Meas. *G* (GPa)	Pred. *B* (GPa)	Meas. *B* (GPa)	Meas. *E* (GPa)	Pred. *E* (GPa)
InAs	63	29[54]	110	64[32]	158	76[54]
				60[43]		
				59[52]		
				58[55]		
InSb	32	29[56]	57	49[43]	81	73[56]
				47[57]		
				46[42]		
BeO	174	156[58]	306	252[59]	439	403[59]
		163[59]		226[60]		417[61]
				283[62]		
MgO	158	130[63]	277	155[63]	397	305[63]
				162[64]		
				157[65]		
CaO	97	81[66]	170	117[66]	244	198[66]
				119[67]		
				109[65]		
				122[68]		
SrO	74	59[63]	130	88[63]	186	173[63]

Materials	Pred. *G* (GPa)	Meas. *G* (GPa)	Pred. *B* (GPa)	Meas. *B* (GPa)	Meas. *E* (GPa)	Pred. *E* (GPa)
				107[69]		
				108[70]		
				92[42]		
BaO	63	38[63]	111	66[63]	160	96[63]
				69[71]		
				75[42]		
ZnO	157	46[72]	275	160[73]	395	112[74]
				183[73]		
				168[75]		
c-ZnS	37	33[76]	65	78[76]	94	86[76]
		32[77]		78[77]		
				77[78]		
				73[37]		
h-ZnS	52	33[79]	91	82[80]	131	86[79]
				81[81]		
				77[78]		
				83[82]		
ZnSe	31	29[83]	55	60[83]	79	76[83]

Materials	Pred. *G* (GPa)	Meas. *G* (GPa)	Pred. *B* (GPa)	Meas. *B* (GPa)	Meas. *E* (GPa)	Pred. *E* (GPa)
				61[84]		
				57[85]		
ZnTe	21	23[86]	36	50[86]	52	61[86]
				52[87]		60[87]
				51[53]		
				44[88]		
CdO	104	45[89]	183	125[89]	263	121[89]
		46[89]		164[90]		204[89]
				108[91]		
				145[42]		
CdS	33	34[92]	57	72[92]	82	88[92]
				61[93]		
				74[94]		
CdSe	24	15[95]	41	45[95]	59	41[95]
				54[96]		
CdTe	17	19[97]	30	49[97]	44	50[97]
		15[98]		45[98]		41[98]
				33[99]		

Materials	Pred. *G* (GPa)	Meas. *G* (GPa)	Pred. *B* (GPa)	Meas. *B* (GPa)	Meas. *E* (GPa)	Pred. *E* (GPa)
				42[42]		
HgS	20	21[100]	36	57[100]	51	58[100]
				19[101]		
				55[102]		
				23[103]		
HgSe	43	15[87]	76	52[87]	109	41[87]
				50[43]		
				58[102]		
HgTe	17	15[87]	30	44[87]	43	41[87]
				40[104]		
				42[43]		
				43[43]		
Li_2O	75	68[105]	131	79[105]	189	158[105]
Al_2O_3	557	129[106]	978	231[106]	1405	328[106]
Fe_2O_3	543	113[107]	965	203[107]	1370	287[107]
Sc_2O_3	283	79[108]	497	168[108]	714	205[108]
Y_2O_3	228	62[109]	401	138[109]	576	162[109]
Er_2O_3	231	67[110]	405	148[110]	581	174[110]

Materials	Pred. *G* (GPa)	Meas. *G* (GPa)	Pred. *B* (GPa)	Meas. *B* (GPa)	Meas. *E* (GPa)	Pred. *E* (GPa)
Yb_2O_3	255	181[111]	-	-	-	-
Lu_2O_3	233	114[112]	-	-	-	-
MnO	151	66[113]	266	155[113]	381	174[113]
		69[114]		151[114]		179[114]
				162[115]		
				142[116]		
CoO	167	69[63]	294	181[63]	421	185[63]
		71[76]		185[76]		190[76]
				191[91]		
				178[65]		
NiO	170	91[63]	298	173[63]	428	231[63]
				236[117]		
				214[118]		
MnTe	43	29[119]	75	47[119]	108	72[119]
SnS	21	31[120]	38	53[120]	54	78[120]
SnTe	28	28[114]	49	42[76]	70	57[76]
		22[76]				
PbS	22	30[121]	38	63[121]	54	78[121]

Materials	Pred. *G* (GPa)	Meas. *G* (GPa)	Pred. *B* (GPa)	Meas. *B* (GPa)	Meas. *E* (GPa)	Pred. *E* (GPa)
				52[122]		
				52[123]		
				60[116]		
PbSe	18	49[124]	31	59[124]	45	37[125]
				45[123]		
				54[116]		
				48[122]		
PbTe	20	23[76]	36	41[76]	52	58[76]
		41[124]		38[124]		28[125]
				41[126]		
				46[116]		
GeS	33	17[127]	58	21[127]	84	40[127]
GeSe	24	14[128]	41	21[128]	59	34[128]
GeTe	42	49[129]	74	55[130]	107	
CuCl	17	9[131]	30	42[131]	44	26[131]
				38[132]		
				40[133]		
				48[134]		

Materials	Pred. *G* (GPa)	Meas. *G* (GPa)	Pred. *B* (GPa)	Meas. *B* (GPa)	Meas. *E* (GPa)	Pred. *E* (GPa)
CuBr	14	9[135]	24	40[135]	34	25[135]
				37[136]		
				37[137]		
				44[134]		
CuI	10	15[138]	17	36[138]	25	40[138]
				36[139]		
				34[137]		
AgCl	17	8[140]	29	43[140]	42	23[140]
				43[141]		
				44[142]		
				44[143]		
AgBr	13	8[76]	24	40[76]	34	22[76]
				40[141]		
				39[144]		
				41[142]		
TlCl	9	9[145]	17	24[145]	24	25[145]
				16[146]		
				24[91]		

Materials	Pred. *G* (GPa)	Meas. *G* (GPa)	Pred. *B* (GPa)	Meas. *B* (GPa)	Meas. *E* (GPa)	Pred. *E* (GPa)
				23[141]		
TlBr	8	9[76]	14	22[76]	20	24[76]
La_3Te_4	74	25[147]	130	50[147]	187	64[147]
Sm_3Se_4	122	32[148]	215	40[148]	309	76[148]
Mg_2Si	37	46[149]	64	54[149]	92	108[149]
Mg_2Ge	37	43[150]	66	49[150]	94	113[150]
Mg_2Sn	28	44[150]	50	41[150]	71	80[150]
Mg_2Pb	48	31[151]	84	34[151]	121	52[151]
Na_2S	16	21[152]	28	20[152]	40	48[152]
ZnSb	23	29[153]	40	48[153]	58	73[153]
CdSb	49	18[154]	86	39[154]	124	47[154]
Sb	116		204	162[14]	293	
Li	6.9	4[76]	12	12[76]	17	11[76]
		4.4[155]		14[156]		
				15[157]		
				14[158]		
Na	3.9	2.1[76]	6.8	7.9[76]	9.8	5.6[76]
		4.2[158]		8.7[157]		

Materials	Pred. *G* (GPa)	Meas. *G* (GPa)	Pred. *B* (GPa)	Meas. *B* (GPa)	Meas. *E* (GPa)	Pred. *E* (GPa)
				7.2[159]		
				8[160]		
K	1.8	0.9[161]	3.1	4.2[161]	4.4	2.5[161]
		3.9[162]		4.7[157]		
				2.6[159]		
				3.7[163]		
Rb	0.7	0.8[76]	1.2	4.2[164]	1.8	2.2[76]
		0.3[164]		4.9[157]		
				5.8[165]		
Be	141	148[76]	249	110[76]	357	311[76]
		156[166]		103[14]		
				114[77]		
				125[160]		
Mg	21	17[167]	36	35[77]	52	45[167]
		17[77]		39[160]		50[168]
				35[169]		
				39[170]		
Ca	4.7	8[171]	8.2	16[172]	12	21[171]

Materials	Pred. *G* (GPa)	Meas. *G* (GPa)	Pred. *B* (GPa)	Meas. *B* (GPa)	Meas. *E* (GPa)	Pred. *E* (GPa)
		2.9[164]		18[14]		
				16[173]		
				17[174]		
Al	35	26[76]	62	63[76]	89	70[76]
		26[175]		73[176]		
				59[160]		
				61[177]		
Ga	21		36	49[178]	52	
				70[14]		
				67[179]		
				40[173]		
In	5.9	5.5[77]	10.4	30[173]	15	14[76]
		6.7[77]		42[180]		
				37[181]		
				46[77]		
Tl	12	7.3[77]	21	37[160]	30	15[182]
		5.4[77]		38[77]		
				36[14]		

Materials	Pred. *G* (GPa)	Meas. *G* (GPa)	Pred. *B* (GPa)	Meas. *B* (GPa)	Meas. *E* (GPa)	Pred. *E* (GPa)
Sn	27	18[76]	48	42[76]	68	49[76]
				58[183]		
				51[14]		
				54[184]		
Pb	9.1	8.5[76]	16	14[76]	23	24[76]
Cu	26	47[76]	45	110[76]	65	127[76]
				79[185]		
Ag	24	29[76]	43	76[76]	62	80[76]
		28[186]		56[187]		81[188]
Au	28	28[76]	49	77[76]	71	78[76]
Zn	57	40[76]	101	92[76]	144	99[76]
		45[77]		90[173]		
				72[77]		
				71[170]		
Cd	26	24[76]	45	55[76]	65	63[76]
		21[77]		40[160]		
				46[181]		
				50[77]		

Materials	Pred. *G* (GPa)	Meas. *G* (GPa)	Pred. *B* (GPa)	Meas. *B* (GPa)	Meas. *E* (GPa)	Pred. *E* (GPa)
Ti	39	43[76]	68	86[76]	97	115[76]
		39[189]		94[190]		127[191]
Zr	54	35[76]	96	95[76]	137	147[76]
		36[189]		89[192]		96[189]
				95[77]		
				90[173]		
Hf	155	58[76]	272	143[76]	390	95[76]
		61[77]		119[193]		
V	106	76[164]	187	186[156]	268	130[76]
				180[173]		
				170[194]		
				157[195]		
Nb	80	38[76]	141	150[196]	202	105[76]
		36[197]		168[198]		
				169[199]		
				170[200]		
Ta	119	69[76]	208	194[76]	299	185[76]
				206[201]		181[202]

Materials	Pred. *G* (GPa)	Meas. *G* (GPa)	Pred. *B* (GPa)	Meas. *B* (GPa)	Meas. *E* (GPa)	Pred. *E* (GPa)
				211[14]		
				209[201]		
Cr	160	115[76]	281	306[76]	403	278[76]
				280[173]		
Mo	117	127[203]	206	251[156]	295	322[76]
				260[199]		
				255[14]		
				262[200]		
W	165	160[76]	290	279[76]	417	410[76]
		160[203]		275[69]		410[203]
				308[204]		
				307[205]		
Mn	64	76[76]	113	158[76]	163	159[76]
				130[206]		
Fe	121	88[207]	222	217[173]	304	212[207]
		83[208]		210[209]		
				198[210]		
				230[196]		

Materials	Pred. *G* (GPa)	Meas. *G* (GPa)	Pred. *B* (GPa)	Meas. *B* (GPa)	Meas. *E* (GPa)	Pred. *E* (GPa)
Co	143	84[77]	251	205[211]	360	213[76]
				220[209]		
				226[212]		
				268[212]		
Ni	141	83[76]	248	207[76]	336	218[76]
				210[209]		
				227[173]		
				250[196]		
Pt	58	64[76]	102	156[76]	147	177[76]
		80[118]				
LiF	56	49[213]	98	69[213]	140	118[213]
				67[214]		
				80[215]		
				77[216]		
NaF	32	37[76]	56	51[76]	80	89[76]
				44[217]		
				49[67]		
				47[214]		

Materials	Pred. *G* (GPa)	Meas. *G* (GPa)	Pred. *B* (GPa)	Meas. *B* (GPa)	Meas. *E* (GPa)	Pred. *E* (GPa)
KF	18	16[76]	31	32[76]	45	42[76]
				29[116]		
				30[218]		
				31[219]		
RbF	14	15[220]	25	31[220]	36	38[220]
				26[221]		
				27[218]		
				25[116]		
LiCl	21	19[76]	36	32[76]	52	48[76]
				34[215]		
				28[67]		
				34[217]		
NaCl	14	13[222]	24	24[222]	35	34[222]
				24[223]		
				24[224]		
				23[225]		
KCl	8.9	9.2[226]	16	16[226]	23	24[226]
				17[227]		

Materials	Pred. *G* (GPa)	Meas. *G* (GPa)	Pred. *B* (GPa)	Meas. *B* (GPa)	Meas. *E* (GPa)	Pred. *E* (GPa)
				20[228]		
				18[229]		
RbCl	7.6	7.6[76]	13	17[76]	19	20[230]
				17[217]		
				16[214]		
				15[231]		
CsCl	6.1	10[76]	11	18[76]	15	25[76]
				17[218]		
				17[232]		
				16[141,233]		
LiBr	16	15[76]	28	25[76]	40	37[76]
				27[234]		
				24[218]		
				26[215]		
NaBr	11	12[76]	19	19[76]	28	29[76]
				25[216]		
				21[235]		
				19[217]		

Materials	Pred. *G* (GPa)	Meas. *G* (GPa)	Pred. *B* (GPa)	Meas. *B* (GPa)	Meas. *E* (GPa)	Pred. *E* (GPa)
KBr	7.2	7.9[213]	13	15[213]	18	20[213]
				12[236]		
				20[216]		
				15[218]		
RbBr	6.1	6.5[76]	11	19[76]	15	17[237]
				18[216]		
				14[219]		
				15[238]		
CsBr	5	8.8[239]	8.7	17[239]	13	22[239]
LiI	11	11[76]	19	18[76]	28	27[76]
				19[215]		
				22[219]		
				20[116]		
NaI	7.9	8.5[76]	14	16[76]	20	22[76]
				13[240]		
				14[221]		
				15[219]		
KI	5.4	6[76]	9	14[76]	14	15[76]

Materials	Pred. *G* (GPa)	Meas. *G* (GPa)	Pred. *B* (GPa)	Meas. *B* (GPa)	Meas. *E* (GPa)	Pred. *E* (GPa)
				13[215]		
				11[221]		
				8.7[236]		
RbI	4.6	5[76]	8	14[215]	12	13[241]
				11[221]		
				15[236]		
				12[217]		
$AgSbTe_2$	30	19[242]	52	53[242]	75	51[242]
$AgBiSe_2$	23	21[243]	41	53[243]	58	55[243]
$AgBiTe_2$	23	16[244]	41	47[244]	58	43[244]
$CuGaTe_2$	21	25[245]	40	46[245]	58	63[245]
				50[246]		
$CuInSe_2$	35	22[247]	63	50[247]	90	58[247]
		26[248]		59[246]		65[248]
				54[249]		
$CuInTe_2$	21	19[248]	37	45[248]	53	51[248]
		21[248]		43[246]		54[248]
$AgGaTe_2$	19	17[250]	35	40[250]	50	45[250]

Materials	Pred. *G* (GPa)	Meas. *G* (GPa)	Pred. *B* (GPa)	Meas. *B* (GPa)	Meas. *E* (GPa)	Pred. *E* (GPa)
				42[246]		
				43[251]		
$AgInTe_2$	17		30	30[249]	43	
				37[251]		
				36[246]		

Table S2. The predicted acoustical properties based on the model proposed in this work, along with a comparison to measurements. v_T is the transverse sound velocity, v_L is the longitudinal sound velocity, v_S is the harmonic mean sound velocity and Z is the acoustic impendence. Acoustic impendence is estimated from the product of mass density and longitudinal sound velocity.

**Materials**	**Pred. v_T (m s^{-1})**	**Meas. v_T (m s^{-1})**	**Pred. v_L (m s^{-1})**	**Meas. v_L (m s^{-1})**	**Pred. v (m s^{-1})**	**Meas. v (m s^{-1})**	**Pred. Z (10^{-6} kg m^{-2} s^{-1})**	**Meas. Z (10^{-6} kg m^{-2} s^{-1})**
Cumulene	18574	-	32033	-	20612	21663[252]	113	-
Graphene	14349	14700[253]	24746	22000[253]	15924	16065[253]	87	77[253]
Diamond	11277	12245[254]	19448	18330[254]	12515	13382[254]	68	64[254]
Si	5658	5825[255]	9713	9117[255]	6276	6401[255]	23[255]	21
Ge	3615	3542[256]	6226	4913[256]	4012	3829[256]	33	26[256]
3C-SiC	7454	7690[257]	12853	12210[257]	8272	8464[257]	41	39[257]
c-BN	9276	10410[31]	15998	15850[31]	10294	11401[31]	56	55[31]
h-BN	11196	7120[258]	19310	14860[258]	12425	8006[258]	42	32[258]
BP	6163	6850[259]	10624	10670[259]	6839	7523[259]	32	32[259]
BAs	4200	4947[260]	7240	7434[260]	4661	4635[260]	38	39[260]
BSb	3314	3571[260]	5708	5563[260]	3677	3921[260]	37	36[260]
AlN	6457	6333[261]	11136	10127[261]	7166	6975[261]	36	33[261]
AlAs	3480	3900[262]	5982	5700[262]	3861	4248[262]	22	21[262]
AlSb	2839	3087[263]	4870	4528[263]	3149	3364[263]	21	19[263]
GaN	4322	4702[264]	7454	7744[264]	4796	5195[264]	45	47[264]
GaP	3613	4131[265]	6223	5847[265]	4010	4479[265]	26	24[265]
GaAs	3021	3345[266]	5204	4731[266]	3353	3626[266]	28	25[266]

Materials	Pred. v_T (m s^{-1})	Meas. v_T (m s^{-1})	Pred. v_L (m s^{-1})	Meas. v_L (m s^{-1})	Pred. v (m s^{-1})	Meas. v (m s^{-1})	Pred. Z (10^{-6} kg m^{-2} s^{-1})	Meas. Z (10^{-6} kg m^{-2} s^{-1})
GaSb	2602	2460[267]	4477	4240[267]	2887	2729[267]	25	24[267]
InN	3740	2518[268]	6450	4810[268]	4150	2817[268]	44	33[268]
InP	3207	2631[269]	5523	5130[269]	3558	3429[269]	26	25[269]
InAs	3369	2238[270]	5806	4282[270]	3739	2503[270]	33	24[270]
InSb	2392	1955[271]	4117	3766[271]	2654	2188[271]	24	22[271]
BeO	7694	4667[61]	13269	8242[61]	8539	5190[61]	40	25[61]
MgO	6721	6596[272]	11591	9112[272]	7459	7125[272]	41	33[272]
CaO	5446	4907[273]	9392	8156[273]	6044	5426[273]	31	27[273]
SrO	3887	3429[273]	6703	6077[273]	4313	3814[273]	34	30[273]
BaO	3136	2435[273]	5409	4712[273]	3480	2726[273]	36	31[273]
ZnO	5314	2793[274]	9166	6024[274]	5898	3145	52	34
c-ZnS	3051	2771[275]	5260	5024[276]	3386	3087[275]	22	21[275]
h-ZnS	3605	2656[277]	6216	5494[277]	4000	2985[277]	25	22[277]
ZnSe	2463	2300[278]	4246	4598[278]	2733	2580[278]	22	24[278]
ZnTe	1933	2740[279]	3322	3580[279]	2145	2932[279]	19	20[279]
CdO	3596	-	6202	-	3991	2008[280]	51	-
CdS	2641	1760[281]	4554	4420[281]	2931	1994[281]	22	21[281]
CdSe	2062	1495[282]	3555	3583[282]	2289	1691[282]	20	20[282]

Materials	Pred. v_T (m s^{-1})	Meas. v_T (m s^{-1})	Pred. v_L (m s^{-1})	Meas. v_L (m s^{-1})	Pred. v (m s^{-1})	Meas. v (m s^{-1})	Pred. Z (10^{-6} kg m^{-2} s^{-1})	Meas. Z (10^{-6} kg m^{-2} s^{-1})
CdTe	1740	1460[279]	2991	3470[279]	1930	1651[279]	18	20[279]
HgS	1638	1848[283]	2823	3243[283]	1818	2053[283]	22	25[283]
HgSe	2270	1673[284]	3914	2769[284]	2519	1849[284]	34	24[284]
HgTe	1464	1603[285]	2522	2555[285]	1625	1765[285]	20	21[285]
Li_2O	6156	5400[286]	10617	10017[286]	6832	6028[286]	21	20[286]
Al_2O_3	11964	6480[287]	20633	11050[287]	13277	7183	82	44
Cr_2O_3	10375	4550[288]	17892	8000[288]	11513	5057[288]	94	42[288]
α-Fe_2O_3	10289	2800[289]	17745	5063[289]	11419	4400[289]	93	27[289]
β-Ga_2O_3	6062	3400[290]	10454	5400[290]	6727	3742[290]	62	32[290]
Sc_2O_3	8691	4890[291]	14989	8470[291]	9645	5428[291]	58	33[291]
Y_2O_3	6819	3970[292]	11760	6945[292]	7567[292]	4411	59	35[292]
Tm_2O_3	5203	2794[291]	8973	5151[291]	5774	3117[291]	80	46[291]
Er_2O_3	5223	2798[291]	9008	5167[291]	5796	3122[291]	78	45[291]
Yb_2O_3	5360	2831[291]	9244	5154[291]	5949	3155[291]	84	47[291]
Lu_2O_3	5119	2820[291]	8828	5052[291]	5681	3139[291]	80	46[291]
MnO	5376	3522[293]	9272	6740[293]	5967	3940[293]	50	36[293]
CoO	5163	3555[293]	8903	6323[293]	5729	3955[293]	57	41[293]
NiO	5057	3503[293]	8721	6799[293]	5612	3922[293]	59	46[293]

Materials	Pred. v_T (m s^{-1})	Meas. v_T (m s^{-1})	Pred. v_L (m s^{-1})	Meas. v_L (m s^{-1})	Pred. v (m s^{-1})	Meas. v (m s^{-1})	Pred. Z (10^{-6} kg m^{-2} s^{-1})	Meas. Z (10^{-6} kg m^{-2} s^{-1})
MnTe	2694	1945[294]	4646	3786[294]	2990	2178[294]	28	23[294]
SnS	2057	1940[295]	3547	3368[295]	2283	2154[295]	18	18[295]
SnO_2	4112	3838[296]	7092	6108[296]	4564	4225[296]	50	43[296]
$SnSe_2$	2184	1340[297]	3766	2330[297]	2424	1488[297]	22	14[297]
$SnSe_2$	2184	2303[297]	3766	4210[297]	2424	2568[297]	22	25[297]
SnSe (b)	1796	2197[298]	3097	3973[298]	1993	2447[298]	19	24[298]
SnSe (a)	1796	1838[298]	3097	3564[298]	1993	2057[298]	19	22[298]
SnTe	2095	1850[299]	3612	3400[299]	2325	2063[299]	23	22[299]
PbS	1705	1910[300]	2935	3460[300]	1891	2128[300]	22	26[300]
PbSe	1490	1600[301]	2565	3150[301]	1653	1793[301]	21	26[301]
PbTe	1596	1600[300]	2748	2900[300]	1771	1782[300]	23	24[300]
GeS	2833	2070[302]	4886	3410[302]	3144	2287[302]	21	14[302]
GeSe	2095	1952[303]	3612	3210[303]	2325	2156	20	18
GeTe	2651	2181[304]	4572	3620[304]	2942	2411[304]	28	22[304]
GaS	5263	2980[305]	9077	4700[305]	5841	3277[305]	36	18[305]
GaS	3250	1800[305]	5604	2900[305]	3606	1984[305]	22	11[305]
GaSe	4505	2641[306]	7769	4515[306]	4999	2928[306]	40	23[306]
GaSe	2513	1185[306]	4333	2482[306]	2789	1332[306]	22	13[306]

Materials	Pred. v_T (m s^{-1})	Meas. v_T (m s^{-1})	Pred. v_L (m s^{-1})	Meas. v_L (m s^{-1})	Pred. v (m s^{-1})	Meas. v (m s^{-1})	Pred. Z (10^{-6} kg m^{-2} s^{-1})	Meas. Z (10^{-6} kg m^{-2} s^{-1})
GaTe	3234	-	5575	-	3589	3400[307]	30	-
InSe	4895	2077[308]	8442	3640[308]	5432	2308[308]	47	20[308]
InSe	2223	1451[308]	3830	2526[308]	2466	1611[308]	21	14[308]
InTe	2217	1435[309]	3822	2177[309]	2460	1570[309]	24	14[309]
TlSe	1979	1605[310]	3407	2621[310]	2196	1771[310]	28	22[310]
γ-CuCl	2072	1872[311]	3574	3370[311]	2300	2084[311]	15	14[311]
γ-CuBr	1642	1716[312]	2833	3115[312]	1823	1912[312]	15	16[312]
γ-CuI	1335	1792[312]	2302	2821[312]	1481	1970[312]	13	16[312]
AgCl	1703	1449[313]	2937	3117[313]	1890	1631[313]	17	18[313]
AgBr	1460	1247[314]	2518	2807[314]	1620	1407[314]	16	18[314]
γ-AgI	1182	1133[315]	2037	2276[315]	1311	1271[315]	12	13[315]
HgI_2	1227	1070[316]	2202	2230[316]	1417	1203[316]	14	14[316]
HgI_2	1227	740[316]	2202	1550[316]	1417	832[316]	14	10[316]
PbI_2	1046	930[317]	1802	1540[317]	1160	1028[317]	11	9[317]
TlCl	1235	1057[318]	2130	2392[318]	1371	1351[318]	13	15[318]
TlBr	1110	1008[319]	1914	2246[319]	1232	1137[319]	13	15[319]
β-PbF_2	1980	1636[320]	3414	3309[320]	2197	1836[320]	27	26[320]
SbI_3	1407	984[321]	2427	1758[321]	1562	1095[321]	13	10[321]

Materials	Pred. v_T (m s^{-1})	Meas. v_T (m s^{-1})	Pred. v_L (m s^{-1})	Meas. v_L (m s^{-1})	Pred. v (m s^{-1})	Meas. v (m s^{-1})	Pred. Z (10^{-6} kg m^{-2} s^{-1})	Meas. Z (10^{-6} kg m^{-2} s^{-1})
BiI_3	714	1114[321]	1230	2267[321]	792	1251[321]	7	13[321]
Sb_2S_3	3911	-	6746	-	4341	2160[322]	46	
Sb_2Se_3	2993	-	5162	-	3321	2421[323]	45	
Sb_2Te_3	3028	1970[324]	5221	2960[324]	3360	2154[324]	68	57[324]
Bi_2Te_3	1805	1700[325]	3105	3300[325]	2002	1903[325]	27	29[325]
La_3Te_4	3317	2010[326]	5662	3580[326]	3678	2236[326]	39	25[326]
Sm_3Se_4	4131	1895[327]	7115	3603[327]	4584	2119[327]	52	26[327]
Mg_2Si	4336	4900[328]	7266	7665[328]	4799	5384[328]	15	15[328]
Mg_2Ge	3521	3800[329]	5970	6250[329]	3902	4198[329]	19	19[329]
Mg_2Sn	2853	3066[330]	4810	4900[330]	3160	3377[330]	17	18[330]
Mg_2Pb	3058	2361[331]	5178	3597[331]	3388	2586[331]	27	19[331]
$CoSb_3$	2527	2643[332]	4357	4590[332]	2804	2934[332]	4.2	4.4[332]
Na_2S	2976	3369[333]	5120	6616[333]	3302	3775[333]	9	12[333]
Na_2Se	2296	2456[333]	3949	4413[333]	2548	2845[333]	10	12[333]
Na_2Te	1855	2244[333]	3179	3822[333]	2058	2488[333]	9	11[333]
Na_2Po	1469	1470[334]	2511	2701[334]	1629	1640[334]	10	11[334]
Cu_2O	2384	1303[335]	4111	4541[335]	2645	1485[335]	25	28[335]
Cu_2S	3078	1773[336]	5308	3634[336]	3416	1991[336]	2.5	1.7[336]

Materials	Pred. v_T (m s^{-1})	Meas. v_T (m s^{-1})	Pred. v_L (m s^{-1})	Meas. v_L (m s^{-1})	Pred. v (m s^{-1})	Meas. v (m s^{-1})	Pred. Z (10^{-6} kg m^{-2} s^{-1})	Meas. Z (10^{-6} kg m^{-2} s^{-1})
Cu_2Se	2600	2116[337]	4484	5040[338,339]	2885	2393[338,339]	32	36[338,339]
α-Ag_2S	1347	-	2378	-	1531	1610[340]	17	
α-Ag_2Se	2042	-	3522	-	2266	1453[341]	28	
α-Ag_2Te	1347	1200[342]	2324	3900[342]	1495	1367[342]	20	33[342]
α-Zn_3P_2	3905	2502[343]	6713	5181[343]	4333	2812[343]	31	24[343]
α-Zn_3As_2	3267	1980[344]	5614	4080[344]	3625	2224[344]	31	23[344]
Cd_3P_2	2693	2002[343]	4621	4150[343]	2987	2250[343]	26	23[343]
α-Cd_3As_2	2499	1600[343]	4293	3552[343]	2773	1804[343]	27	23[343]
α-ZnP_2	4829	3375[345]	8327	5649[345]	5359	3735[345]	29	20[345]
$ZnAs_2$	2919	2532[345]	5032	4106[345]	3239	2793[345]	25	21[345]
CdP_2	3930	2602[345]	6776	4629[345]	4361	2895[345]	28	19[345]
2H-CdI_2	1211	1199[346]	2087	2563[346]	1344	1349[346]	12	15[346]
ZnSb		-		-	2136	2241[347]		
CdSb	16	-		-	3008	1845[348]		
As (binary)	5241	2990[349]	9038	4790[349]	5816	3294[349]	53	28[349]
Sb	4256	2219[350]	7339	3891[350]	4723	2466[350]	48	26[350]
Se	2526	1953[351]	4379	4173[351]	2805	2461[351]	21	20[351]
Te	1627	1410[352]	2822	2287[352]	1807	2468[352]	18	15[352]

Materials	Pred. v_T ($m\ s^{-1}$)	Meas. v_T ($m\ s^{-1}$)	Pred. v_L ($m\ s^{-1}$)	Meas. v_L ($m\ s^{-1}$)	Pred. v ($m\ s^{-1}$)	Meas. v ($m\ s^{-1}$)	Pred. Z ($10^{-6}\ kg\ m^{-2}\ s^{-1}$)	Meas. Z ($10^{-6}\ kg\ m^{-2}\ s^{-1}$)
MgB_2	7655	5525[353]	13173	9846[353]	8494	6149[353]	34	26[353]
TiB_2	7167	7592[353]	12356	12097[353]	7954	8360[353]	75	74[353]
ZrB_2	9003	6079[354]	15514	9470[354]	9991	6676[354]	69	43[354]
HfB_2	4900	4598[354]	8430	6994[354]	5437	5035[354]	94	78[354]
CaB_6	7923	6709[355]	13663	10247[355]	8792	7350[355]	33	25[355]
SrB_6	5621	5337[356]	9662	9969[356]	6236	5960[356]	33	34[356]
BaB_6	4792	5440[356]	8227	8940[356]	5315	6009[356]	36	39[356]
LaB_6	6240	6221[357]	10762	10886[357]	6925	6912[357]	51	51[357]
CeB_6	6152	5594[358]	10610	8384[358]	6828	6114[358]	51	40[358]
PrB_6	6126	4928[359]	10566	8288[359]	6799	5456[359]	51[c]	40[359]
NdB_6	6071	4764[359]	10471	8110[359]	6738	5280[359]	52	40[359]
PmB_6	6137	4620[359]	10585	7974[359]	6811	5127[359]	52	40[359]
SmB_6	6002	4574[360]	10352	8294[360]	6661	5097[360]	53	42[360]
EuB_6	5485	4391[361]	9459	8442[361]	6087	4913[361]	46	41[361]
GdB_6	5920	4170[359]	10210	7476[359]	6570	4642[359]	54	40[359]
TbB_6	5890	4032[359]	10158	7350[359]	6537	4495[359]	55	40[359]
DyB_6	5850	3890[359]	10089	7203[359]	6492	4341[359]	55	40[359]
HoB_6	5828	3756[359]	10052	7077[359]	6468	4197[359]	56	39[359]

Materials	Pred. v_T (m s^{-1})	Meas. v_T (m s^{-1})	Pred. v_L (m s^{-1})	Meas. v_L (m s^{-1})	Pred. v (m s^{-1})	Meas. v (m s^{-1})	Pred. Z (10^{-6} kg m^{-2} s^{-1})	Meas. Z (10^{-6} kg m^{-2} s^{-1})
ErB_6	5808	3613[359]	10017	6946[359]	6446	4043[359]	56	39[359]
TmB_6	5798	3457[359]	9999	6816[359]	6434	3874[359]	56	38[359]
YbB_6	5376	3405[361]	9271	7233[361]	5966	3832[361]	51	40[361]
LuB_6	5738	3139[359]	9895	6532[359]	6367	3529[359]	56	37[359]
YB_{12}	8022	7524[362]	12011	14337[362]	8768	8414[362]	41	49[362]
ZrB_{12}	8223	6530[362]	14181	15372[362]	9125	7381[362]	51	55[362]
TbB_{12}	6102	5939[362]	10510	12688[362]	6771	6686[362]	48	58[362]
DyB_{12}	5923	5763[362]	10194	12679[362]	6572	6496[362]	47	58[362]
HoB_{12}	5972	5926[362]	10284	12031[362]	6627	6653[362]	48	56[362]
ErB_{12}	6024	5571[362]	10376	10414[362]	6684	6222[362]	49	49[362]
TmB_{12}	6012	5864[362]	10357	11640[362]	6672	6575[362]	49	55[362]
YbB_{12}	5182	5689[362]	8781	11842[362]	5742	6396[362]	42	57[362]
LuB_{12}	6105	5996[362]	10522	12818[362]	6775	6750[362]	51	62[362]
B_4C	12171	8283[357]	19221	14496[357]	13388	9203[362]	39	29[362]
Li	3706	2820[363]	5535	6030[363]	4049	3175[363]	3.0	3.2[363]
Na	2053	1620[363]	3066	3310[363]	2243	1820[363]	3.0	3.2[363]
K	1462	1230[363]	2184	2600[363]	1597	1384[363]	1.9	2.3[363]
Rb	697	770[363]	1041	1430[363]	761	860[363]	1.6	2.2[363]

Materials	Pred. v_T (m s^{-1})	Meas. v_T (m s^{-1})	Pred. v_L (m s^{-1})	Meas. v_L (m s^{-1})	Pred. v (m s^{-1})	Meas. v (m s^{-1})	Pred. Z (10^{-6} kg m^{-2} s^{-1})	Meas. Z (10^{-6} kg m^{-2} s^{-1})
Be	9012	8330[363]	13468	12700[363]	9847	9125[363]	25	23[363]
Mg	2788	3170[363]	4164	5700[363]	3046	3530[363]	12	16[363]
Ca	1798	2210[363]	2685	4180[363]	1964	2470[363]	4.1	6.4[363]
Al	3717	3150[363]	5555	6360[363]	4062	3536[363]	15	17[363]
Ga	1910	750[363]	2908	3030[363]	2092	856[363]	17	18[363]
In	1261	710[363]	1885	2460[363]	1378	810[363]	7.4	9.7[363]
Tl	1033	480[363]	1549	1630[363]	1130	547[363]	18	19[363]
Sn	1954	1650[363]	3277	3300[363]	2163	1851[363]	24	24[363]
Pb	917	710[363]	1395	2050[363]	1005	807[363]	16	23[363]
Cu	1742	2560[363]	2605	4900[363]	1904	2864[363]	23	44[363]
Ag	1566	1690[363]	2344	3640[363]	1712	1903[363]	25	38[363]
Au	1221	1190[363]	2050	3280[363]	1352	1352[363]	40	63[363]
Zn	2911	2290[363]	4352	3890[363]	3181	2538[363]	31	28[363]
Cd	1769	1690[363]	2644	2980[363]	1933	1879[363]	23	26[363]
Ti	2999	2920[363]	4481	6260[363]	3277	3288[363]	20	28[363]
Zr	2865	1950[363]	4281	4360[363]	3130	2200[363]	30	31[363]
Hf	3511	2000[363]	5246	3670[363]	3836	2231[363]	70	49[363]
V	4281	2780[363]	6472	6000[363]	4685	3131[363]	40	37[363]

Materials	Pred. v_T (m s^{-1})	Meas. v_T (m s^{-1})	Pred. v_L (m s^{-1})	Meas. v_L (m s^{-1})	Pred. v (m s^{-1})	Meas. v (m s^{-1})	Pred. Z (10^{-6} kg m^{-2} s^{-1})	Meas. Z (10^{-6} kg m^{-2} s^{-1})
Nb	3142	2090[363]	4741	5100[363]	3437	2366[363]	40	44[363]
Ta	2729	2030[363]	4108	4240[363]	2985	2283[363]	69	71[363]
Cr	4830	3980[363]	7440	6850[363]	5297	4416[363]	54	49[363]
Mo	3437	3510[363]	5664	6650[363]	3798	3924[363]	58	68[363]
W	2980	2840[363]	4890	5320[363]	3291	3173[363]	94	102[363]
Mn	3047	3280[363]	4552	5560[363]	3329	3634[363]	33	41[363]
Fe	3978	3220[363]	6407	5950[363]	4386	3593[363]	51	47[363]
Co	4143	3000[363]	6317	5730[363]	4539	3356[363]	55	50[363]
Ni	4071	3080[363]	6243	5810[363]	4463	3442[363]	56	52[363]
Pt	1671	1690[363]	2793	4080[363]	1849	1912[363]	60	88[363]
Cs	983	590[363]	1469	1090[363]	1074	659[363]	0.9	0.7[363]
Sr	1319	1520[363]	1970	2780[363]	1441	1694[363]	2.5	3.6[363]
Ba	1553	1160[363]	2320	2080[363]	1697	1292[363]	8.4	7.5[363]
LiF	4645	4305[364]	8010	7149[364]	5155	4761[364]	21	19[364]
NaF	3397	3323[364]	5859	5663[364]	3770	3683[364]	16	16[364]
KF	2964	2548[364]	4647	4630[364]	2990	2840[364]	12	12[364]
RbF	1956	2133[364]	3372	3945[364]	2170	2380[364]	13	15[364]
LiCl	3198	3058[364]	5516	5260[364]	3549	3393[364]	11	11[364]

Materials	Pred. v_T (m s^{-1})	Meas. v_T (m s^{-1})	Pred. v_L (m s^{-1})	Meas. v_L (m s^{-1})	Pred. v (m s^{-1})	Meas. v (m s^{-1})	Pred. Z (10^{-6} kg m^{-2} s^{-1})	Meas. Z (10^{-6} kg m^{-2} s^{-1})
NaCl	2568	2591[364]	4429	4528[364]	2850	2879[364]	9.6	9.8[364]
KCl	2140	2150[364]	3689	3870[364]	2374	2394[364]	7.4	7.7[364]
RbCl	1657	1657[364]	2856	3093[364]	1840	1850[364]	8.1	8.7[364]
CsCl	1357	1585[364]	2340	2839[364]	1506	1764[364]	7.9	9.6[364]
LiBr	2162	2072[364]	3729	3622[364]	2399	2302[364]	13	13[364]
NaBr	1872	1912[364]	3229	3330[364]	2078	2123[364]	10	11[364]
KBr	1638	1685[364]	2824	3032[364]	1818	1876[364]	7.8	8.4[364]
RbBr	1361	1382[364]	2346	2587[364]	1510	1543[364]	7.9	8.7[364]
CsBr	1171	1393[364]	2019	2464[364]	1300	1549[364]	7.5	9.1[364]
LiI	1671	1609[364]	2882	2844[364]	1854	1789[364]	12	12[364]
NaI	1490	1518[364]	2569	2731[364]	1653	1690[364]	9.4	10[364]
KI	1325	1371[364]	2283	2496[364]	1470	1528[364]	7.2	7.9[364]
RbI	1150	1179[364]	1982	2231[364]	1276	1318[364]	7.1	8[364]
CsI	1016	1267[364]	1751	2212[364]	1128	1407[364]	6.8	8.6[364]
$AgSbSe_2$	2012	1733[8]	3465	3433[8]	2232	1942[8]	23	23[8]
$AgSbTe_2$	2060	1485[8]	3550	2861[8]	2286	1662[8]	25	21[8]
$AgBiSe_2$	1729		2978		1918	1322[8]	23	
$AgBiTe_2$	1713	1697[8]	2947	3223[8]	1900	1897[8]	24	26[8]

Materials	Pred. v_T (m s^{-1})	Meas. v_T (m s^{-1})	Pred. v_L (m s^{-1})	Meas. v_L (m s^{-1})	Pred. v (m s^{-1})	Meas. v (m s^{-1})	Pred. Z (10^{-6} kg m^{-2} s^{-1})	Meas. Z (10^{-6} kg m^{-2} s^{-1})
$NaSbTe_2$	2116	2047[8]	3644	3630[8]	2348	2277[8]	19	19[8]
$NaBiTe_2$	1995	1675[8]	3433	3131[8]	2213	1871[8]	21	20[8]
$CuGaTe_2$	2039	2115[8]	3511	3985[8]	2263	2364[8]	21	24[8]
$CuInSe_2$	2523	2100[8]	4351	3770[8]	2800	2338[8]	25	22[8]
$CuInTe_2$	1889	1790[8]	3250	3420[8]	2094	2002[8]	20	21[8]
$AgGaTe_2$	1835		3158		2036	1894[8]		
$AgInTe_2$	1708		2935		1895	1673[8]		

Table S3. The predicted thermal properties based on the model proposed in this work, along with a comparison to measurements. γ is the Grüneisen parameter, α is the linear thermal expansion coefficient and κ_L is the lattice thermal conductivity.

Materials	Pred. γ	Meas. γ	Pred. α (10^{-6} K)	Meas. α (10^{-6} K)	Pred. $\kappa_{L,sine}$ (W $m^{-1}K^{-1}$)	Meas. κ_L (W $m^{-1}K^{-1}$)
Diamond	0.77	0.75[365]	0.47	1[366]	712	2000[367,368]
Si	0.76	0.77[369]	3.2	2.6[370]	93	166[365,368]
		0.56[371]		2.7[369]		156[372]
		1.1[365]				136[373]
Ge	0.75	0.74[22]	3.5	5.7[369]	60	65[365]
		0.76[371]		5.8[22,370]		60[372,374,375]
		1.1[365]				54[373]
3C-SiC	0.87	0.76[371]	2.1	2.9[370]	180	490[367,368]
						300[376]
c-BN	1.3	1.2[377]	1.7	1.8[378]	149	760[365]
		0.7[365]				
h-BN	0.94	0.7[365]			221	220[258]
BP	1.1	1.1[377]	4.4	3.7[379]	65	350[365,368]
		0.75[365]				
BAs	1.1	0.98[380]	5.8	4.1[381]	38	
BSb	1.5	1.1[260]	9.1		13	

**Materials**	**Pred. γ**	**Meas. γ**	**Pred. α (10^{-6} K)**	**Meas. α (10^{-6} K)**	**Pred. $\kappa_{L,sine}$ (W $m^{-1}K^{-1}$)**	**Meas. κ_L (W $m^{-1}K^{-1}$)**
AlN	1.3	0.7[365]			36	200[5]
AlAs	1.1	1.1[382]	8.1	3.5[370]	16	80[383]
		0.66[365]				89[384]
						98[365]
AlSb	1.1	1.2[385]	8.5	4.2[370]	12	56[365]
		0.6[5]				46[375]
GaN	1.3	0.7[365]			19	130[386]
						210[365]
GaP	1.1	0.75[365]	7.2	5.3[370]	22	77[373]
				5.7[214]		100[5]
				5.8[387]		
GaAs	1.1	0.97[382]	7.3	5.4[370]	18	50[384]
		0.75[365]		5.8[388]		45[365,375]
				6[389]		
				5.7[214]		
GaSb	1	0.75[365]	7.4	7.8[389,390]	13.5	37[375]
				6.1[370]		40[365]

Materials	Pred. γ	Meas. γ	Pred. α (10^{-6} K)	Meas. α (10^{-6} K)	Pred. $\kappa_{L,sine}$ (W $m^{-1}K^{-1}$)	Meas. κ_L (W $m^{-1}K^{-1}$)
				6.3[214]		
InN	1.5	0.97[391]			12	38[391]
		0.7[365]				
InP	0.96	1.19[392]	5.8	4.8[388]	24	67[375]
		0.78[393]		4.6[370]		70[394]
		0.6[365]				93[365]
InAs	0.83	0.57[365]	3.5	4.5[390]	45	30[365]
		0.57[395]		4.7[370]		27[375]
InSb	1.5	0.56[365]	10	5.1[214]	5.4	20[365]
				5.4[389]		16[375]
MgO	1.9	1.4[365]	5.9	4.2[396]	31	60[365]
				5.1[397]		
CaO	1.9	1.6[365]	8.4	13[398]	17	27[365]
SrO	1.9	1.5[365]	11	13[399]	10	12[365]
BaO	1.9	1.5[365]	11.5	12.8[400]	7.1	2.3[365]
ZnO	2	2.2[8]			16	54[401]

Materials	Pred. γ	Meas. γ	Pred. α (10^{-6} K)	Meas. α (10^{-6} K)	Pred. $\kappa_{L,sine}$ (W $m^{-1}K^{-1}$)	Meas. κ_L (W $m^{-1}K^{-1}$)
		0.75[365]				60[365]
BeO	1.6	1.4[8]			43	370[58]
c-ZnS	1.5	0.75[365]	14	7[402]	7	27[365,401]
		1.2[380]		6.7[214]		
				6.4[370]		
h-ZnS	1.8	2.1[8]			5.2	-
ZnSe	1.4	1.1[380]	15	7.2[370]	5.2	19[365]
		0.75[365]		7.6[403]		13[383]
				7.7[404]		
				7.4[402]		
ZnTe	1.5	1.1[380]	19	8.4[214]	2.8	18[365]
		0.97[365]		9[405]		11[383]
				8.4[402]		
				8.2[370]		
CdO	1.9		9.7	13[406]	12	
CdS	1.7	0.75[365]			3.1	16[365]
						20[281]
CdSe	1.6	0.6[365]			1.9	9[407]

**Materials**	**Pred. γ**	**Meas. γ**	**Pred. α (10^{-6} K)**	**Meas. α (10^{-6} K)**	**Pred. $\kappa_{L,sine}$ (W $m^{-1}K^{-1}$)**	**Meas. κ_L (W $m^{-1}K^{-1}$)**
						4.4[383]
CdTe	1.4	1.1[380]	18	4.5[214]	2.6	10[279]
		0.52[365]		5[408]		7.5[365]
				4.9[370]		
HgS	0.99	1.5[8]	15	38[118]	4.9	
HgSe	0.78	1.3[8]	5.1	5.5[370]	24	3[409]
						1.9[383]
HgTe	1.3	1.9[10]	18	4.6[370]	2.3	2.5[383]
		1.2[8]				
Li_2O	1.9	1.7[8]			10	11[410]
Al_2O_3	1.8	1.3[371]			73	35[411]
						30[412]
Cr_2O_3	1.9	1.6[8]			59	15[413]
						13.1[414]
α-Fe_2O_3	1.9				57	11.3[415]
β-Ga_2O_3	1.7	1.2[8]			18	10.9[290]
						13[416]

Materials	Pred. γ	Meas. γ	Pred. α (10^{-6} K)	Meas. α (10^{-6} K)	Pred. $\kappa_{L,sine}$ (W $m^{-1}K^{-1}$)	Meas. κ_L (W $m^{-1}K^{-1}$)
Sc_2O_3	1.7	1.5[8]			15	17[417]
Y_2O_3	1.7	1.5[8]			10	14[417]
Tm_2O_3	1.7	1.72[8]			8	
Er_2O_3	1.7	1.72[8]			7.9	-
Yb_2O_3	1.8	1.7[8]			8.4	-
Lu_2O_3	1.7	1.6[8]			7.8	-
MnO	2	1.9[8]	6.8	11[418]	24	10[419]
CoO	2	1.6[8]	6.8	4.5[420]	24	17.0[421]
						10.5[421]
NiO	1.9	1.9[8]			24	27[421]
						32[421]
MnTe	1.9	1.9[8]			3.2	1.8[422]
						1.7[294]
						1.5[383]
SnS	1.2	1.5[8]			2.2	1.7[383]
SnO_2	1.6	1.2[8]			11	40[423]
		0.98[423]				
SnS_2	1.2	1.2[8]			2.1	0.85[424]

Materials	Pred. γ	Meas. γ	Pred. α (10^{-6} K)	Meas. α (10^{-6} K)	Pred. $\kappa_{L,sine}$ (W $m^{-1}K^{-1}$)	Meas. κ_L (W $m^{-1}K^{-1}$)
$SnSe_2$	1.5	1.2[425]			3.2	1.5[383]
		1.5[8]				1.3[426]
$SnSe_2$	1.5	1.2[425]			3.2	7.1[426]
		1.7[8]				6.7[383]
SnSe	1.1	1.7[8]			2	2.3[427,428]
						0.7[429]
SnSe	1.1	1.9[8]			2	1.2[427]
						0.8[428]
						0.5[429]
SnTe	1.8	1.7[8]	16.	10[430]	2.6	2.8[299]
						1.5[383]
PbS	1.6	1.7[8]	21	20[431]	2.2	2.5[300]
		2[300]				2.5[383]
PbSe	1.5	1.7[300]	23	15[430]	1.8	1.6[300]
		2[8]				1.7[383]
PbTe	1.7	1.5[300]	19	13[430]	1.8	2.4[432]
		1.7[8]				2.3[383]
						2[300]

Materials	Pred. γ	Meas. γ	Pred. α (10^{-6} K)	Meas. α (10^{-6} K)	Pred. $\kappa_{L,sine}$ (W $m^{-1}K^{-1}$)	Meas. κ_L (W $m^{-1}K^{-1}$)
GeS	1	1.3[8]			5.4	2.0[433]
GeSe	1.5	1.3[8]			1.5	2.2[433]
						1.7[303]
GeTe	2	2.2[8]			4.5	2.6[8]
GaS	1.7	1.2[8]			3	1.1[383]
GaSe	1.7	2.2[8]			2	2[434]
		2.4[434]				
InSe	1.8	1.5[8]			2	3.7[383]
InTe	1.9	2.1[309]			1.4	0.75[309]
						1.7[383]
						0.6[383]
γ-CuCl	2	1.6[8]	43	15[133]	1.4	
				12[370]		
γ-CuBr	1.9	1.7[8]	47	15[370]	0.97	-
γ-CuI	1.9	1.2[8]	53	27[435]	0.65	
				24[436]		
AgCl	2	1.9[371]	45	30[214]	1.1	1[437]

Materials	Pred. γ	Meas. γ	Pred. α (10^{-6} K)	Meas. α (10^{-6} K)	Pred. $\kappa_{L,sine}$ (W $m^{-1}K^{-1}$)	Meas. κ_L (W $m^{-1}K^{-1}$)
		2.2[8]				
AgBr	2	2.4[8]	47	35[214]	0.8	
γ-AgI	1.9	2[8]	55	60[438]	0.47	0.4
HgI_2	1.5	2[439]			0.63	0.41[440]
		2.1[8]				
HgI_2	1.5	2[439]			0.63	0.11[440]
		2.2[8]				
PbI_2	1.8	1.3[8]			0.35	2.7[441]
TlCl	2	1.8[8]	53		0.53	
TlBr	2	2.3[8]			0.42	
β-PbF_2	2	2.1[8]				
		0.85[442]				
SbI_3	1.4	1.6[8]				
BiI_3	1.8	2.1[8]			0.13	0.36[383]
Sb_2S_3	1.1			-	3.6	1.3[383]
Sb_2Se_3	1.1			-	1.2	1[383]
Sb_2Te_3	1.7	1.5[10]			1.9	2.4[383]

Materials	Pred. γ	Meas. γ	Pred. α (10^{-6} K)	Meas. α (10^{-6} K)	Pred. $\kappa_{L,sine}$ (W $m^{-1}K^{-1}$)	Meas. κ_L (W $m^{-1}K^{-1}$)
Bi_2Te_3	1.6	1.9[8]			1.7	1.6[443]
						1.7[383]
La_3Te_4	1.6	1.8[326]			3.8	2[326]
		1.6[8]				1.1[444]
Sm_3Se_4	1.7	1.8[8]			6.5	
Mg_2Si	1.5	1.3[371]			7.8	11[445,446]
		1.1[8]				7.8[383]
Mg_2Ge	1.6	1.4[371]			6.1	10[445]
		1.3[8]				
Mg_2Sn	1.5	1.3[371]			4.1	9[445]
		1.2[8]				13[383]
Mg_2Pb	1.5	1[8]			16	11[331]
$CoSb_3$	1.6	0.95[332]			2	10[332]
		1.5[8]				
Na_2S	1.9	1.9[8]			1.8	
Na_2Se	1.9	1.5[8]			1.2	
Na_2Te	1.8	1.4[8]			0.82	
Na_2Po	1.8	1.7[8]			0.59	

Materials	Pred. γ	Meas. γ	Pred. α (10^{-6} K)	Meas. α (10^{-6} K)	Pred. $\kappa_{L,sine}$ (W m^{-1}K^{-1})	Meas. κ_L (W m^{-1}K^{-1})
Cu_2O	1.7	0.6[447]			2	4.5[448]
		0.6[75]				
Cu_2S	2	1.7[336]			0.17	0.39[336]
		2.1[8]				0.50[383]
Cu_2Se	2	2.5[8]			0.69	0.5[449]
		0.77[336]				0.54[383]
α-Ag_2S	1.5				0.62	0.9[383]
α-Ag_2Se	2			-	0.46	0.6[450]
						0.46[383]
α-Ag_2Te	1.9				0.48	0.3[342]
						0.72[383]
α-Zn_3P_2	1.5	2[8]			3.1	1.3[451]
						2.4[452]
α-Zn_3As_2	1.5	2.1[8]			1.8	1.2[383,453]
Cd_3P_2	1.3	2.1[8]			1.7	1.6[383]
						2.4[452]
α-Cd_3As_2	1.4	2.3[8]			1.1	0.4[453]

Materials	Pred. γ	Meas. γ	Pred. α (10^{-6} K)	Meas. α (10^{-6} K)	Pred. $\kappa_{L,sine}$ (W $m^{-1}K^{-1}$)	Meas. κ_L (W $m^{-1}K^{-1}$)
						1.4[383]
α-ZnP_2	1.3	1.4[8]			6.4	
$ZnAs_2$	1.3	1.3[8]			0.46	5.4[383]
β-CdP_2	1	1.6[8]				
2H-CdI_2	1.8	2.2[8]				
ZnSb	1.4	0.76[347]			1.2	1.3[454]
						1.1[383]
CdSb	0.99				5.4	1[348]
As	0.64	1.3[455]			270	17.5[456]
		1.2[8]				25.4[457]
Sb	0.64	0.94[455]			184	5[458]
		1.5[8]				
Se	0.51	1[459]				
		2.2[8]				
Te	0.51	1[460]				
		1.3[8]				
MgB_2	1.8	1.6[8]			36	16[461]
TiB_2	1.8	1.2[8]			143	120[462]

**Materials**	**Pred. γ**	**Meas. γ**	**Pred. α (10^{-6} K)**	**Meas. α (10^{-6} K)**	**Pred. $\kappa_{L,sine}$ (W m^{-1}K^{-1})**	**Meas. κ_L (W m^{-1}K^{-1})**
						96[463]
						60[462]
ZrB_2	1.9	1.1[8]			171	107[464]
						58[465]
						23[462]
HfB_2	1.8	1 [8]			98	105[466]
CaB_6	1.9	1.7[355]			22	20[467]
		1 [8]				
SrB_6	1.8	1.8[8]			10	18[467]
BaB_6	1.8	1.3[8]			14	28[468]
LaB_6	2	1.5[8]			23	35[469]
CeB_6	2	0.96[8]			55	67[469]
PrB_6	2	1.4[8]			27	44(100K)[470]
NdB_6	2	1.4[8]			25	36(100K)[470]
PmB_6	2	1.5[8]				
SmB_6	2	1.7[8]			19	15[471]
EuB_6	1.9	1.9[8]			12	15[472]
GdB_6	2	1.6[8]			21	30(100K)[470]

Materials	Pred. γ	Meas. γ	Pred. α (10^{-6} K)	Meas. α (10^{-6} K)	Pred. $\kappa_{L,sine}$ (W $m^{-1}K^{-1}$)	Meas. κ_L (W $m^{-1}K^{-1}$)
TbB_6	2	1.7[8]			19	
DyB_6	2	1.7[8]			18	
HoB_6	2	1.8[8]			17	
ErB_6	2	1.9[8]			15	
TmB_6	2	2[8]			14	
YbB_6	1.9	2.2[8]			9	
LuB_6	2	2.1[8]			12	
YB_{12}	1.3	1.8[8]			17	23[473]
ZrB_{12}	2	2.5[8]			12	10[473]
TbB_{12}	1.9	2.2[8]			8	14.9[474]
DyB_{12}	1.9	2.3[8]			7	33.0[474]
HoB_{12}	1.9	2[8]			9	20.5[474]
ErB_{12}	1.9	1.8[8]			11	12.4[474]
TmB_{12}	1.9	2[8]			9.5	16.7[474]
YbB_{12}	1.8	2.1[8]			5.9	15[475]
LuB_{12}	1.9	2.2[8]			8.4	57[474]
B_4C	1.5	2 [8]			26	27.63[462]
Li	0.99	0.98[476]	47	46[1]	5.5	

Materials	Pred. γ	Meas. γ	Pred. α (10^{-6} K)	Meas. α (10^{-6} K)	Pred. $\kappa_{L,sine}$ (W $m^{-1}K^{-1}$)	Meas. κ_L (W $m^{-1}K^{-1}$)
				51[477]		
Na	0.99	1.2[476]	50	70[156]	2.1	
				67[159]		
K	0.99	1.3[476]	59	65[478]	0.83	
				71[159]		
Rb	0.99		119	96[156]	0.17	
				100[477]		
Be	1.1		3	11[1]		
Mg	1.1		28	28[479]	5.6	
				25[1]		
Ca	1.1		43	21[1]	1.8	
				22[156]		
Al	1.1	2.3[480]	13	17[187]	20	
				21[477]		
Ga	0.89		17	18[1]	4.3	
				18[477]		
In	1.1		31	32[1]	1.6	
				24[477]		

Materials	Pred. γ	Meas. γ	Pred. α (10^{-6} K)	Meas. α (10^{-6} K)	Pred. $\kappa_{L,sine}$ (W $m^{-1}K^{-1}$)	Meas. κ_L (W $m^{-1}K^{-1}$)
Tl	1.1		26	30[1]	1.5	
Sn	0.89		9.3	7.9[481]	9.8	
Pb	1.1		32	29[1]<br>28[477]	1.5	
Cu	1.1		28	25[477]<br>24[482]	6	
Ag	1.1		21	19[1]<br>19[156]	5.8	
Au	1.1		18.4	14[1]<br>24[477]	5.3	
Zn	1.1		9.4	25[477]	16	
Cd	1.1		16	31[477]	4.9	
Ti	1.1		12	8.1[483]<br>8.6[1]	12	
Zr	0.99		6.2	5.7[1,484]<br>5.9[192]	33	
Hf	1.1		2.3	5.9[1]	57	
V	0.99	1.2[485]	4.4	6.8[156]		

Materials	Pred. γ	Meas. γ	Pred. α (10^{-6} K)	Meas. α (10^{-6} K)	Pred. $\kappa_{L,sine}$ (W $m^{-1}K^{-1}$)	Meas. κ_L (W $m^{-1}K^{-1}$)
		1.2[486]				
Nb	0.99		5	7[487]		
				5.4[156]		
Ta	0.99		3.5	6.3[1]		
				6.6[487]		
Cr	0.99	1.2[205]	3.2	5.1[404]		
				4.9[1]		
Mo	0.99	0.7[205]	3.8	4.9[156]		
				4.8[1]		
W	0.99	1.71[205]	2.7	4.5[1]		
Fe	0.99	1.7[486]	4.6	12[488]		
		1.7[489]		12[1]		
				10[477]		
Co	1.1		4.6	13[1]		
Ni	1.1		4.6	9[477]		
Pt	1.1		9.8	9.5[490]		
				10[477]		
				8.8[1]		

Materials	Pred. γ	Meas. γ	Pred. α (10^{-6} K)	Meas. α (10^{-6} K)	Pred. $\kappa_{L,sine}$ (W m^{-1}K^{-1})	Meas. κ_L (W m^{-1}K^{-1})
LiF	1.98	1.64[364]	25	35[491-493]	7.13	14.2[364]
NaF	1.98	1.83[364]	35	31[494]	3.47	10.5[364]
KF	1.99	1.58[364]	43	35[495]	1.82	7.1[364]
RbF	1.99	1.37[364]	47	31[217]	1.14	2.3[496]
LiCl	1.98	1.69[364]	40	31[217]	2.41	
NaCl	1.97	1.64[497]	47	44[214]	1.48	6.32[364]
				35[217]		6[498]
KCl	1.96	1.49[364]	54	37[499]	0.9	6.7[364]
				39[500]		
				50[501]		
RbCl	1.95	1.57[364]	56	35[214]	0.62	2.1[364]
				33[217]		
CsCl	1.96	2.24[364]	61	47[502]	0.43	0.97[364]
LiBr	1.98	1.88[364]	46	31[217]	1.35	1.8[496]
				49[214]		
NaBr	1.96	1.72[364]	51	35[217]	0.91	2.5[364]
				42[503]		
KBr	1.95	1.46[364]	58	33[504]	0.6	3.8[505]

Materials	Pred. γ	Meas. γ	Pred. α (10^{-6} K)	Meas. α (10^{-6} K)	Pred. $\kappa_{L,sine}$ (W m^{-1}K^{-1})	Meas. κ_L (W m^{-1}K^{-1})
				43[214]		
RbBr	1.95	1.43[364]	61	36[214]	0.44	3.2[364]
				34[217]		
CsBr	1.95	1.93[364]	64	39[506]	0.33	0.86[364]
LiI	1.99	2.03[364]	51	27[217]	0.8	
NaI	1.96	1.66[364]	56	45[214]	0.58	1.33[364]
KI	1.93	1.45[364]	63	37[507]	0.4	2.9[364]
				43[508]		
RbI	1.93	1.51[364]	66	37[509]	0.31	3.3[510]
				33[217]		
CsI	1.93	2[364]	69	50[436]	0.24	0.97[511]
				40[506]		
$AgSbSe_2$	1.64	3.5[8]			1.06	0.46[8]
		3.7[8]				0.42[8]
		1.96[8]				0.75[8]
$AgSbTe_2$	1.8	2.05[8]			1.04	0.7[8]
		1.87[8]				0.63[8]
$AgBiSe_2$	1.59	2.9[8]			0.87	1.03[8]

Materials	Pred. γ	Meas. γ	Pred. α (10^{-6} K)	Meas. α (10^{-6} K)	Pred. $\kappa_{L,sine}$ (W $m^{-1}K^{-1}$)	Meas. κ_L (W $m^{-1}K^{-1}$)
		2.5[8]				0.73[8]
$AgBiTe_2$	1.71	2.5[8]			0.79	0.76[8]
		1.82[8]				
$NaSbTe_2$	1.75	1.8[8]			1.75	0.74[8]
		1.6[8]				
		1.58[8]				
$NaBiTe_2$	1.79	1.6[8]			1.67	0.63[8]
		1.9[8]				
		1.76[8]				
$CuGaTe_2$	1.53	1.46[8]			1.53	2.2[8]
		1.79[8]				2.7[8]
$CuInSe_2$	1.25	1.62[8]			3.61	2.9[8]
						3.7[8]
$CuInTe_2$	1.33	0.93[8]			1.62	4.9[8]
		1.84[8]				2.5[8]
$AgGaTe_2$	1.44	1.5[8]			1.31	1.85[8]
						0.95[8]
$AgInTe_2$	1.39	1.5[8]			1.16	1.2[8]

**Materials**	**Pred. γ**	**Meas. γ**	**Pred. α (10^{-6} K)**	**Meas. α (10^{-6} K)**	**Pred. $\kappa_{L,sine}$ (W $m^{-1}K^{-1}$)**	**Meas. κ_L (W $m^{-1}K^{-1}$)**
						1.5[8]

References

1 Haynes, W. M. *CRC Handbook of Chemistry and Physics*. (CRC Press, 2015).

2 Mu, L. L., Feng, C. J. & He, H. M. Topological research on lattice energies for inorganic compounds. *Match-Commun Math Co* **56**, 97-111, doi:10.1197/jamia.M1972 (2006).

3 Kittel, C. *Introduction to Solid State Physics*. (John Wiley & Sons, 2005).

4 Nascimento, M. A. C. The nature of the chemical bond. *Journal of the Brazilian Chemical Society* **19**, doi:10.1590/s0103-50532008000200007 (2008).

5 Phillips, J. C. Ionicity of the Chemical Bond in Crystals. *Reviews of Modern Physics* **42**, 317-356, doi:10.1103/RevModPhys.42.317 (1970).

6 Levy, M. Introduction to fundamentals of elastic constants. *Experimental Methods in the Physical Sciences* **39**, 1-35, doi:10.1016/S1079-4042(01)80084-9 (2001).

7 Chung, D. H. & Buessem, W. R. The Voigt-Reuss-Hill Approximation and Elastic Moduli of Polycrystalline MgO, CaF2, β-ZnS, ZnSe, and CdTe. *J. Appl. Phys.* **38**, 2535-2540, doi:10.1063/1.1709944 (1967).

8 Chen, Z., Zhang, X., Lin, S., Chen, L. & Pei, Y. Rationalizing Phonon dispersion for lattice thermal conductivity of solids. *National Science Review* **5**, 888-894, doi:10.1093/nsr/nwy097 (2018).

9 Yan, S., Wang, Y., Tao, F. & Ren, J. High-Throughput Estimation of Phonon Thermal Conductivity from First-Principles Calculations of Elasticity. *The Journal of Physical Chemistry A* **126**, 8771-8780, doi:10.1021/acs.jpca.2c06286 (2022).

10 Toberer, E. S., Zevalkink, A. & Snyder, G. J. Phonon engineering through crystal chemistry. *J. Mater. Chem.* **21**, 15843, doi:10.1039/c1jm11754h (2011).

11 Grimsditch, M. H. & Ramdas, A. K. Brillouin scattering in diamond. *Phys. Rev. B* **11**, 3139-3148, doi:10.1103/PhysRevB.11.3139 (1975).

12 Patel, N. N. & Sunder, M. High pressure melting curve of osmium up to 35 GPa. *J. Appl. Phys.* **125**, doi:10.1063/1.5045823 (2019).

13 Liang, Y. & Zhang, B. Mechanical and electronic properties of superhardReB2. *Phys. Rev. B* **76**, doi:10.1103/PhysRevB.76.132101 (2007).

14 Makino, Y. & Miyake, S. Estimation of bulk moduli of compounds by empirical relations between bulk modulus and interatomic distance. *J. Alloys Compd.* **313**, 235-241, doi:Doi 10.1016/S0925-8388(00)01182-8 (2000).

15 Wang, Z., Ke, Y., Liu, D., Guo, H. & Bevan, K. H. Low bias short channel impurity mobility in graphene from first principles. *Appl. Phys. Lett.* **101**, doi:10.1063/1.4748326 (2012).

16 Dubrovinskaia, N., Dubrovinsky, L., Caracas, R. & Hanfland, M. Diamond as a high pressure gauge up to 2.7 Mbar. *Appl. Phys. Lett.* **97**, doi:10.1063/1.3529454 (2010).

17 Norouzzadeh, P. & Myles, C. W. A first-principles lattice dynamical study of type-I, type-II, and type-VIII silicon clathrates. *Journal of Materials Science* **51**, 4538-4548, doi:10.1007/s10853-016-9766-1 (2016).

18 Alouani, M. & Wills, J. M. Calculated optical properties of Si, Ge, and GaAs under hydrostatic pressure. *Phys Rev B Condens Matter* **54**, 2480-2490, doi:10.1103/physrevb.54.2480 (1996).

19 Zandiehnadem, F. & Ching, W. Y. Total energy, lattice dynamics, and structural phase transitions in silicon by the orthogonalized linear combination of atomic orbitals method. *Phys Rev B Condens Matter* **41**, 12162-12179, doi:10.1103/physrevb.41.12162 (1990).

20 Lheureux, D. *et al.* High-pressure ultrasonic measurements on single crystal. *High Pressure Research* **22**, 763-767, doi:10.1080/08957950212423 (2002).

21 Foulkes, W. M. Accuracy of the chemical-pseudopotential method for tetrahedral semiconductors. *Phys Rev B Condens Matter* **48**, 14216-14225, doi:10.1103/physrevb.48.14216 (1993).

22 Ma, Y. & Tse, J. S. Ab initio determination of crystal lattice constants and thermal expansion for germanium isotopes. *Solid State Commun.* **143**, 161-165, doi:10.1016/j.ssc.2007.04.043 (2007).

23 Menoni, C. S., Hu, J. Z. & Spain, I. L. Germanium at high pressures. *Phys Rev B Condens Matter* **34**, 362-368, doi:10.1103/physrevb.34.362 (1986).

24 Wang, C. Z., Yu, R. & Krakauer, H. Pressure dependence of Born effective charges, dielectric constant, and lattice

dynamics in SiC. *Phys Rev B Condens Matter* **53**, 5430-5437, doi:10.1103/physrevb.53.5430 (1996).

25 Kwiseon Kim, W. R. L. L., Benjamin Segall. Erratum: Elastic constants and related properties of tetrahedrally bonded BN, AlN, GaN, and InN. *Phys. Rev. B* **56**, 7018 (1997).

26 Pastorelli, R., Ossi, P. M., Bottani, C. E., Checchetto, R. & Miotello, A. Elastic constants of cubic boron nitride films. *Appl. Phys. Lett.* **77**, 2168-2170, doi:10.1063/1.1312201 (2000).

27 Zaoui, A. & Hassan, F. E. Full potential linearized augmented plane wave calculations of structural and electronic properties of BN, BP, BAs and BSb. *J Phys-Condens Mat* **13**, 253-262, doi:Doi 10.1088/0953-8984/13/2/303 (2001).

28 Janotti, A., Wei, S. H. & Singh, D. J. First-principles study of the stability of BN and C. *Phys. Rev. B* **64**, doi:10.1103/PhysRevB.64.174107 (2001).

29 Huang, H. *et al.* Clusters Induced Electron Redistribution to Tune Oxygen Reduction Activity of Transition Metal Single-Atom for Metal-Air Batteries. *Angew. Chem. Int. Ed. Engl.* **61**, e202116068, doi:10.1002/anie.202116068 (2022).

30 Azuhata, T., Sota, T. & Suzuki, K. Elastic constants of III-V compound semiconductors: Modification of Keyes' relation. *J Phys-Condens Mat* **8**, 3111-3119, doi:Doi 10.1088/0953-8984/8/18/005 (1996).

31 Rodríguez-Hernández, P., González-Diaz, M. & Muñoz, A. Electronic and structural properties of cubic BN and BP. *Phys. Rev. B* **51**, 14705-14708, doi:10.1103/PhysRevB.51.14705 (1995).

32 Caus, M., Dovesi, R. & Roetti, C. Pseudopotential Hartree-Fock study of seventeen III-V and IV-IV semiconductors. *Phys Rev B Condens Matter* **43**, 11937-11943, doi:10.1103/physrevb.43.11937 (1991).

33 Touat, D., Ferhat, M. & Zaoui, A. Dynamical behaviour in the boron III–V group: a first-principles study. *J. Phys.: Condens. Matter* **18**, 3647-3654, doi:10.1088/0953-8984/18/15/011 (2006).

34 Pedersen, T. G. & Pedersen, J. G. Self-consistent tight-binding model of B and N doping in graphene. *Phys. Rev. B* **87**, doi:10.1103/PhysRevB.87.155433 (2013).

35 El Haj Hassan, F. First - principles study of BNxSb1–x, BPxSb1–x and BAsxSb1–x alloys. *physica status solidi (b)* **242**, 3129-3137, doi:10.1002/pssb.200441157 (2005).

36 Greene, R. G., Luo, H., Ruoff, A. L., Trail, S. S. & DiSalvo, F. J., Jr. Pressure Induced Metastable Amorphization of BAs: Evidence for a Kinetically Frustrated Phase Transformation. *Phys. Rev. Lett.* **73**, 2476-2479, doi:10.1103/PhysRevLett.73.2476 (1994).

37 Kitamura, M., Muramatsu, S. & Harrison, W. A. Elastic properties of semiconductors studied by extended Huckel theory. *Phys Rev B Condens Matter* **46**, 1351-1357, doi:10.1103/physrevb.46.1351 (1992).

38 Deligoz, E., Colakoglu, K. & Ciftci, Y. O. Elastic, electronic, and vibrational properties of RhN compound. *Journal of Materials Science* **45**, 3720-3726, doi:10.1007/s10853-010-4415-6 (2010).

39 Bouhafs, B., Aourag, H. & Certier, M. Trends in band-gap pressure coefficients in boron compounds BP, BAs, and BSb. *J Phys-Condens Mat* **12**, 5655-5668, doi:Doi 10.1088/0953-8984/12/26/312 (2000).

40 Zaoui, A. & Ferhat, M. High-Pressure Structural Phase Transition of BSb. *physica status solidi (b)* **225**, 15-19, doi:10.1002/(sici)1521-3951(200105)225:1<15::Aid-pssb15>3.0.Co;2-7 (2001).

41 A. Balzarotti, P. L., N. Motta. Short-range order and clustering in $Ga_{1-x}Al_xAs$ and its heterostructures. *Solid State Commun.* **56**, 471-473 (1985).

42 Majewski, J. A. & Vogl, P. Simple model for structural properties and crystal stability of sp-bonded solids. *Phys Rev B Condens Matter* **35**, 9666-9682, doi:10.1103/physrevb.35.9666 (1987).

43 Lam, P. K., Cohen, M. L. & Martinez, G. Analytic relation between bulk moduli and lattice constants. *Phys Rev B Condens Matter* **35**, 9190-9194, doi:10.1103/physrevb.35.9190 (1987).

44 D.S. Rimai, R. J. S. Elastic moduli and mode gammas of GaP: Their relationship to those of other isomorphic crystals and the high pressure structural-electrical transition. *Solid State Commun.* **30**, 591-594 (1979).

45 Di Ventra, M., Peressi, M. & Baldereschi, A. Chemical and structural contributions to the valence-band offset at GaP/GaAs heterojunctions. *Phys. Rev. B* **54**, 5691-5695, doi:DOI 10.1103/PhysRevB.54.5691 (1996).

46 Ferreira, L. G., Wei, S. H. & Zunger, A. First-principles calculation of alloy phase diagrams: The renormalized-interaction approach. *Phys Rev B Condens Matter* **40**, 3197-3231, doi:10.1103/physrevb.40.3197 (1989).

47 Froyen, S. & Cohen, M. L. Structural properties of III-V zinc-blende semiconductors under pressure. *Phys. Rev. B* **28**,

3258-3265, doi:10.1103/PhysRevB.28.3258 (1983).
48 Wei, S. H. & Zunger, A. Band gaps and spin-orbit splitting of ordered and disordered AlxGa1-xAs and GaAsxSb1-x alloys. *Phys Rev B Condens Matter* **39**, 3279-3304, doi:10.1103/physrevb.39.3279 (1989).
49 Zhang, S. B. & Cohen, M. L. High-pressure phases of III-V zinc-blende semiconductors. *Phys Rev B Condens Matter* **35**, 7604-7610, doi:10.1103/physrevb.35.7604 (1987).
50 Sidorov, V. A. *et al.* Nature of semiconductor-to-metal transition and volume properties of bulk tetrahedral amorphous GaSb and GaSb-Ge semiconductors under high pressure. *Phys. Rev. Lett.* **73**, 3262-3265, doi:10.1103/PhysRevLett.73.3262 (1994).
51 D.N. Nichols, D. S. R., R.J. Sladek. Elastic anharmonicity of InP: Its relationship to the high pressure transition. *Solid State Commun.* **36**, 667-669 (1980).
52 Wei, S., Ferreira, L. G. & Zunger, A. First-principles calculation of temperature-composition phase diagrams of semiconductor alloys. *Phys Rev B Condens Matter* **41**, 8240-8269, doi:10.1103/physrevb.41.8240 (1990).
53 Cohen, M. L. Calculation of bulk moduli of diamond and zinc-blende solids. *Phys Rev B Condens Matter* **32**, 7988-7991, doi:10.1103/physrevb.32.7988 (1985).
54 Carles, R., Saint-Cricq, N., Renucci, J. B., Zwick, A. & Renucci, M. A. Resonance Raman scattering in InAs near theE1edge. *Phys. Rev. B* **22**, 6120-6126, doi:10.1103/PhysRevB.22.6120 (1980).
55 Christensen, N. E. Calculated equation of state of InAs. *Phys Rev B Condens Matter* **33**, 5096-5098, doi:10.1103/physrevb.33.5096 (1986).
56 Kobayashi, A. & Roy, A. Effects of local atomic arrangements on the densities of phonon states of $Ga_{1-x}In_xAs$, $Ga_{1-x}In_xSb$, $GaAs_{1-x}Sb_x$, and $InAs_{1-x}Sb_x$. *Phys Rev B Condens Matter* **35**, 5611-5620, doi:10.1103/physrevb.35.5611 (1987).
57 Cohen, M. L. Predicting properties and new materials. *Solid State Commun.* **92**, 45-52 (1994).
58 BENTLE, G. G. Elastic Constants of Single - Crystal BeO at Room Temperature. *J. Am. Ceram. Soc.* **49**, 3 (1966).
59 Bentle, G. G. Elastic Constants of Single-Crystal BeO at Room Temperature. *J. Am. Ceram. Soc.* **49**, 125-128, doi:10.1111/j.1151-2916.1966.tb15389.x (1966).
60 Boettger, J. C. & Wills, J. M. Theoretical structural phase stability of BeO to 1 TPa. *Phys Rev B Condens Matter* **54**, 8965-8968, doi:10.1103/physrevb.54.8965 (1996).
61 Cline, C. F., Dunegan, H. L. & Henderson, G. W. Elastic constants of hexagonal BeO, ZnS, and CdSe. *J. Appl. Phys.* **38**, 1944-1948 (1967).
62 K.J. Chang, M. L. C. Theoretical study of BeO: structural and electronic properties. *Solid State Commun.* **50**, 487-491 (1984).
63 Jog, K. N., Sanyal, S. P. & Singh, R. K. Three-body-interaction effects on the phase-transition and high-pressure behavior of divalent-metal oxides. *Phys Rev B Condens Matter* **35**, 5235-5243, doi:10.1103/physrevb.35.5235 (1987).
64 De Vita, A. *et al.* Defect energetics in MgO treated by first-principles methods. *Phys Rev B Condens Matter* **46**, 12964-12973, doi:10.1103/physrevb.46.12964 (1992).
65 B.R.K. Gupta & Goyal, R. P. Static and thermophysical properties of chalcogenide crystals with NaCl structure. *Solid State Commun.* **49**, 559-562 (1984).
66 Dobrzyniecki, J., Li, X., Nielsen, A. E. B. & Sowiński, T. Effective three-body interactions for bosons in a double-well confinement. *Physical Review A* **97**, doi:10.1103/PhysRevA.97.013609 (2018).
67 Jiro YAMAsHITA & ASANO, S. Cohesive Properties of Alkali Halides and Simple Oxides in the Local-Density Formalism. *J. Phys. Soc. Jpn.* **52**, 3506-3513 (1983).
68 Hama, J. & Suito, K. The search for a universal equation of state correct up to very high pressures. *J Phys-Condens Mat* **8**, 67-81, doi:Doi 10.1088/0953-8984/8/1/008 (1996).
69 Bylander, D. M. & Kleinman, L. Self-consistent relativistic calculation of the energy bands and cohesive energy of W. *Phys. Rev. B* **29**, 1534-1539, doi:10.1103/PhysRevB.29.1534 (1984).
70 Zupan, A., Petek, I. I., Caus, M. & Dovesi, R. Elastic constants, phase transition, and electronic structure of strontium oxide SrO: An ab initio Hartree-Fock study. *Phys Rev B Condens Matter* **48**, 799-806, doi:10.1103/physrevb.48.799 (1993).

71 Liu, L.-g. & Bassett, W. A. Effect of pressure on the crystal structure and the lattice parameters of BaO. *Journal of Geophysical Research* **77**, 4934-4937, doi:10.1029/JB077i026p04934 (1972).

72 Peselnick, L. & Meister, R. Variational Method of Determining Effective Moduli of Polycrystals: (A) Hexagonal Symmetry, (B) Trigonal Symmetry. *J. Appl. Phys.* **36**, 2879-2884, doi:10.1063/1.1714598 (1965).

73 Karzel, H. *et al.* Lattice dynamics and hyperfine interactions in ZnO and ZnSe at high external pressures. *Phys. Rev. B* **53**, 11425-11438, doi:DOI 10.1103/PhysRevB.53.11425 (1996).

74 Kucheyev, S. O., Bradby, J. E., Williams, J. S., Jagadish, C. & Swain, M. V. Mechanical deformation of single-crystal ZnO. *Appl. Phys. Lett.* **80**, 956-958, doi:Doi 10.1063/1.1448175 (2002).

75 Amrani, B., Chiboub, I., Hiadsi, S., Benmessabih, T. & Hamdadou, N. Structural and electronic properties of ZnO under high pressures. *Solid State Commun.* **137**, 395-399, doi:10.1016/j.ssc.2005.12.020 (2006).

76 Huntington, H. B. *Solid State Physics* 213-351 (1958).

77 Sisodia, P. & Verma, M. P. Polycrystalline Elastic Moduli of Some Hexagonal and Tetragonal Materials. *physica status solidi (a)* **122**, 525-534, doi:10.1002/pssa.2211220212 (1990).

78 Lee, S. G. & Chang, K. J. First-principles study of the structural properties of MgS-, MgSe-, ZnS-, and ZnSe-based superlattices. *Phys Rev B Condens Matter* **52**, 1918-1925, doi:10.1103/physrevb.52.1918 (1995).

79 Valeev, R. G. *et al.* Structure and properties of ZnS_xSe_{1-x} thin films deposited by thermal evaporation of ZnS and ZnSe powder mixtures. *Materials Research Express* **2**, doi:10.1088/2053-1591/2/2/025006 (2015).

80 Martins, J. L., Troullier, N. & Wei, S. Pseudopotential plane-wave calculations for ZnS. *Phys Rev B Condens Matter* **43**, 2213-2217, doi:10.1103/physrevb.43.2213 (1991).

81 Lin, J. S., Qteish, A., Payne, M. C. & Heine, V. V. Optimized and transferable nonlocal separable ab initio pseudopotentials. *Phys Rev B Condens Matter* **47**, 4174-4180, doi:10.1103/physrevb.47.4174 (1993).

82 Nazzal, A. & Qteish, A. Ab initio pseudopotential study of the structural phase transformations of ZnS under high pressure. *Phys Rev B Condens Matter* **53**, 8262-8266, doi:10.1103/physrevb.53.8262 (1996).

83 Vogelgesang, R., Grimsditch, M. & Wallace, J. S. The elastic constants of single crystal β-Si_3N_4. *Appl. Phys. Lett.* **76**, 982-984, doi:10.1063/1.125913 (2000).

84 Tuchman, J. A., Kim, S., Sui, Z. & Herman, I. P. Exciton photoluminescence in strained and unstrained ZnSe under hydrostatic pressure. *Phys Rev B Condens Matter* **46**, 13371-13378, doi:10.1103/physrevb.46.13371 (1992).

85 Caro, M. A., Schulz, S. & O'Reilly, E. P. Comparison of stress and total energy methods for calculation of elastic properties of semiconductors. *J Phys Condens Matter* **25**, 025803, doi:10.1088/0953-8984/25/2/025803 (2013).

86 Oliinyk, O. V. & Tatarenko, V. A. Modulated distribution of vacancies within the post-irradiated bcc metals. *Low Temperature Physics* **49**, 1229-1235, doi:10.1063/10.0021366 (2023).

87 Rajput, B. D. & Browne, D. A. Lattice dynamics of II-VI materials using the adiabatic bond-charge model. *Phys Rev B Condens Matter* **53**, 9052-9058, doi:10.1103/physrevb.53.9052 (1996).

88 El Haj Hassan, F., Amrani, B. & Bahsoun, F. Ab initio investigations of zinc chalcogenides semiconductor alloys. *Physica B: Condensed Matter* **391**, 363-370, doi:10.1016/j.physb.2006.10.020 (2007).

89 Peng, F., Liu, Q., Fu, H. & Yang, X. First-principles calculations on phase transition and elasticity of CdO under pressure. *Solid State Commun.* **148**, 6-9, doi:10.1016/j.ssc.2008.07.024 (2008).

90 Miloua, R., Miloua, F., Arbaoui, A., Kebbab, Z. & Benramdane, N. Theoretical study of phase separation in Cd1−xZnxO alloys. *Solid State Commun.* **144**, 5-9, doi:10.1016/j.ssc.2007.07.035 (2007).

91 Drickamer, H. G., Lynch, R. W., Clendenen, R. L. & Perez-Albueene, E. A. *Solid State Physics* 135-228 (1967).

92 Project, T. M. (United States: N. p., 2020).

93 Vogel, D., Kruger, P. & Pollmann, J. Ab initio electronic-structure calculations for II-VI semiconductors using self-interaction-corrected pseudopotentials. *Phys Rev B Condens Matter* **52**, 14316-14319, doi:10.1103/physrevb.52.r14316 (1995).

94 Vogel, D., Kruger, P. & Pollmann, J. Self-interaction and relaxation-corrected pseudopotentials for II-VI semiconductors. *Phys Rev B Condens Matter* **54**, 5495-5511, doi:10.1103/physrevb.54.5495 (1996).

95 Project, T. M. (United States: N. p., 2020).

96 Zakharov, O., Rubio, A. & Cohen, M. L. Calculated structural and electronic properties of CdSe under pressure. *Phys Rev B Condens Matter* **51**, 4926-4930, doi:10.1103/physrevb.51.4926 (1995).

97 Deligoz, E., Colakoglu, K. & Ciftci, Y. Elastic, electronic, and lattice dynamical properties of CdS, CdSe, and CdTe. *Physica B: Condensed Matter* **373**, 124-130, doi:10.1016/j.physb.2005.11.099 (2006).

98 Fu, X. *et al.* Alternative route for electrochemical ammonia synthesis by reduction of nitrate on copper nanosheets. *Applied Materials Today* **19**, doi:10.1016/j.apmt.2020.100620 (2020).

99 Ouendadji, S., Ghemid, S., Bouarissa, N., Meradji, H. & El Haj Hassan, F. Ab initio study of structural, electronic, phase diagram, and optical properties of CdSe x Te 1−x semiconducting alloys. *Journal of Materials Science* **46**, 3855-3861, doi:10.1007/s10853-011-5306-1 (2011).

100 J. Prieur, J. J., W. Szuszkiewicz, E. Dynowska, J. Gorecka, B. Witkowska. Elastic Constants of β-HgS. *Acta Phys. Pol., A* **94**, 3 (1998).

101 Werner, A., Hochheimer, H. D., Strössner, K. & Jayaraman, A. High-pressure x-ray diffraction studies on HgTe and HgS to 20 GPa. *Phys. Rev. B* **28**, 3330-3334, doi:10.1103/PhysRevB.28.3330 (1983).

102 Hua, X. L., Chen, X. J. & Goddard, W. A. Generalized generalized gradient approximation: An improved density-functional theory for accurate orbital eigenvalues. *Phys. Rev. B* **55**, 16103-16109, doi:DOI 10.1103/PhysRevB.55.16103 (1997).

103 Zhou, W. *et al.* High-performance and compact-designed flexible thermoelectric modules enabled by a reticulate carbon nanotube architecture. *Nat Commun* **8**, 14886, doi:10.1038/ncomms14886 (2017).

104 Comedi, D. & Kalish, R. Thermal vibrational amplitudes of constituent atoms and mechanical stability in ZnxCd1-xTe and Hg1-yCdyTe. *Phys Rev B Condens Matter* **46**, 15844-15858, doi:10.1103/physrevb.46.15844 (1992).

105 Project, T. M. (United States: N. p., 2020).

106 Project, T. M. (United States: N. p., 2020).

107 Project, T. M. (United States: N. p., 2020).

108 Project, T. M. (United States: N. p., 2020).

109 Project, T. M. (United States: N. p., 2020).

110 Project, T. M. (United States: N. p., 2020).

111 Project, T. M. (United States: N. p., 2020).

112 Project, T. M. (United States: N. p., 2020).

113 Oliver, D. W. The Elastic Moduli of MnO. *J. Appl. Phys.* **40**, 893-893, doi:10.1063/1.1657481 (1969).

114 Herrmannronzaud, D., Pavlovic, A. S. & Waintal, A. Critical and Elastic Behavior of Paramagnetic Manganese Oxide. *Physica B & C* **86**, 570-571, doi:Doi 10.1016/0378-4363(77)90599-X (1977).

115 Parsafar, G. & Mason, E. A. Universal equation of state for compressed solids. *Phys Rev B Condens Matter* **49**, 3049-3060, doi:10.1103/physrevb.49.3049 (1994).

116 Verma, A. S. & Bhardwaj, S. R. Correlation between ionic charge and ground-state properties in rocksalt and zinc blende structured solids. *J Phys Condens Matter* **18**, 8603-8612, doi:10.1088/0953-8984/18/37/018 (2006).

117 Sasaki, T. Lattice distortion of NiO under high pressure. *Phys Rev B Condens Matter* **54**, R9581-R9584, doi:10.1103/physrevb.54.r9581 (1996).

118 Li, W. *et al.* Enhanced Biological Photosynthetic Efficiency Using Light‐Harvesting Engineering with Dual‐Emissive Carbon Dots. *Adv. Funct. Mater.* **28**, doi:10.1002/adfm.201804004 (2018).

119 Project, T. M. (United States: N. p., 2020).

120 Project, T. M. (United States: N. p., 2020).

121 B.R.K. Gupta & Kumar, V. Analysis of effective compressibilities in PbS, PbSe, PbTe and SnTe. *Solid State Commun.* **45**, 745-747 (1983).

122 Kacimi, S., Zaoui, A., Abbar, B. & Bouhafs, B. Ab initio study of cubic PbS_xSe_{1-x} alloys. *J. Alloys Compd.* **462**, 135-141, doi:10.1016/j.jallcom.2007.07.068 (2008).

123 Hummer, K., Grüneis, A. & Kresse, G. Structural and electronic properties of lead chalcogenides from first principles. *Phys. Rev. B* **75**, doi:10.1103/PhysRevB.75.195211 (2007).

124 Skelton, J. M., Parker, S. C., Togo, A., Tanaka, I. & Walsh, A. Thermal physics of the lead chalcogenides PbS, PbSe, and PbTe from first principles. *Phys. Rev. B* **89**, doi:10.1103/PhysRevB.89.205203 (2014).

125 Pei, Y.-L. & Liu, Y. Electrical and thermal transport properties of Pb-based chalcogenides: PbTe, PbSe, and PbS. *J. Alloys Compd.* **514**, 40-44, doi:10.1016/j.jallcom.2011.10.036 (2012).

126 Jacobsen, M. K., Liu, W. & Li, B. Sound velocities of PbTe to 14 GPa: evidence for coupling between acoustic and optic phonons. *J Phys Condens Matter* **25**, 365402, doi:10.1088/0953-8984/25/36/365402 (2013).

127 Project, T. M. (United States: N. p., 2020).

128 Hsueh, H. C., Vass, H., Clark, S. J., Ackland, G. J. & Crain, J. High-pressure effects in the layered semiconductor germanium selenide. *Phys Rev B Condens Matter* **51**, 16750-16760, doi:10.1103/physrevb.51.16750 (1995).

129 Rabe, K. M. & Joannopoulos, J. D. Structural properties of GeTe at T=0. *Phys Rev B Condens Matter* **36**, 3319-3324, doi:10.1103/physrevb.36.3319 (1987).

130 Baki, N., Méçabih, S., Khachai, H. & Abbar, B. Spin-polarized electronic structure and magnetic properties of Ge 1−x TM x Te (TM=Mn, Fe). *J. Magn. Magn. Mater.* **345**, 222-229, doi:10.1016/j.jmmm.2013.06.048 (2013).

131 R. Banerjee, Y. P. V. Thermodynamic properties of cuprous chloride. *Solid State Commun.* **9**, 2115-2118 (1971).

132 Hull, S. & Keen, D. A. Superionic behaviour in copper(I) chloride at high pressures and high temperatures. *J Phys-Condens Mat* **8**, 6191-6198, doi:Doi 10.1088/0953-8984/8/34/009 (1996).

133 Hanson, R. C., Helliwell, K. & Schwab, C. Anharmonicity in CuCl—elastic, dielectric, and piezoelectric constants. *Phys. Rev. B* **9**, 2649-2654, doi:10.1103/PhysRevB.9.2649 (1974).

134 Amrani, B. *et al.* First principles study of structural, elastic, electronic and optical properties of CuCl, CuBr and CuI compounds under hydrostatic pressure. *Physica B: Condensed Matter* **381**, 179-186, doi:10.1016/j.physb.2006.01.447 (2006).

135 Chatterjee, S., Ghosh, S. & Basu, A. N. Unified study of the lattice-mechanical properties of copper halide crystals. *Phys. Rev. B* **28**, 3534-3549, doi:10.1103/PhysRevB.28.3534 (1983).

136 Hull, S. & Keen, D. A. High-pressure polymorphism of the copper(I) halides: A neutron-diffraction study to ~10 GPa. *Phys Rev B Condens Matter* **50**, 5868-5885, doi:10.1103/physrevb.50.5868 (1994).

137 Shen, S.-G. Calculation of the elastic properties of semiconductors. *J. Phys.: Condens. Matter* **6**, 8733, doi:10.1088/0953-8984/25/2/025803 (1994).

138 Kocak, B., Ciftci, Y. O., Colakoglu, K. & Deligoz, E. A first-principle study of the structural, elastic, lattice dynamical and thermodynamic properties of PrX (X=P, As). *Physica B: Condensed Matter* **407**, 316-323, doi:10.1016/j.physb.2011.10.038 (2012).

139 Singh, R. K. & Gupta, D. C. Phase transition and high-pressure elastic behavior of copper halides. *Phys Rev B Condens Matter* **40**, 11278-11283, doi:10.1103/physrevb.40.11278 (1989).

140 P. P. M. GROENEWEGEN & HuISZOON, C. Debye-Waller B Values of Some NaCI-Type Structures and Interionic Interaction. *Acta Cryst.* **A28**, 164 (1972).

141 Schlosser, H., Ferrante, J. & Smith, J. R. Global expression for representing cohesive-energy curves. *Phys Rev B Condens Matter* **44**, 9696-9699, doi:10.1103/physrevb.44.9696 (1991).

142 Gupta, D. C. & Singh, R. K. Pressure-induced phase transitions in silver halides. *Phys Rev B Condens Matter* **43**, 11185-11189, doi:10.1103/physrevb.43.11185 (1991).

143 W. C. Hughes & Cain, L. S. Second-order elastic constants of AgCl from 20 to 430 ℃. *Phys. Rev. B* **53**, 9 (1996).

144 Amrani, B., El Haj Hassan, F. & Zoaeter, M. First-principles study of rock-salt $AgCl_xBr_{1-x}$ alloys. *Physica B: Condensed Matter* **396**, 192-198, doi:10.1016/j.physb.2007.04.004 (2007).

145 Dandekar, D. P. Pressure Dependence of the Elastic Constants of Calcite. *Physical Review* **172**, 873-877, doi:10.1103/PhysRev.172.873 (1968).

146 Herrmannsfeldt, G. A., Chang, Y. C. & Drickamer, H. G. High-pressure studies of luminescence from GaP and GaP:N diodes. *Phys Rev B Condens Matter* **34**, 5373-5376, doi:10.1103/physrevb.34.5373 (1986).

147 Project, T. M. (United States: N. p., 2020).

148 Tamaki, A. *et al.* Transport-Properties in Valence Fluctuation Compound Sm_3Se_4. *J. Magn. Magn. Mater.* **31-4**, 383-384,

doi:Doi 10.1016/0304-8853(83)90288-3 (1983).
149 Project, T. M. (United States: N. p., 2020).
150 Georg H. Grosch, K.-J. R. Studies on AB_2-type intermetallic compounds, I. Mg_2Ge and Mg_2Sn: single-crystal structure refinement and ab initio calculations. *J. Alloys Compd.* **235**, 250-255 (1996).
151 Project, T. M. (United States: N. p., 2020).
152 Khachai, H. *et al.* First principles study of the elastic properties in X2S (X=Li, Na, K and Rb) compounds under pressure effect. *Solid State Commun.* **147**, 178-182, doi:10.1016/j.ssc.2008.05.028 (2008).
153 Project, T. M. (United States: N. p., 2020).
154 Project, T. M. (United States: N. p., 2020).
155 Peresada, G. I. On the calculation of elastic moduli of polycrystalline systems from single crystal data. *Physica Status Solidi (a)* **4**, K23-K27, doi:10.1002/pssa.2210040136 (1971).
156 Moruzzi, V. L., Janak, J. F. & Schwarz, K. Calculated thermal properties of metals. *Phys Rev B Condens Matter* **37**, 790-799, doi:10.1103/physrevb.37.790 (1988).
157 Sigalas, M., Bacalis, N. C., Papaconstantopoulos, D. A., Mehl, M. J. & Switendick, A. C. Total-energy calculations of solid H, Li, Na, K, Rb, and Cs. *Phys Rev B Condens Matter* **42**, 11637-11643, doi:10.1103/physrevb.42.11637 (1990).
158 Rasky, D. J. & Milstein, F. Pseudopotential theoretical study of the alkali metals under arbitrary pressure: Density, bulk modulus, and shear moduli. *Phys Rev B Condens Matter* **33**, 2765-2780, doi:10.1103/physrevb.33.2765 (1986).
159 MacDonald, R. A., Shukla, R. C. & Kahaner, D. K. Thermodynamic properties of bcc crystals at high temperatures: The alkali metals. *Phys. Rev. B* **29**, 6489-6499, doi:10.1103/PhysRevB.29.6489 (1984).
160 Makino, Y. Empirical determination of bulk moduli of elemental substances by pseudopotential radius. *J. Alloys Compd.* **242**, 122-128, doi:Doi 10.1016/0925-8388(96)02351-1 (1996).
161 Cohen, S. S. & Klein, M. L. Thermodynamic properties of potassium at 160 and 308 K. *Phys. Rev. B* **12**, 2984-2987, doi:10.1103/PhysRevB.12.2984 (1975).
162 Baria, J. K. Lattice mechanical properties of alkali metals. *Physica B: Condensed Matter* **371**, 280-296, doi:10.1016/j.physb.2005.10.123 (2006).
163 Zhu, M. J., Bylander, D. M. & Kleinman, L. Multiatom covalent bonding and the formation enthalpy of Na_2K. *Phys Rev B Condens Matter* **53**, 14058-14062, doi:10.1103/physrevb.53.14058 (1996).
164 V L Sliwko, P Mohn, K Schwarz & Blaha, P. The fcc - bcc structural transition: I. A band theoretical study for Li, K, Rb, Ca, Sr, and the transition metals Ti and V. *J. Phys.: Condens. Matter* **8**, 799–815 (1996).
165 Winzenick, M., Vijayakumar, V. V. & Holzapfel, W. B. High-pressure x-ray diffraction on potassium and rubidium up to 50 GPa. *Phys Rev B Condens Matter* **50**, 12381-12385, doi:10.1103/physrevb.50.12381 (1994).
166 Silversmith, D. J. & Averbach, B. L. Pressure Dependence of the Elastic Constants of Beryllium and Beryllium-Copper Alloys. *Phys. Rev. B* **1**, 567-571, doi:10.1103/PhysRevB.1.567 (1970).
167 Naimon, E. R. Third-Order Elastic Constants of Magnesium. I. Experimental. *Phys. Rev. B* **4**, 4291-4296, doi:10.1103/PhysRevB.4.4291 (1971).
168 Peng, Q., Meng, J., Li, Y., Huang, Y. & Hort, N. Effect of yttrium addition on lattice parameter, Young's modulus and vacancy of magnesium. *Materials Science and Engineering: A* **528**, 2106-2109, doi:10.1016/j.msea.2010.11.042 (2011).
169 M. Y. Chou & Cohen, M. L. AB INITIO STUDY OF THE STRUCTURAL PROPERTIES OF MAGNESIUM. *Solid State Commun.* **57**, 785-788 (1986).
170 Wedig, U., Jansen, M., Paulus, B., Rosciszewski, K. & Sony, P. Structural and electronic properties of Mg, Zn, and Cd from Hartree-Fock and density functional calculations including hybrid functionals. *Phys. Rev. B* **75**, doi:10.1103/PhysRevB.75.205123 (2007).
171 Y. P. SHARMA & MATHU, S. S. Higher order elastic constants of some face-centered cubic metals. *CANADIAN JOURNAL OF PHYSICS.* **47**, 1995 (1969).
172 Sigalas, M., Papaconstantopoulos, D. A. & Bacalis, N. C. Total energy and band structure of the 3d, 4d, and 5d metals. *Phys Rev B Condens Matter* **45**, 5777-5783, doi:10.1103/physrevb.45.5777 (1992).
173 Janak, J. F. & Williams, A. R. Giant internal magnetic pressure and compressibility anomalies. *Phys. Rev. B* **14**, 4199-

4204, doi:10.1103/PhysRevB.14.4199 (1976).
174 Anderson, M. S., Swenson, C. A. & Peterson, D. T. Experimental equations of state for calcium, strontium, and barium metals to 20 kbar from 4 to 295 K. *Phys Rev B Condens Matter* **41**, 3329-3338, doi:10.1103/physrevb.41.3329 (1990).
175 Zbigniew Witczak, V. A. G., Przemysław Witczak. Elastic properties of a polycrystalline sample of the L12 Al5CrTi2 intermetallic compound under hydrostatic pressure up to 1 GPa at room temperature. *J. Alloys Compd.* **337**, 58-63 (2002).
176 Greene, R. G., Luo, H. & Ruoff, A. L. Al as a simple solid: High pressure study to 220 GPa (2.2 Mbar). *Phys. Rev. Lett.* **73**, 2075-2078, doi:10.1103/PhysRevLett.73.2075 (1994).
177 Juan, Y. M. & Kaxiras, E. Application of gradient corrections to density-functional theory for atoms and solids. *Phys Rev B Condens Matter* **48**, 14944-14952, doi:10.1103/physrevb.48.14944 (1993).
178 Murdick, D. A. *et al.* Analytic bond-order potential for the gallium arsenide system. *Phys. Rev. B* **73**, doi:10.1103/PhysRevB.73.045206 (2006).
179 Bernasconi, M., Chiarotti, G. L. & Tosatti, E. Ab initio calculations of structural and electronic properties of gallium solid-state phases. *Phys Rev B Condens Matter* **52**, 9988-9998, doi:10.1103/physrevb.52.9988 (1995).
180 Takemura, K. Effect of pressure on the lattice distortion of indium to 56 GPa. *Phys Rev B Condens Matter* **44**, 545-549, doi:10.1103/physrevb.44.545 (1991).
181 Schulte, O. & Holzapfel, W. B. Effect of pressure on the atomic volume of Zn, Cd, and Hg up to 75 GPa. *Phys Rev B Condens Matter* **53**, 569-580, doi:10.1103/physrevb.53.569 (1996).
182 Madhava, M. R. & Saunders, G. A. Elastic constants and stability of bcc In-Tl alloys. *Phys. Rev. B* **18**, 5340-5349, doi:10.1103/PhysRevB.18.5340 (1978).
183 Cheong, B. H. & Chang, K. J. First-principles study of the structural properties of Sn under pressure. *Phys Rev B Condens Matter* **44**, 4103-4108, doi:10.1103/physrevb.44.4103 (1991).
184 Peltzer y Blanca, E. L. *et al.* Calculated static and dynamic properties of beta -Sn and Sn-O compounds. *Phys Rev B Condens Matter* **48**, 15712-15718, doi:10.1103/physrevb.48.15712 (1993).
185 Manghnani, M. H., Katahara, K. & Fisher, E. S. Ultrasonic equation of state of rhenium. *Phys. Rev. B* **9**, 1421-1431, doi:10.1103/PhysRevB.9.1421 (1974).
186 Bhikshamaiah, G., Sadanandam, J. & Suryanarayana, S. V. Debye Characteristic Temperature of Some Silver-Base Binary-Alloys. *Cryst. Res. Technol.* **29**, 713-718, doi:DOI 10.1002/crat.2170290526 (1994).
187 MacDonald, R. A. & MacDonald, W. M. Thermodynamic properties of fcc metals at high temperatures. *Phys. Rev. B* **24**, 1715-1724, doi:10.1103/PhysRevB.24.1715 (1981).
188 Sugano, M., Itoh, K., Nyilas, A. & Kiyoshi, T. Irreversible behavior of thermal expansion in Bi2212 composite wire at low temperature. *Physica C: Superconductivity and its Applications* **445-448**, 751-755, doi:10.1016/j.physc.2006.05.017 (2006).
189 Ito, M. *et al.* Effect of electronegativity on the mechanical properties of metal hydrides with a fluorite structure. *J. Alloys Compd.* **426**, 67-71, doi:10.1016/j.jallcom.2006.02.036 (2006).
190 Wang, H. *et al.* On-Demand Semiconductor Source of Entangled Photons Which Simultaneously Has High Fidelity, Efficiency, and Indistinguishability. *Phys. Rev. Lett.* **122**, 113602, doi:10.1103/PhysRevLett.122.113602 (2019).
191 Xu, J. J., Cheung, H. Y. & Shi, S. Q. Mechanical properties of titanium hydride. *J. Alloys Compd.* **436**, 82-85, doi:10.1016/j.jallcom.2006.06.107 (2007).
192 Liu, S. J., Shi, S. Q., Huang, H. & Woo, C. H. Interatomic potentials and atomistic calculations of some metal hydride systems. *J. Alloys Compd.* **330**, 64-69, doi:Doi 10.1016/S0925-8388(01)01451-7 (2002).
193 Sigalas, M. M. & Papaconstantopoulos, D. A. Calculations of the total energy, electron-phonon interaction, and Stoner parameter for metals. *Phys Rev B Condens Matter* **50**, 7255-7261, doi:10.1103/physrevb.50.7255 (1994).
194 Polatoglou, H. M. & Methfessel, M. Cohesive properties of solids calculated with the simplified total-energy functional of Harris. *Phys Rev B Condens Matter* **37**, 10403-10406, doi:10.1103/physrevb.37.10403 (1988).
195 Magerl, A., Berre, B. & Alefeld, G. Changes of the elastic constants of V, Nb, and Ta by hydrogen and deuterium. *Physica Status Solidi (a)* **36**, 161-171, doi:10.1002/pssa.2210360117 (1976).
196 Barbiellini, B., Moroni, E. G. & Jarlborg, T. Effects of Gradient Corrections on Electronic-Structure in Metals. *J Phys-*

Condens Mat **2**, 7597-7611, doi:Doi 10.1088/0953-8984/2/37/005 (1990).
197 Ito, M., Muta, H., Uno, M. & Yamanaka, S. Characteristics of niobium hydrogen solid solution. *J. Alloys Compd.* **425**, 164-168, doi:10.1016/j.jallcom.2006.01.043 (2006).
198 Kenichi, T. & Singh, A. K. High-pressure equation of state for Nb with a helium-pressure medium: Powder x-ray diffraction experiments. *Phys. Rev. B* **73**, doi:10.1103/PhysRevB.73.224119 (2006).
199 De la Peña-Seaman, O., de Coss, R., Heid, R. & Bohnen, K. P. Ab initiostudy of the structural, electronic, and phononic properties of$Nb_{1-x}Mo_x$using the self-consistent virtual-crystal approximation. *Phys. Rev. B* **76**, doi:10.1103/PhysRevB.76.174205 (2007).
200 MacDonald, R. A. & Shukla, R. C. Thermodynamic properties of bcc crystals at high temperatures: The transition metals. *Phys Rev B Condens Matter* **32**, 4961-4968, doi:10.1103/physrevb.32.4961 (1985).
201 Turchi, P. E. A., Gonis, A., Drchal, V. & Kudrnovský, J. First-principles study of stability and local order in substitutional Ta-W alloys. *Phys. Rev. B* **64**, doi:10.1103/PhysRevB.64.085112 (2001).
202 H. L. BROWN, P. E. ARMSTRON & KEMPTER, C. P. Temperature dependence of Young's modulus of a Ta-W-Hf alloy. *J. Less-Common Metals* **12**, 328-330 (1967).
203 H. L. BROWN & KEMPTER, C. P. Elastic properties of thoriated W-Mo and W-Mo-Re alloys. *J. Less-Common Metals* **12**, 166-168 (1967).
204 Zhu, M. J., Bylander, D. M. & Kleinman, L. Ab initio calculations of the cohesive energy of Mo and W and heat of formation of $MoSi_2$ and WSi_2. *Phys Rev B Condens Matter* **36**, 3182-3185, doi:10.1103/physrevb.36.3182 (1987).
205 Errandonea, D. Improving the understanding of the melting behaviour of Mo, Ta, and W at extreme pressures. *Physica B: Condensed Matter* **357**, 356-364, doi:10.1016/j.physb.2004.11.087 (2005).
206 N. MORI, M. TAKAHASHI & OOMI, G. Magnetic contribution to the bulk modulus of 3d-transition metal alloys. *J. Magn. Magn. Mater.* **31**, 135-136 (1983).
207 Gunkelmann, N., Ledbetter, H. & Urbassek, H. M. Experimental and atomistic study of the elastic properties of α′ Fe–C martensite. *Acta Mater.* **60**, 4901-4907, doi:10.1016/j.actamat.2012.05.038 (2012).
208 Dubrovinsky, L. S., Dubrovinskaia, N. A., Saxena, S. K., Rekhi, S. & LeBihan, T. Aggregate shear moduli of iron up to 90 GPa and 1100 K. *J. Alloys Compd.* **297**, 156-161, doi:Doi 10.1016/S0925-8388(99)00587-3 (2000).
209 Jarlborg, T. & Peter, M. Electronic-Structure, Magnetism and Curie Temperatures in Fe, Co and Ni. *J. Magn. Magn. Mater.* **42**, 89-99, doi:Doi 10.1016/0304-8853(84)90293-2 (1984).
210 Latham, C. D., Öberg, S., Briddon, P. R. & Louchet, F. A pseudopotential density functional theory study of native defects and boron impurities in FeAl. *J. Phys.: Condens. Matter* **18**, 8859-8876, doi:10.1088/0953-8984/18/39/016 (2006).
211 Modak, P., Verma, A. K., Rao, R. S., Godwal, B. K. & Jeanloz, R. Ab initiototal-energy and phonon calculations of Co at high pressures. *Phys. Rev. B* **74**, doi:10.1103/PhysRevB.74.012103 (2006).
212 Kong, Y., Kong, L. T. & Liu, B. X. First-principles calculations of the structural stability and magnetic property of the metastable phases in the equilibrium immiscible Co-Au system. *J Phys Condens Matter* **18**, 4345-4353, doi:10.1088/0953-8984/18/17/020 (2006).
213 H. Braul, C. A. P. Elastic and photoelastic constants of NaCl, KBr and LiF by Brillouin scattering. *Solid State Commun.* **38**, 227-230 (1981).
214 Tsay, Y.-f., Bendow, B. & Mitra, S. S. Theory of the Temperature Derivative of the Refractive Index in Transparent Crystals. *Phys. Rev. B* **8**, 2688-2696, doi:10.1103/PhysRevB.8.2688 (1973).
215 Shanker, J., Agrawal, D. P. & Singh, R. P. Additivity Rule for the Softness Parameter in Alkali-Halides. *Solid State Commun.* **31**, 765-768, doi:Doi 10.1016/0038-1098(79)90785-3 (1979).
216 Sangster, M. J. L. CONSISTENT SHELL MODEL PARAMETERS FOR THE ROCKSALT STRUCTURE ALKALI HALIDES. *Solid State Commun.* **18**, 67-70 (1976).
217 Boyer, L. L. First-principles equation-of-state calculations for alkali halides. *Phys. Rev. B* **23**, 3673-3685, doi:10.1103/PhysRevB.23.3673 (1981).
218 Kohler, U., Johannsen, P. G. & Holzapfel, W. B. Equation-of-state data for CsCl-type alkali halides. *J Phys-Condens Mat*

9, 5581-5592, doi:Doi 10.1088/0953-8984/9/26/007 (1997).
219 Cortona, P. Direct determination of self-consistent total energies and charge densities of solids: A study of the cohesive properties of the alkali halides. *Phys Rev B Condens Matter* **46**, 2008-2014, doi:10.1103/physrevb.46.2008 (1992).
220 Goyal, S. C. & Verma, M. P. Dielectric behavior of allkali halides and the second-neighbor interaction. *Phys. Rev. B* **9**, 3609-3613, doi:10.1103/PhysRevB.9.3609 (1974).
221 van Schilfgaarde, M. & Sher, A. Tight-binding theory and elastic constants. *Phys Rev B Condens Matter* **36**, 4375-4382, doi:10.1103/physrevb.36.4375 (1987).
222 Gong, Z., Horton, G. K. & Cowley, E. R. Monte Carlo and lattice-dynamics studies of the thermal and elastic properties of a rigid-ion model of sodium chloride. *Phys Rev B Condens Matter* **38**, 10820-10829, doi:10.1103/physrevb.38.10820 (1988).
223 Swenson, C. A. Volume dependence of the Gruneisen parameter: Alkali metals and NaCl. *Phys Rev B Condens Matter* **31**, 1150-1152, doi:10.1103/physrevb.31.1150 (1985).
224 O. D. Slagle, H. A. M. Temperature Dependence of the Elastic Constants of the Alkali Halides. I. NaCl, KCl, and KBr. *J. Appl. Phys.* **38**, 437–446 (1967).
225 Cowley, E. R., Gross, J., Gong, Z. & Horton, G. K. Cell-cluster and self-consistent calculations for a model sodium chloride crystal. *Phys Rev B Condens Matter* **42**, 3135-3141, doi:10.1103/physrevb.42.3135 (1990).
226 Gong, Z., Horton, G. K. & Cowley, E. R. van der Waals forces in a Monte Carlo and lattice-dynamics study of the thermal and elastic properties of a rigid-ion model of potassium chloride. *Phys Rev B Condens Matter* **40**, 3294-3300, doi:10.1103/physrevb.40.3294 (1989).
227 N. V. Chandra Shekar & Rajan, K. G. Kinetics of pressure induced structural phase transitions—A review. *Bull. Mater. Sci.* **24**, 1 (2001).
228 Kantorovich, L. N. Thermoelastic properties of perfect crystals with nonprimitive lattices. I. General theory. *Phys Rev B Condens Matter* **51**, 3520-3534, doi:10.1103/physrevb.51.3520 (1995).
229 R.W. Roberts, C. S. S. Ultrasonic parameters in the born model of the sodium and potassium halides. *J. Phys. Chem. Solids* **31**, 619-634 (1970).
230 R.J. Wallat, J. H. Third order elastic constants of rubidium chloride. *J. Phys. Chem. Solids* **38**, 1227-1231 (1977).
231 R.W. Roberts, C. S. S. Ultrasonic parameters in the born model of the rubidium halides. *J. Phys. Chem. Solids* **31**, 2397-2400 (1970).
232 Satpathy, S. Electron energy bands and cohesive properties of CsCl, CsBr, and CsI. *Phys Rev B Condens Matter* **33**, 8706-8715, doi:10.1103/physrevb.33.8706 (1986).
233 Z. P. Chang, G. R. B. Nonlinear Pressure Dependence of Elastic Constants and Fourth-Order Elastic Constants of Cesium Halides. *Phys. Rev. Lett.* **19**, 1381 (1967).
234 Charles S. Smith, L. S. C. Temperature derivatives at constant volume of the elastic constants of the alkali halides. *J. Phys. Chem. Solids* **41**, 199-203 (1980).
235 D. B. Sirdeshmukh, K. G. S. Bulk modulus ‐ volume relationship for some crystals with a rock salt structure. *J. Appl. Phys.* **59**, 276–277 (1986).
236 Krishnamurthy, C. V. & Murti, Y. V. Solid solutions of alkali halide compounds. I. Configurational and vibrational contributions. *Phys Rev B Condens Matter* **43**, 14206-14218, doi:10.1103/physrevb.43.14206 (1991).
237 Z.P. Chang, G. R. B. Pressure dependence of the elastic constants of RbCl, RbBr and RbI. *J. Phys. Chem. Solids* **32**, 27-40 (1971).
238 A. Jayaraman, B. B., R. G. Maines, H. Bach. Effective ionic charge and bulk modulus scaling in rocksalt-structured rare-earth compounds. *Phys. Rev. B* **26**, 3347 (1982).
239 O. D. Slagle, H. A. M. Temperature Dependence of the Elastic Constants of the Alkali Halides. III. CsCl, CsBr, and CsI. *J. Appl. Phys.* **38**, 451–458 (1967).
240 Rao, B. S. & Sanyal, S. P. Structural and elastic properties of sodium halides at high pressure. *Phys Rev B Condens Matter* **42**, 1810-1816, doi:10.1103/physrevb.42.1810 (1990).
241 J R Drabble, R. E. B. S. The third-order elastic constants of potassium chloride, sodium chloride and lithium fluoride.

Proc. Phys. Soc. **92** 1090 (1967).

242 Project, T. M. (United States: N. p., 2020).

243 Project, T. M. (United States: N. p., 2020).

244 Project, T. M. (United States: N. p., 2020).

245 Project, T. M. (United States: N. p., 2020).

246 Verma, A. S. & Bhardwaj, S. R. Correlation between ionic charge and the mechanical properties of complex structured solids. *J. Phys.: Condens. Matter* **19**, doi:10.1088/0953-8984/19/2/026213 (2007).

247 Project, T. M. (United States: N. p., 2020).

248 Fernández, B. & Wasim, S. M. Sound Velocities and Elastic Moduli in CuInTe2 and CuInSe2. *physica status solidi (a)* **122**, 235-242, doi:10.1002/pssa.2211220122 (1990).

249 Neumann, H. Bulk Modulus-Volume Relationship in Ternary Chalcopyrite Compounds. *physica status solidi (a)* **96**, K121-K125, doi:10.1002/pssa.2210960245 (1986).

250 Project, T. M. (United States: N. p., 2020).

251 Verma, A. S. & Bhardwaj, S. R. Mechanical and optical properties of AIIBIVC2V and AIBIIIC2VI semiconductors. *physica status solidi (b)* **243**, 2858-2863, doi:10.1002/pssb.200642140 (2006).

252 Wang, M. & Lin, S. Ballistic Thermal Transport in Carbyne and Cumulene with Micron-Scale Spectral Acoustic Phonon Mean Free Path. *Sci Rep* **5**, 18122, doi:10.1038/srep18122 (2015).

253 Bosak, A., Krisch, M., Mohr, M., Maultzsch, J. & Thomsen, C. Elasticity of single-crystalline graphite: Inelastic x-ray scattering study. *Phys. Rev. B* **75**, doi:10.1103/PhysRevB.75.153408 (2007).

254 McSkimin, H. J., Andreatch, P. & Glynn, P. The Elastic Stiffness Moduli of Diamond. *J. Appl. Phys.* **43**, 985-987, doi:10.1063/1.1661318 (1972).

255 Ezz-El-Arab, M., Galperin, B., Brielles, J. & Vodar, B. Variation des vitesses de propagation des ultrasons dans le silicium monocristallin entre 25 °et 830 °C. *Solid State Commun.* **6**, 387-390 (1968).

256 McSkimin, H. J. & Andreatch, P. Elastic Moduli of Germanium Versus Hydrostatic Pressure at 25.0 ℃ and −195.8 ℃. *J. Appl. Phys.* **34**, 651, doi:10.1063/1.1729323 (1963).

257 SCHREIBER E, S. N. Sound velocity and Poisson's ratio of SiC]Elastic Constants of Silicon Carbide. *J. Am. Ceram. Soc.* **49(6)**, 342-342 (1966).

258 Duclaux, L., Nysten, B., Issi, J. P. & Moore, A. W. Structure and low-temperature thermal conductivity of pyrolytic boron nitride. *Phys. Rev. B* **46**, 3362-3367, doi:10.1103/PhysRevB.46.3362 (1992).

259 Xia, H., Xia, Q. & Ruoff, A. L. BP at megabar pressures and its equation of state to 110 GPa. *J. Appl. Phys.* **74**, 1660, doi:10.1063/1.354817 (1993).

260 Broido, D. A., Lindsay, L. & Reinecke, T. L. Ab initiostudy of the unusual thermal transport properties of boron arsenide and related materials. *Phys. Rev. B* **88**, doi:10.1103/PhysRevB.88.214303 (2013).

261 Gerlich D, D. S. L., Slack G A. Elastic properties of aluminum nitride. *J. Phys. Chem. Solids* **47(5)**, 437-441 (1986).

262 Adachi, S. GaAs, AlAs, and AlxGa1−xAs Material parameters for use in research and device applications. *J. Appl. Phys.* **58**, R1, doi:10.1063/1.336070 (1985).

263 Bolef, D. I. & Menes, M. Elastic Constants of Single-Crystal Aluminum Antimonide. *J. Appl. Phys.* **31**, 1426, doi:10.1063/1.1735857 (1960).

264 Yamaguchi M, Y. T., Azuhata T, et al. Brillouin scattering study of gallium nitride elastic stiffness constants. *J. Phys.: Condens. Matter* **9(1)**, 241 (1997).

265 Weil, R. & Groves, W. O. The Elastic Constants of Gallium Phosphide. *J. Appl. Phys.* **39**, 4049-4051, doi:10.1063/1.1656922 (1968).

266 Blakemore, J. Semiconducting and other major properties of gallium arsenide. *J. Appl. Phys.* **53**, R123-R181 (1982).

267 Brazhkin, V., Lyapin, A., Goncharova, V., Stal’gorova, O. & Popova, S. Elastic softness of amorphous tetrahedrally bonded GaSb and (Ge 2) 0.27 (GaSb) 0.73 semiconductors. *Phys. Rev. B* **56**, 990 (1997).

268 Sheleg A U & A, S. V. Determination of elastic constants of hexagonal crystals from measured values of dynamic atomic displacements. *Inorg. Mater.* **15**, 1257-1260 (1979).

269 Hickernell, F. & Gayton, W. Elastic Constants of Single‐Crystal Indium Phosphide. *J. Appl. Phys.* **37**, 462-462 (1966).
270 Gerlich, D. Elastic Constants of Single‐Crystal Indium Arsenide. *J. Appl. Phys.* **34**, 2915-2915, doi:10.1063/1.1729833 (1963).
271 Drabble, J. & Brammer, A. The third-order elastic constants of indium antimonide. *Proceedings of the Physical Society* **91**, 959 (1967).
272 Sumino, Y., Ohno, I., Goto, T. & Kumazawa, M. Measurement of elastic constants and internal frictions on single-crystal MgO by rectangular parallelepiped resonance. *Journal of Physics of the Earth* **24**, 263-273 (1976).
273 Chang, Z. & Graham, E. Elastic properties of oxides in the NaCl-structure. *J. Phys. Chem. Solids* **38**, 1355-1362 (1977).
274 Carlotti, G., Fioretto, D., Socino, G. & Verona, E. Brillouin scattering determination of the whole set of elastic constants of a single transparent film of hexagonal symmetry. *J. Phys.: Condens. Matter* **7**, 9147 (1995).
275 Zarembovitch, A. Étude des constantes élastiques de la blende et de leur variation avec la température. *Journal de Physique* **24**, 1097-1102 (1963).
276 Bieniewski, T. & Czyzak, S. Refractive indexes of single hexagonal ZnS and CdS crystals. *JOSA* **53**, 496-497 (1963).
277 Chang E & R, B. G. Pressure dependence of single crystal elastic constants and anharmonic properties of wurtzite. *J. Phys. Chem. Solids* **34(9)**, 1543-1563 (1973).
278 Lee, B. H. Elastic Constants of ZnTe and ZnSe between 77°–300°K. *J. Appl. Phys.* **41**, 2984-2987, doi:10.1063/1.1659349 (1970).
279 Bijalwan, R., Ram, P. & Tiwari, M. Lattice thermal conductivity of II-VI compounds. *Journal of Physics C: Solid State Physics* **16**, 2537 (1983).
280 Subhadra, K. & Sirdeshmukh, D. X-ray determination of the mean Debye-Waller factors, amplitudes of vibrations and the Debye temperatures of CdO, PbS and MnS. *Pramana* **10**, 357-360 (1978).
281 Holland, M. G. Phonon Scattering in Semiconductors From Thermal Conductivity Studies. *Physical Review* **134**, A471-A480, doi:10.1103/PhysRev.134.A471 (1964).
282 Bonello B & B., F. Elastic constants of CdSe at low temperature. *J. Phys. Chem. Solids* **54(2)**, 209-212 (1993).
283 Kumazaki, K. Elastic properties and ionicity of zero‐gap semiconductors. *physica status solidi (a)* **33**, 615-623 (1976).
284 Ford P J, M. A. J., Saunders G A, et al. The effects of pressure on the elastic constants of mercury selenide up to the phase transition. *Journal of Physics C: Solid State Physics* **15(4)**, 657 (1982).
285 Vekilov, Y. K. & Rusakov, A. ELASTIC CONSTANTS AND LATTICE DYNAMICS OF SOME A 2 B 6 COMPOUNDS. *SOV PHYS SOLID STATE* **13**, 956-960 (1971).
286 Hull, S., Farley, T. W. D., Hayes, W. & Hutchings, M. The elastic properties of lithium oxide and their variation with temperature. *J. Nucl. Mater.* **160**, 125-134 (1988).
287 Zhang, L. & Chopelas, A. Sound velocity of Al2O3 to 616 kbar. *Phys Earth Planet In* **87**, 77-83 (1994).
288 Rossi, L. R. & Lawrence, W. G. Elastic Properties of Oxide Solid Solutions: The System Al2O3− Cr2O3. *J. Am. Ceram. Soc.* **53**, 604-608 (1970).
289 Lin, J.-F. *et al.* Phonon density of states of Fe2O3across high-pressure structural and electronic transitions. *Phys. Rev. B* **84**, doi:10.1103/PhysRevB.84.064424 (2011).
290 Guo Z, V. A., Wu X, et al. Anisotropic thermal conductivity in single crystal β-gallium oxide. *Appl. Phys. Lett.* **106(11)**, 111909 (2015).
291 Dole, S., Hunter, O. & Calderwood, F. Elastic properties of polycrystalline scandium and thulium sesquioxides. *J. Am. Ceram. Soc.* **60**, 167-168 (1977).
292 Badehian, H. A., Salehi, H., Ghoohestani, M. & Ching, W. Y. First-Principles Study of Elastic, Structural, Electronic, Thermodynamical, and Optical Properties of Yttria (Y2O3) Ceramic in Cubic Phase. *J. Am. Ceram. Soc.* **96**, 1832-1840, doi:10.1111/jace.12259 (2013).
293 Uchida, N. & Saito, S. Elastic constants and acoustic absorption coefficients in MnO, CoO, and NiO single crystals at room temperature. *The Journal of the Acoustical Society of America* **51**, 1602-1605 (1972).
294 Xu, Y. *et al.* Performance optimization and single parabolic band behavior of thermoelectric MnTe. *J. Mater. Chem. A* **5**, 19143-19150, doi:10.1039/c7ta04842d (2017).

295 Ding, G., Gao, G. & Yao, K. High-efficient thermoelectric materials: The case of orthorhombic IV-VI compounds. *Scientific reports* **5** (2015).

296 Chang, E. & Graham, E. K. The elastic constants of cassiterite SnO2and their pressure and temperature dependence. *Journal of Geophysical Research* **80**, 2595-2599, doi:10.1029/JB080i017p02595 (1975).

297 Lagnier, R., Ayache, C., Harbec, J.-Y., Jandl, S. & Jay-Gerin, J.-P. Specific heat of the semiconducting layered compound SnSe2 at low temperatures. *Solid State Commun.* **48**, 65-68 (1983).

298 Li, C. W. *et al.* Orbitally driven giant phonon anharmonicity in SnSe. *Nature Physics* **11**, 1063-1069, doi:10.1038/nphys3492 (2015).

299 Li, W. *et al.* Band and scattering tuning for high performance thermoelectric $Sn_{1-x}Mn_{x}Te$ alloys. *Journal of Materiomics* **1**, 307-315, doi:10.1016/j.jmat.2015.09.001 (2015).

300 Wang, H., Schechtel, E., Pei, Y. & Snyder, G. J. High Thermoelectric Efficiency of n-type PbS. *Advanced Energy Materials* **3**, 488-495, doi:10.1002/aenm.201200683 (2013).

301 Chen, Z. *et al.* Vacancy-induced dislocations within grains for high-performance PbSe thermoelectrics. *Nat Commun* **8**, 13828, doi:10.1038/ncomms13828 (2017).

302 Madelung, O. in *Semiconductors: Data Handbook* 566-605 (Springer, 2004).

303 Zhang, X. *et al.* Thermoelectric properties of GeSe. *Journal of Materiomics* **2**, 331-337, doi:10.1016/j.jmat.2016.09.001 (2016).

304 Zhang, X. *et al.* GeTe Thermoelectrics. *Joule*, doi:10.1016/j.joule.2020.03.004 (2020).

305 Powell, B., Jandl, S., Brebner, J. & Levy, F. Anisotropic phonon dispersion in GaS. *Journal of Physics C: Solid State Physics* **10**, 3039 (1977).

306 Khalilov K M, R. K. I. PREPARATION OF A GASE SINGLE CRYSTAL AND DETERMINATION OF ITS ELASTIC PARAMETERS. *Soviet Physics Crystallography, Ussr* **11(6)**, 786-&. (1967).

307 Bhan S, S. K. Kristallstruktur von Tl5Te3 und Tl2Te3. *Journal of the Less Common Metals* **20(3)** (1970).

308 Gatulle M, F. M., Chevy A. Elastic constants of the layered compounds GaS, GaSe, InSe, and their pressure dependence I. Experimental part. *physica status solidi (b)* **119(1)**, 327-336 (1983).

309 Jana, M. K., Pal, K., Waghmare, U. V. & Biswas, K. The Origin of Ultralow Thermal Conductivity in InTe: Lone-Pair-Induced Anharmonic Rattling. *Angew. Chem. Int. Ed. Engl.* **55**, 7792-7796, doi:10.1002/anie.201511737 (2016).

310 Every, A. & McCurdy, A. in *Second and Higher Order Elastic Constants* 593-606 (Springer).

311 Prevot, B., Hennion, B. & Dorner, B. Phonon spectrum of CuCl at 4.2 K and its temperature dependence. *Journal of Physics C: Solid State Physics* **10**, 3999 (1977).

312 Hanson, R., Hallberg, J. & Schwab, C. Elastic and piezoelectric constants of the cuprous halides. *Appl. Phys. Lett.* **21**, 490-492 (1972).

313 Hughes W C, C. L. S. Second-order elastic constants of AgCl from 20 to 430 °C. *Phys. Rev. B* **53(9)**, 5174 (1996).

314 Dorner, B., Von der Osten, W. & Buhrer, W. Lattice dynamics of AgBr. *Journal of Physics C: Solid State Physics* **9**, 723 (1976).

315 Shen, S.-G. Calculation of the elastic properties of semiconductors. *J. Phys.: Condens. Matter* **6**, 8733 (1994).

316 Prevot B, S. C. & B., D. Phonon Dispersion in Red HgI2. *physica status solidi (b)* **88(1)**, 327-333 (1978).

317 Dorner, B., Ghosh, R. & Harbeke, G. Phonon Dispersion in the Layered Compound PbI2. *physica status solidi (b)* **73**, 655-659 (1976).

318 Kodama, M., Saito, S. & Minomura, S. Pressure Dependence of the Elastic Constants of TlCl. *J. Phys. Soc. Jpn.* **33**, 1361-1371 (1972).

319 Morse G E, L. A. W. The temperature and pressure dependence of the elastic constants of thallium bromide ag. *J. Phys. Chem. Solids* **28(6)**, 939-950 (1967).

320 Dickens M H, H. W., Hutchings M T, et al. Neutron scattering studies of acoustic phonon modes in PbF2 up to high temperatures. *Journal of Physics C: Solid State Physics* **12(1)**, 17 (1979).

321 Tato, E., Komatsu, T. & Kaifu, Y. Determination of Elastic Constants of BiI3 and SbI3 Layered Crystals by Means of Brillouin Scattering. *J. Phys. Soc. Jpn.* **54**, 3597-3604 (1985).

322 Grigas, I., VI, S., VF, K. & ORLYUKAS, A. Vol. 14 928-& (MEZHDUNARODNAYA KNIGA 39 DIMITROVA UL., 113095 MOSCOW, RUSSIA, 1972).

323 Abrikosov N K, T. A. Binary Semiconductors.(Book Reviews: Semiconducting II-VI, IV-VI, and V-VI Compounds)[*Science* **166**, 1261 (1969).

324 Yang, F., Ikeda, T., Snyder, G. J. & Dames, C. Effective thermal conductivity of polycrystalline materials with randomly oriented superlattice grains. *J. Appl. Phys.* **108**, 034310, doi:10.1063/1.3457334 (2010).

325 Kullmann, W. *et al.* Lattice dynamics and phonon dispersion in the narrow gap semiconductor Bi2Te3 with sandwich structure. *physica status solidi (b)* **162**, 125-140 (1990).

326 May, A. F., Fleurial, J.-P. & Snyder, G. J. Thermoelectric performance of lanthanum telluride produced via mechanical alloying. *Phys. Rev. B* **78**, doi:10.1103/PhysRevB.78.125205 (2008).

327 Tamaki, A. *et al.* Elastic properties of SmB6 and Sm3Se4. *J. Magn. Magn. Mater.* **47**, 469-471 (1985).

328 Whitten, W., Chung, P. & Danielson, G. Elastic constants and lattice vibration frequencies of Mg2Si. *J. Phys. Chem. Solids* **26**, 49-56 (1965).

329 Chung P L, W. W. B., Danielson G C. Lattice dynamics of Mg2Ge. *J. Phys. Chem. Solids* **26(12)**, 1753-1760 (1965).

330 Davis, L., Whitten, W. & Danielson, G. Elastic constants and calculated lattice vibration frequencies of Mg2Sn. *J. Phys. Chem. Solids* **28**, 439-447 (1967).

331 Duan, Y., Sun, Y., Feng, J. & Peng, M. Thermal stability and elastic properties of intermetallics Mg 2 Pb. *Physica B: Condensed Matter* **405**, 701-704 (2010).

332 Caillat, T., Borshchevsky, A. & Fleurial, J. P. Properties of single crystalline semiconducting CoSb3. *J. Appl. Phys.* **80**, 4442-4449 (1996).

333 Kalarasse, F. & Bennecer, B. Elastic properties and lattice dynamics of alkali chalcogenide compounds Na2S, Na2Se and Na2Te. *Computational Materials Science* **50**, 1806-1810, doi:10.1016/j.commatsci.2011.01.019 (2011).

334 Baki, N. *et al.* Elastic, Electronic, Optical and Thermal Properties of Na2Po: An Ab Initio Study. *J. Electron. Mater.* **45**, 435-443, doi:10.1007/s11664-015-4119-4 (2015).

335 Manghnani, M., Brower, W. & Parker, H. Anomalous elastic behavior in Cu2O under pressure. *physica status solidi (a)* **25**, 69-76 (1974).

336 He, Y. *et al.* High thermoelectric performance in non-toxic earth-abundant copper sulfide. *Adv. Mater.* **26**, 3974-3978, doi:10.1002/adma.201400515 (2014).

337 (!!! INVALID CITATION !!!).

338 A. Danilkin, S., Yethiraj, M. & J. Kearley, G. Phonon Dispersion in Superionic Copper Selenide: Observation of Soft Phonon Modes in Superionic Phase Transition. *J. Phys. Soc. Jpn.* **79**, 25-28, doi:10.1143/jpsjs.79sa.25 (2010).

339 Danilkin, S. A. *et al.* Crystal structure and lattice dynamics of superionic copper selenide Cu2−δSe. *J. Alloys Compd.* **361**, 57-61, doi:10.1016/s0925-8388(03)00439-0 (2003).

340 Honma, K. & Iida, K. Specific Heat of the Superionic Conductor Ag2Te x S1-x. *J. Phys. Soc. Jpn.* **54**, 2218-2228 (1985).

341 Pasternak, M., Benczer-Koller, N., Yang, T., Ruel, R. & Herber, R. H. Impurity-induced local disorder in the ordered state of the superionic conductorβ-Ag2Se. *Phys. Rev. B* **27**, 2055-2058, doi:10.1103/PhysRevB.27.2055 (1983).

342 Pei, Y., Heinz, N. A. & Snyder, G. J. Alloying to Increase the Band Gap for Improving Thermoelectric Properties of Ag2Te. *J. Mater. Chem.* **21**, 18256-18260 (2011).

343 Opilska, L. & Opilski, A. Relation between energy gap and velocity of ultrasonic wave propagation in AIIBV group semiconductors. *physica status solidi (a)* **35** (1976).

344 Demidenko, A. *et al.* Heat capacity and thermodynamic properties of A 3 2 B 2 5 compounds. *Izvestiya Akademii Nauk SSSR, Neorganicheskie Materialy* **13**, 214-216 (1977).

345 Soshnikov, L., Trukhan, V., Haliakevich, T. & Soshnikava, H. Dielectric and elastic properties of CdP2, ZnP2 and ZnAs2 single crystals. *Moldavian Journal of the Physical Sciences* **4**, 201-210 (2005).

346 Sandercock, J. Some recent developments in Brillouin-scattering. *Rca Review* **36**, 89-107 (1975).

347 Bjerg, L., Iversen, B. B. & Madsen, G. K. H. Modeling the thermal conductivities of the zinc antimonides ZnSb and Zn4Sb3. *Phys. Rev. B* **89**, doi:10.1103/PhysRevB.89.024304 (2014).

348 Madelung, O. in *Semiconductors: Data Handbook* 476-506 (Springer, 2004).

349 Pace, N. & Saunders, G. Elastic wave propagation in the group VB semimetals. *J. Phys. Chem. Solids* **32**, 1585-1601 (1971).

350 de Bretteville Jr, A., Cohen, E. R., Ballato, A. D., Greenberg, I. N. & Epstein, S. Least-Squares Determination of the Elastic Constants of Antimony and Bismuth. *Physical Review* **148**, 575 (1966).

351 Royer, D. & Dieulesaint, E. Elastic and piezoelectric constants of trigonal selenium and tellurium crystals. *J. Appl. Phys.* **50**, 4042-4045 (1979).

352 Lin, S. *et al.* Tellurium as a high-performance elemental thermoelectric. *Nat Commun* **7**, 10287, doi:10.1038/ncomms10287 (2016).

353 Liu, Z.-l., Chen, X.-R. & Wang, Y.-L. First-principles calculations of elastic properties of LiBC. *Physica B: Condensed Matter* **381**, 139-143, doi:10.1016/j.physb.2005.12.264 (2006).

354 Wiley, D., Manning, W. & Hunter, O. Elastic properties of polycrystalline TiB2, ZrB2 and HfB2 from room temperature to 1300 K. *Journal of the Less Common Metals* **18**, 149-157 (1969).

355 Wei, Y.-K., Yu, J.-X., Li, Z.-G., Cheng, Y. & Ji, G.-F. Elastic and thermodynamic properties of CaB6 under pressure from first principles. *Physica B: Condensed Matter* **406**, 4476-4482, doi:10.1016/j.physb.2011.09.011 (2011).

356 Huang, B., Duan, Y.-H., Sun, Y., Peng, M.-J. & Chen, S. Electronic structures, mechanical and thermodynamic properties of cubic alkaline-earth hexaborides from first principles calculations. *J. Alloys Compd.* **635**, 213-224, doi:10.1016/j.jallcom.2015.02.128 (2015).

357 Lemis-Petropoulos, P., Kapaklis, V., Peikrishvili, A. & Politis, C. Characterization of B4C and LaB6 by Ultrasonics and X-rays diffraction. *Int. J. Mod Phys B* **17**, 2781-2788 (2003).

358 Lüthi, B. *et al.* Elastic and magnetoelastic effects in CeB 6. *Zeitschrift für Physik B Condensed Matter* **58**, 31-38 (1984).

359 Jie, D. *et al.* Elastic properties and electronic structures of lanthanide hexaborides. *Chinese Physics B* **24**, 096201 (2015).

360 Nakamura, S., Goto, T., Kasaya, M. & Kunii, S. Electron-Strain Interaction in ValenceFluctuation Compound SmB6. *J. Phys. Soc. Jpn.* **60**, 4311-4318 (1991).

361 Zherlitsyn, S. *et al.* Elastic properties of ferromagnetic EuB6. *The European Physical Journal B-Condensed Matter and Complex Systems* **22**, 327-333 (2001).

362 Odintsov, V. V. & Korin', O. V. Influence of the Crystalline Structure on the Mechanical Properties of Dodecaborides of Rare-Earth Metals and Zirconium. *Mater. Sci.* **51**, 576-582, doi:10.1007/s11003-016-9878-4 (2016).

363 Molecular Acoustics · 5.1.2 Metals: Datasheet from Landolt-Börnstein - Group II Molecules and Radicals · Volume 5: "Molecular Acoustics" in SpringerMaterials (https://doi.org/10.1007/10201218_69) (Springer-Verlag Berlin Heidelberg).

364 D. B. Sirdeshmukh , L. Sirdeshmukh & Subhadra, K. G. *Alkali Halides*. (Springer Berlin).

365 Morelli D T & A, S. G. *High lattice thermal conductivity solids*. (Springer New York, 2006).

366 Mounet, N. & Marzari, N. First-principles determination of the structural, vibrational and thermodynamic properties of diamond, graphite, and derivatives. *Phys. Rev. B* **71**, doi:10.1103/PhysRevB.71.205214 (2005).

367 Slack, G. A. Thermal Conductivity of Pure and Impure Silicon, Silicon Carbide, and Diamond. *J. Appl. Phys.* **35**, 3460-3466, doi:10.1063/1.1713251 (1964).

368 Slack, G. A. Nonmetallic crystals with high thermal conductivity. *J. Phys. Chem. Solids* **34**, 321-335 (1973).

369 Reeber, R. R. Thermal expansion of some group IV elements and ZnS. *Physica Status Solidi (a)* **32**, 321-331, doi:10.1002/pssa.2210320138 (1975).

370 Berger, L. I. *Semiconductor Materials*. (CRC Press, 1996).

371 Slack, G. A. The thermal conductivity of nonmetallic crystals. *Solid state physics* **34**, 1-71, doi:10.1016/S0081-1947(08)60359-8 (1979).

372 Glassbrenner, C. J. & Slack, G. A. Thermal Conductivity of Silicon and Germanium from 3 °K to the Melting Point. *Physical Review* **134**, A1058-A1069, doi:10.1103/PhysRev.134.A1058 (1964).

373 Steigmeier, E. & Kudman, I. Acoustical-optical phonon scattering in Ge, Si, and III-V compounds. *Physical Review* **141**, 767 (1966).

374 Bakhchieva, S., Kekelidze, N. & Kekua, M. Thermal conductivity of germanium doped with silicon, tin, and aluminium.

physica status solidi (a) **83**, 139-145 (1984).

375 Steigmeier, E. F. & Kudman, I. Thermal Conductivity of III-V Compounds at High Temperatures. *Physical Review* **132**, 508-512, doi:10.1103/PhysRev.132.508 (1963).

376 Sparavigna, A. Lattice thermal conductivity in cubic silicon carbide. *Phys. Rev. B* **66**, doi:10.1103/PhysRevB.66.174301 (2002).

377 Sanjurjo, J. A., López-Cruz, E., Vogl, P. & Cardona, M. Dependence on volume of the phonon frequencies and the ir effective charges of several III-V semiconductors. *Phys. Rev. B* **28**, 4579-4584, doi:10.1103/PhysRevB.28.4579 (1983).

378 Abendroth, B., Gago, R., Eichhorn, F. & Möller, W. X-ray diffraction study of stress relaxation in cubic boron nitride films grown with simultaneous medium-energy ion bombardment. *Appl. Phys. Lett.* **85**, 5905-5907, doi:10.1063/1.1836868 (2004).

379 Slack, G. A. & Bartram, S. F. Thermal expansion of some diamondlike crystals. *J. Appl. Phys.* **46**, 89-98, doi:10.1063/1.321373 (1975).

380 J.K.D. Verma & Aggarwal, M. D. Grüneisen constant of some semiconductors. *Solid State Commun.* **8**, 1929-1931 (1970).

381 Benkabou, F., Chikr, C., Aourag, H., Becker, P. J. & Certier, M. Atomistic study of zinc-blende BAs from molecular dynamics. *Phys. Lett. A* **252**, 71-76, doi:Doi 10.1016/S0375-9601(99)80004-8 (1999).

382 Venkateswaran, U. D., Cui, L. J., Weinstein, B. A. & Chambers, F. A. Forward and reverse high-pressure transitions in bulklike AlAs and GaAs epilayers. *Phys Rev B Condens Matter* **45**, 9237-9247, doi:10.1103/physrevb.45.9237 (1992).

383 P., S. D. Lattice thermal conductivity of semiconductors: A chemical bond approach. *J. Phys. Chem. Solids* **31(1)**, 19-40 (1970).

384 Afromowitz, M. A. Thermal conductivity of Ga1−xAlxAs alloys. *J. Appl. Phys.* **44**, 1292-1294, doi:10.1063/1.1662342 (1973).

385 Weinstein, B. A. & Zallen, R. Pressure-Raman Effects in Covalent and Molecular-Solids. *Top. Appl. Phys.* **54**, 462-527 (1984).

386 Slack, G. A., Tanzilli, R. A., Pohl, R. & Vandersande, J. The intrinsic thermal conductivity of AIN. *J. Phys. Chem. Solids* **48**, 641-647 (1987).

387 Rimai, D. S. & Sladek, R. J. Elastic-Moduli and Mode Gammas of Gap - Their Relationship to Those of Other Isomorphic Crystals and the High-Pressure Structural-Electrical Transition. *Solid State Commun.* **30**, 591-594, doi:Doi 10.1016/0038-1098(79)90102-9 (1979).

388 Soma, T., Satoh, J. & Matsuo, H. Thermal-Expansion Coefficient of Gaas and Inp. *Solid State Commun.* **42**, 889-892, doi:Doi 10.1016/0038-1098(82)90233-2 (1982).

389 Herres, N. *et al.* Effect of interfacial bonding on the structural and vibrational properties of InAs/GaSb superlattices. *Phys Rev B Condens Matter* **53**, 15688-15705, doi:10.1103/physrevb.53.15688 (1996).

390 Tronc, P., Reid, B., Maciejko, R., Leclercq, J. L. & Lazzari, J. L. Optical-Properties and Fluctuations of Composition in Ga0.77in0.23as0.19sb0.81 Alloys. *Solid State Commun.* **85**, 177-181, doi:Doi 10.1016/0038-1098(93)90370-3 (1993).

391 Krukowski, S. *et al.* Thermal properties of indium nitride. *J. Phys. Chem. Solids* **59**, 289-295 (1998).

392 Andrés Cantarero & Hernández-Calderón, I. High Pressure Semiconductor Physics. *physica status solidi (b)* **252**, 651-652, doi:10.1002/pssb.201570326 (2015).

393 Haruna K, Maeta H & Ohashi K, e. a. The thermal expansion coefficient and Gruneisen parameter of InP crystal at low temperatures. *Journal of Physics C: Solid State Physics* **20(32)**, 5275 (1987).

394 Aliev, S., NASHELSKII, A. & Shalyt, S. Thermal conductivity and thermoelectric power of n-type indium phosphide at low temperatures(Temperature dependence of heat conductivity and thermal emf in n-type InP). *SOVIET PHYSICS-SOLID STATE* **7**, 1287 (1965).

395 Orlova, N. Variation of phonon dispersion curves with temperature in indium arsenide measured by X‐ray thermal diffuse scattering. *physica status solidi (b)* **119**, 541-546 (1983).

396 Sushil, K. Volume dependence of isothermal bulk modulus and thermal expansivity of MgO. *Physica B: Condensed Matter* **367**, 114-123, doi:10.1016/j.physb.2005.06.004 (2005).

397 Lu, L.-Y., Cheng, Y., Chen, X.-R. & Zhu, J. Thermodynamic properties of MgO under high pressure from first-principles calculations. *Physica B: Condensed Matter* **370**, 236-242, doi:10.1016/j.physb.2005.09.017 (2005).

398 W.A. Fischer & Janke, D. Electrolytic deoxidation of liquid metals at 1600 ℃. *Scripta Metallurgica* **6**, 923-928 (1972).

399 Beals, R. J. & Cook, R. L. Directional Dilatation of Crystal Lattices at Elevated Temperatures. *J. Am. Ceram. Soc.* **40**, 279-284, doi:10.1111/j.1151-2916.1957.tb12620.x (2006).

400 Zollweg, R. J. X-Ray Lattice Constant of Barium Oxide. *Physical Review* **100**, 671-673, doi:10.1103/PhysRev.100.671 (1955).

401 Slack, G. A. Thermal Conductivity of II-VI Compounds and Phonon Scattering byFe2+Impurities. *Phys. Rev. B* **6**, 3791-3800, doi:10.1103/PhysRevB.6.3791 (1972).

402 Soma, T. Thermal-Expansion and Lattice-Dynamics under Pressure of Zns, Znse and Znte. *Solid State Commun.* **34**, 927-932, doi:Doi 10.1016/0038-1098(80)91100-X (1980).

403 Schorr, S. & Sheptyakov, D. Low-temperature thermal expansion in sphalerite-type and chalcopyrite-type multinary semiconductors. *J. Phys.: Condens. Matter* **20**, doi:10.1088/0953-8984/20/10/104245 (2008).

404 Koumelis, C. N. The thermal expansion coefficient of chromium in the temperature region of 3 to 80 ℃. *Physica Status Solidi (a)* **19**, K65-K69, doi:10.1002/pssa.2210190157 (1973).

405 Straumanis, M. E. & Aka, E. Z. Lattice Parameters, Coefficients of Thermal Expansion, and Atomic Weights of Purest Silicon and Germanium. *J. Appl. Phys.* **23**, 330-334, doi:10.1063/1.1702202 (1952).

406 H.P. Singh & Dayal, B. Lattice parameters of cadmium oxide at elevated temperatures. *Solid State Commun.* **7**, 725-726 (1969).

407 Klemens, P. *Thermal Conductivity 14*. (Springer, 2013).

408 M.G. Williams, R.D. Tomlinson & Hampshire, M. J. X-ray determination of the lattice parameters and thermal expansion of cadmium telluride in the temperature range 20–420 ℃. *Solid State Commun.* **7**, 1831-1832 (1969).

409 Whitsett, C. R., Nelson, D. A., Broerman, J. G. & Paxhia, E. C. Lattice Thermal Conductivity of Mercury Selenide. *Phys. Rev. B* **7**, 4625-4640, doi:10.1103/PhysRevB.7.4625 (1973).

410 Takahashi, T. & Kikuchi, T. Porosity dependence on thermal diffusivity and thermal conductivity of lithium oxide Li2O from 200 to 900 C. *J. Nucl. Mater.* **91**, 93-102 (1980).

411 Lide, D. R. E. *Handbook of Chemistry and Physics: CRC Handbook*. (CRC Press, 1993).

412 Slack, G. A. Thermal Conductivity of MgO,Al2O3, MgAl2O4, andFe3O4Crystals from 3 ° to 300 °K. *Physical Review* **126**, 427-441, doi:10.1103/PhysRev.126.427 (1962).

413 Bruce, R. H. & Cannell, D. S. Specific heat ofCr2O3near the Néel temperature. *Phys. Rev. B* **15**, 4451-4459, doi:10.1103/PhysRevB.15.4451 (1977).

414 Marinelli, M. *et al.* Critical behavior of thermal diffusivity and thermal conductivity ofCr2O3at the Néel transition. *Phys. Rev. B* **49**, 4356-4359, doi:10.1103/PhysRevB.49.4356 (1994).

415 Horai, K.-i. Thermal conductivity of rock-forming minerals. *Journal of Geophysical Research* **76**, 1278-1308, doi:10.1029/JB076i005p01278 (1971).

416 Víllora, E. G., Shimamura, K., Ujiie, T. & Aoki, K. Electrical conductivity and lattice expansion of β-Ga2O3 below room temperature. *Appl. Phys. Lett.* **92**, 202118, doi:10.1063/1.2910770 (2008).

417 Li, J.-G., Ikegami, T. & Mori, T. Fabrication of transparent Sc2O3 ceramics with powders thermally pyrolyzed from sulfate. *J. Mater. Res.* **18**, 1816-1822, doi:10.1557/jmr.2003.0252 (2011).

418 Masao Kuriyama & Hosoya, S. X-ray Measurement of Scattering Factors of Manganese and Oxygen Atoms in Manganous Oxide. *J. Phys. Soc. Jpn.* **17**, 1022-1029 (1962).

419 Slack, G. & Newman, R. Thermal conductivity of MnO and NiO. *Phys. Rev. Lett.* **1**, 359 (1958).

420 Zhang, Y. *et al.* Microstructures and properties of high-entropy alloys. *Prog. Mater Sci.* **61**, 1-93, doi:10.1016/j.pmatsci.2013.10.001 (2014).

421 Lewis, F. & Saunders, N. The thermal conductivity of NiO and CoO at the Neel temperature. *Journal of Physics C: Solid State Physics* **6**, 2525 (1973).

422 Ren, Y. *et al.* Synergistic effect by Na doping and S substitution to high thermoelectric performance of p-type MnTe.

Journal of Materials Chemistry C (2017).
423 Turkes P, P. C., Helbig R. Thermal conductivity of SnO2 single crystals. *Journal of Physics C: Solid State Physics* **13(26)**, 4941 (1980).
424 Khélia, C., Boubaker, K., Ben Nasrallah, T., Amlouk, M. & Belgacem, S. Morphological and thermal properties of β-SnS2 sprayed thin films using Boubaker polynomials expansion. *J. Alloys Compd.* **477**, 461-467, doi:10.1016/j.jallcom.2008.10.051 (2009).
425 Wang, H., Gao, Y. & Liu, G. Anisotropic phonon transport and lattice thermal conductivities in tin dichalcogenides SnS 2 and SnSe 2. *RSC Advances* **7**, 8098-8105 (2017).
426 Busch, G., Fröhlich, C., Hulliger, F. & Steigmeier, E. Struktur, elektrische und thermoelektrische Eigenschaften von SnSe2. *Helv. Phys. Acta* **34**, 359-368 (1961).
427 Ibrahim, D. *et al.* Reinvestigation of the thermal properties of single-crystalline SnSe. *Appl. Phys. Lett.* **110**, 032103, doi:10.1063/1.4974348 (2017).
428 Jin, M. *et al.* Growth and characterization of large size undoped p -type SnSe single crystal by Horizontal Bridgman method. *J. Alloys Compd.* **712**, 857-862, doi:10.1016/j.jallcom.2017.04.110 (2017).
429 Zhao, L. *et al.* Ultralow thermal conductivity and high thermoelectric figure of merit in SnSe crystals. *Nature* **508**, 373-377, doi:10.1038/nature13184 (2014).
430 Y. Sternberg, N. Yellin, S. Cohen & Dor, L. B. Lattice matching and thermal expansion in the Pb-Sn-Te system. *J. Solid State Chem.* **32**, 364-367 (1982).
431 T. F. Smith & White, G. K. The low-temperature thermal expansion and Gruneisen parameters of some tetrahedrallybonded solids. *J. Phys. C: Solid State Phys.* **8** 2031 (1975).
432 Morelli, D. T., Jovovic, V. & Heremans, J. P. Intrinsically Minimal Thermal Conductivity in Cubic I-V-VI_2 Semiconductors. *Phys. Rev. Lett.* **101**, 035901 (2008).
433 Okhotin, A., Krestovnikov, A., Aivazov, A. & Pushkarskii, A. Thermal Conductivity of GeS and GeSe. *physica status solidi (b)* **31**, 485-487 (1969).
434 C., F. N. Properties of gallium selenide single crystal. *Prog. Cryst. Growth Charact. Mater.* **28(4)**, 275-353 (1994).
435 Stutius, W. Diamagnetic susceptibility of superionic conductors CuI and AgI. *Solid State Commun.* **18**, 1275-1278 (1976).
436 T. Sauder, A. Daunois, J.L. Deiss & Merle, J. C. Effects of uniaxial stress on the excitons in single crystals of CuI: Comparison with thin films. *Solid State Commun.* **51**, 323-326 (1984).
437 Anis-ur-Rehman M, M. A. Measurement of thermal transport properties with an improved transient plane source technique. *Int. J. Thermophys.* **24(3)**, 867-883 (2003).
438 J.L Tallon & Buckley, R. G. The fast-ion transition in FCC silver iodide. *Solid State Commun.* **47**, 563-566 (1983).
439 de Göer, A., Locatelli, M. & Nicolau, I. Low-temperature heat transport in α-HgI2 single crystals. *J. Phys. Chem. Solids* **43**, 311-317 (1982).
440 Burger, A., Morgan, S., Henderson, D., Silberman, E. & Nason, D. Thermal diffusivity of α ‐ mercuric iodide. *J. Appl. Phys.* **69**, 722-725 (1991).
441 Silva, T. *et al.* Thermal diffusivity of lead iodide. *J. Appl. Phys.* **83**, 6193-6195 (1998).
442 Palchoudhuri S, B. G. K. The thermal conductivity of some fluorite structured compounds. *Solid State Commun.* **70(4)**, 475-478 (1989).
443 Spitzer, D. Lattice thermal conductivity of semiconductors: A chemical bond approach. *J. Phys. Chem. Solids* **31**, 19-40 (1970).
444 Smirnov, I. & Oskotski, V. Thermal conductivity of rare earth compounds. *Handbook on the Physics and Chemistry of Rare Earths* **16**, 107-224 (1993).
445 Martin, J. Thermal conductivity of Mg2Si, Mg2Ge and Mg2Sn. *J. Phys. Chem. Solids* **33**, 1139-1148 (1972).
446 Tani, J.-i. & Kido, H. Thermoelectric properties of Sb-doped Mg2Si semiconductors. *Intermetallics* **15**, 1202-1207, doi:10.1016/j.intermet.2007.02.009 (2007).
447 Reimann, K. & Syassen, K. Raman scattering and photoluminescence in Cu 2 O under hydrostatic pressure. *Phys. Rev. B* **39**, 11113 (1989).

448 Chen, X., Parker, D., Du, M.-H. & Singh, D. J. Potential thermoelectric performance of hole-doped Cu2O. *New Journal of Physics* **15**, 043029 (2013).
449 Liu, H. *et al.* Ultrahigh Thermoelectric Performance by Electron and Phonon Critical Scattering in Cu2Se1-xIx. *Adv. Mater.* **25**, 6607-6612, doi:10.1002/adma.201302660 (2013).
450 Day, T. *et al.* Evaluating the potential for high thermoelectric efficiency of silver selenide. *Journal of Materials Chemistry C* **1**, 7568-7573 (2013).
451 Brockway, L., Vasiraju, V., Asayesh-Ardakani, H., Shahbazian-Yassar, R. & Vaddiraju, S. Thermoelectric properties of large-scale Zn3P2 nanowire assemblies. *Nanotechnology* **25**, 145401, doi:10.1088/0957-4484/25/14/145401 (2014).
452 Masumoto, K., Isomura, S. & Sasaki, K. Physical and electronic properties of semiconducting solid solutions of the Cd3P2–Zn3P2 system. *physica status solidi (a)* **6**, 515-523 (1971).
453 Spitzer, D. P., Castellion, G. A. & Haacke, G. Anomalous Thermal Conductivity of Cd3As2 and the Cd3As2− Zn3As2 Alloys. *J. Appl. Phys.* **37**, 3795-3801 (1966).
454 Toman, K. The structure of ZnSb-CdSb alloys. *J. Phys. Chem. Solids* **11**, 342 (1959).
455 White, G. Thermal expansion of trigonal elements at low temperatures: As, Sb and Bi. *Journal of Physics C: Solid State Physics* **5**, 2731 (1972).
456 Heremans, J., Issi, J.-P., Rashid, A. & Saunders, G. Electrical and thermal transport properties of arsenic. *Journal of Physics C: Solid State Physics* **10**, 4511 (1977).
457 Morelli, D. T. & Uher, C. Thermal conductivity of arsenic single crystals from 2 to 300 K. *Phys. Rev. B* **28**, 4242-4246, doi:10.1103/PhysRevB.28.4242 (1983).
458 Völklein, F. & Kessler, E. Thermal conductivity and thermoelectric figure of merit of thin antimony films. *physica status solidi (b)* **158**, 521-529 (1990).
459 Grosse, R., Krause, P., Meissner, M. & Tausend, A. The coefficients of thermal expansion and the Gruneisen functions of trigonal and amorphous selenium in the temperature range between 10K and 300K. *Journal of Physics C: Solid State Physics* **11**, 45 (1978).
460 Hortal, M. & Leadbetter, A. The low temperature thermal expansion and Gruneisen functions of tellurium. *Journal of Physics C: Solid State Physics* **5**, 2129 (1972).
461 Bauer E, P. C., Berger S, et al. Thermal conductivity of superconducting MgB2. *J. Phys.: Condens. Matter* **13(22)**, L487 (2001).
462 Basu, B., Raju, G. & Suri, A. Processing and properties of monolithic TiB2 based materials. *Int. Mater. Rev.* **51**, 352-374 (2006).
463 Hill, R. F. & Supancic, P. H. Thermal conductivity of platelet - filled polymer composites. *J. Am. Ceram. Soc.* **85**, 851-857 (2002).
464 Guo, S.-Q., Kagawa, Y., Nishimura, T. & Tanaka, H. Pressureless sintering and physical properties of ZrB2-based composites with ZrSi2 additive. *Scripta Mater.* **58**, 579-582, doi:10.1016/j.scriptamat.2007.11.019 (2008).
465 Guo, S.-Q. Densification of ZrB2-based composites and their mechanical and physical properties: A review. *J. Eur. Ceram. Soc.* **29**, 995-1011, doi:10.1016/j.jeurceramsoc.2008.11.008 (2009).
466 Opeka M M, T. I. G., Wuchina E J, et al. Mechanical, thermal, and oxidation properties of refractory hafnium and zirconium compounds. *J. Eur. Ceram. Soc.* **19(13)**, 2405-2414 (1999).
467 Takeda, M., Fukuda, T. & Miura, T. in *Thermoelectrics, 2002. Proceedings ICT'02. Twenty-First International Conference on.* 173-176 (IEEE).
468 Gürsoy, M., Takeda, M. & Albert, B. High-pressure densified solid solutions of alkaline earth hexaborides (Ca/Sr, Ca/Ba, Sr/Ba) and their high-temperature thermoelectric properties. *J. Solid State Chem.* **221**, 191-195, doi:10.1016/j.jssc.2014.10.001 (2015).
469 Peysson, Y., Ayache, C., Salce, B., Kunii, S. & Kasuya, T. Thermal conductivity of CeB6 and LaB6. *J. Magn. Magn. Mater.* **59**, 33-40 (1986).
470 Sera, M., Kobayashi, S., Hiroi, M., Kobayashi, N. & Kunii, S. Thermal conductivity of R B 6 (R= C e, P r, N d, S m, G d) single crystals. *Phys. Rev. B* **54**, R5207 (1996).

471 Popov, P. A., Novikov, V. V., Sidorov, A. A. & Maksimenko, E. V. Thermal conductivity of LaB6 and SmB6 in the range 6–300 K. *Inorg. Mater.* **43**, 1187-1191, doi:10.1134/s0020168507110064 (2007).

472 Nolas, G. S., Slack, G. A. & Schujman, S. B. Semiconductor clathrates: A phonon glass electron crystal material with potential for thermoelectric applications. *Semiconductors and semimetals* **69**, 255-300 (2001).

473 Odintsov V V, L. M. I., L'vov S N. Thermal conductivity of metal dodecaborides with a UB12 structure. *Soviet Atomic Energy* **35(3)**, 834-835 (1973).

474 Misiorek, H., Mucha, J., Jezowski, A., Paderno, Y. & Shitsevalova, N. Thermal conductivity of rare-earth element dodecaborides. *Journal of Physics: Condensed Matter* **7**, 8927 (1995).

475 Iga, F. *et al.* Thermoelectric properties of the Kondo semiconductor: Yb 1− x Lu x B 12. *J. Magn. Magn. Mater.* **226**, 137-138 (2001).

476 Cui, G.-l. & Yu, R.-l. Volume and pressure dependence of Grüneisen parameter γ for solids at high temperatures. *Physica B: Condensed Matter* **390**, 220-224, doi:10.1016/j.physb.2006.08.034 (2007).

477 Gambino, R. J. & Seiden, P. E. Correlation of the Superconducting Transition Temperature with an Empirical Pseudopotential Determined from Atomic Spectra. *Phys. Rev. B* **2**, 3571-3577, doi:10.1103/PhysRevB.2.3571 (1970).

478 Jindal, V. K. & Pathak, K. N. Thermal expansion of sodium and potassium. *Phys. Rev. B* **14**, 3704-3705, doi:10.1103/PhysRevB.14.3704 (1976).

479 Pathak, P. D. & Desai, R. J. Thermal expansion of magnesium and temperature variation of negative second moment of its frequency spectrum. *Physica Status Solidi (a)* **66**, K179-K182, doi:10.1002/pssa.2210660271 (1981).

480 Zoli, M. & Bortolani, V. Thermodynamic Properties of Fcc Metals - Cu and Al. *J Phys-Condens Mat* **2**, 525-539, doi:Doi 10.1088/0953-8984/2/3/003 (1990).

481 Swartz, K. D., Chua, W. B. & Elbaum, C. Third-Order Elastic Constants of Tin and of a Tin-Indium Alloy. *Phys. Rev. B* **6**, 426-435, doi:10.1103/PhysRevB.6.426 (1972).

482 Kazanc, S., Çiftci, Y. Ö., Çolakoğlu, K. & Ozgen, S. Temperature and pressure dependence of the some elastic and lattice dynamical properties of copper: a molecular dynamics study. *Physica B: Condensed Matter* **381**, 96-102, doi:10.1016/j.physb.2005.12.259 (2006).

483 Sirota, N. N. & Zhabko, T. E. X-ray study of the anisotropy of thermal properties in titanium. *Physica Status Solidi (a)* **63**, K211-K215, doi:10.1002/pssa.2210630266 (1981).

484 <Zr-a1.pdf>.

485 Delaire, O. *et al.* Electron-phonon interactions and high-temperature thermodynamics of vanadium and its alloys. *Phys. Rev. B* **77**, doi:10.1103/PhysRevB.77.214112 (2008).

486 Lucas, M. S. *et al.* Effects of composition, temperature, and magnetism on phonons in bcc Fe-V alloys. *Phys. Rev. B* **82**, doi:10.1103/PhysRevB.82.144306 (2010).

487 B.B. Argent & Milne, G. J. C. The physical properties of niobium, tantalum, molybdenum and tungsten. *Journal of the Less Common Metals* **2**, 154-162 (1960).

488 Mukhopadhyay, S. *et al.* The curious case of cuprous chloride: Giant thermal resistance and anharmonic quasiparticle spectra driven by dispersion nesting. *Phys. Rev. B* **96**, doi:10.1103/PhysRevB.96.100301 (2017).

489 Adams, J. J., Agosta, D. S., Leisure, R. G. & Ledbetter, H. Elastic constants of monocrystal iron from 3 to500K. *J. Appl. Phys.* **100**, doi:10.1063/1.2365714 (2006).

490 White, G. K. & Andrikidis, C. Thermal expansion of chromium at high temperature. *Phys. Rev. B* **53**, 8145-8147, doi:DOI 10.1103/PhysRevB.53.8145 (1996).

491 Pathak, P. D. & Vasavada, N. G. Thermal expansion of LiF by X-ray diffraction and the temperature variation of its frequency spectrum. *Acta Crystallographica Section A* **28**, 30-33, doi:10.1107/s0567739472000063 (1972).

492 Yousuke Watanabe, A. S., Toshio Sakurai. Lattice Parameter and Thermal Expansion Measurements of a LiF(001) Surface by He-Atom Beam Diffraction Method. *J. Phys. Soc. Jpn.* **66**, 649-652 (1997).

493 Reinhard Boehler, G. C. K. Thermal expansion of LiF at high pressures. *J. Phys. Chem. Solids* **41**, 1019-1022 (1980).

494 Yagi, T. Experimental determination of thermal expansivity of several alkali halides at high pressures. *J. Phys. Chem. Solids* **39**, 563-571 (1978).

495 Rapp, J. E. & Merchant, H. D. Thermal expansion of alkali halides from 70 to 570 K. *J. Appl. Phys.* **44**, 3919-3923, doi:10.1063/1.1662872 (1973).

496 Hakansson, B. & Ross, R. G. Thermal-Conductivity and Heat-Capacity of Solid Libr and Rbf under Pressure. *J Phys-Condens Mat* **1**, 3977-3985, doi:Doi 10.1088/0953-8984/1/25/009 (1989).

497 Reinhard Boehler, G. C. K. Equation of state of sodium chloride up to 32 kbar and 500 ℃. *J. Phys. Chem. Solids* **41**, 517-523 (1980).

498 B. Håkansson, P. A. Thermal conductivity and heat capacity of solid NaCl and NaI under pressure. *J. Phys. Chem. Solids* **47**, 355-362 (1986).

499 Kai Wang & Reeber, R. R. High temperature thermal expansion of alkali halides. *J. Phys. Chem. Solids* **56**, 895-900 (1995).

500 P. D. Pathak, N. G. V. Thermal expansion of NaCl, KCl and CsBr by X-ray diffraction and the law of corresponding states. *Acta Cryst.* **A26**, 655-658 (1970).

501 A.K. Gupta & Rao, K. V. Thermal expansion measurements for estimating vacancy concentration in X-ray irradiated KCl single crystals. *Solid State Commun.* **34**, 655-657 (1980).

502 Bailey, A. C. & Yates, B. The low temperature thermal expansion and related thermodynamic properties of alkali halides with a caesium chloride structure. *The Philosophical Magazine: A Journal of Theoretical Experimental and Applied Physics* **16**, 1241-1248, doi:10.1080/14786436708229973 (2006).

503 Deshpande, V. T. Thermal expansion of sodium fluoride and sodium bromide. *Acta Cryst.* **14**, 794 (1961).

504 John W. Schwartz, C. T. W. Thermal Conductivity of Some Alkali Halides Containing Divalent Impurities. I. Phonon Resonances. *Phys. Rev.* **155**, 959 (1967).

505 R G Ross, P. A., B Sundqvist, G Backstrom. Thermal conductivity of solids and liquids under pressure. *Rep. Prog. Phys.* **47**, 1347 (1984).

506 J. W. Johnson, P. A. Agron & Bredig, M. A. Molar Volume and Structure of Solid and Molten Cesium Halides. *J. Am. Chem. Soc.* **77**, 2734–2737 (1955).

507 COLELL, R. Reply to Post's comments on my paper Multiple Diffraction of X-rays and the phase problem. Computational procedures and comparison with experiment. *Acta Cryst.* **A31**, 155 (1975).

508 Khan, A. A. Ionic size effects on the thermal expansion coefficient in NaCl-type alkali halides. *Acta Cryst.* **A30**, 105-106 (1974).

509 Sirdeshmukh, D. B. Effective Ionic Charge and the Thermal Expansion of the Alkali Halide Crystals. *J. Chem. Phys.* **45**, 2333–2334 (1966).

510 Andersson, P. Thermal conductivity under pressure and through phase transitions in solid alkali halides. I. Experimental results for KCl, KBr, KI, RbCl, RbBr and RbI. *J. Phys. C: Solid State Phys.* **18**, 3943 (1985).

511 D Gerlich, P. A. Temperature and pressure effects on the thermal conductivity and heat capacity of CsCl, CsBr and CsI. *J. Phys. C: Solid State Phys.* **15**, 5211 (1982).
